# Scientists have favorable opinions on immunity certificates but raise concerns regarding fairness and inequality

**DOI:** 10.1038/s41598-021-93148-1

**Published:** 2021-07-07

**Authors:** Iván Aranzales, Ho Fai Chan, Reiner Eichenberger, Rainer Hegselmann, David Stadelmann, Benno Torgler

**Affiliations:** 1 School of Economics and Finance, Queensland University of Technology, Brisbane, QLD Australia; 2 Centre for Behavioural Economics, Society and Technology (BEST), Brisbane, QLD Australia; 3 University of Fribourg, Fribourg, Switzerland; 4 CREMA ‐ Center for Research in Economics, Management and the Arts, Basel, Switzerland; 5 Frankfurt School of Finance & Management, Frankfurt, Germany; 6 University of Bayreuth, Bayreuth, Germany; 7 IREF ‐ Institute for Research in Economic and Fiscal Issues, Paris, France

**Keywords:** Psychology, Health care

## Abstract

During the first wave of the COVID-19 pandemic, we collected over 12,000 responses from a survey of scientists, who were asked to express their opinions on immunity certificates (also called “immunity passports”) as a potential instrument to lessen the impact of the crisis. Overall, we find that scientists perceive immunity certificates as favorable for public health (50.2%) and the state of the economy (54.4%) while one-fifth (19.1%) and one-sixth (15.4%) disagree. Scientists stipulate some concerns about fairness (36.5%) and inequality (22.4%) arising from implementation of immunity certification. We find some smaller differences among scientific fields, particularly between health scientists and social scientists, with the latter being slightly more positive about the effect of immunity certification. Scholars in the United States, including health scientists, are more likely to view the immunity certificates favorably and mention fewer concerns about this policy’s effect on fairness and inequality. Female scholars are significantly less in favor of immunity certificates, while scientists with more conservative political views hold more favorable opinions. Our results reveal that given the uncertainties during an early phase of a pandemic, scientists see scope for immunity certification to lessen the general societal impacts of the crisis.

## Introduction

COVID-19-related uncertainty and policy reactions caused major disruptions in almost every country, with the US and several Western European nations experiencing high levels of infections and societal fallouts^1,2^. With global eradication seen as unlikely, convalescent, and later vaccine induced immunity gained prominence as the path out of the crisis. In December 2020, the vaccine made by Pfizer and BioNTech became the first fully-tested immunization approved for emergency use^3^ and developers of several vaccines announced excellent results. Insights are being generated over time through careful longitudinal studies on safety, immunogenicity, and protection rate^4^. While the duration of vaccine-acquired immunity is yet uncertain, neutralizing antibodies after infection and induced immunological memory reactions to SARS-CoV-2 have been shown to persist for several months at least^5,6^. As the number of people with convalescent-induced (infection induced) and vaccine-induced immunity continues to increase, the utility of strict infection control measures decreases. The economic and social costs of such measures were high^7^.

Immunity certificates for convalescents were discussed by scientists early during the pandemic^8–11^ and they received policy attention in several countries. Such certificates were mostly intended as a transitory tool to reduce the total costs of the pandemic^12^; to allow individuals to resume their usual activities and interacting with others who were isolated or whose relationships were interrupted^13^; and to facilitate travel, taking into account that immunity passports were already used in public health and travel medicine^14^. While immunity certificates for convalescents were not introduced prior to the advent of vaccines, the introduction of Israel’s “Green Pass”—intended for those vaccinated or those who have already contracted the disease—prompted a surge of calls from policy makers for implementation in early 2021. The President of the European Commission, Ursula von der Leyen, presented a legislative proposal for a type of European “Green Pass” that would include information on whether there is proof that a person has been vaccinated, information on COVID19 recovery, or whether a person was tested for infection.

Acceptance of COVID-19 vaccines may differ among individuals. Existing evidence points to the usefulness of focusing on prosocial concerns when motivating vaccination uptake^15^. A global survey assessing potential acceptance of a COVID-19 vaccine indicates country-level differences in acceptance rates ranging from less than 55% in Russia to 90% in China^16^. Another study using European data reports that people’s willingness to vaccinate ranges from 30% (Hungary) to 80% (Denmark)^17^. Several individual-level differences are associated with the willingness to receive a vaccine^18^. Organized campaigns by vaccine-hesitant groups promote beliefs that vaccinations are unsafe via social media, leading to vaccine hesitancy; a large proportion of the content shared about vaccines on popular social media sites are anti-vaccination messages^19^. Thus, vaccine refusal is seen as a significant problem^17^. Individual acceptance of vaccination may also be negatively affected by increasing immunity levels in the population, as the incentive to free ride on others’ vaccinations grows^20,21^. This raises the question of whether immunity certificates would offer further motivation and positive incentive to vaccinate if they allow more individual freedom of movement and restoration of liberties, thereby empowering and motivating individuals to contribute to the common good^8^. Enforced measures have been shown to crowd out voluntary support for COVID-19 policies^22^.

These considerations make the question of immunity certificates an ongoing and important policy topic in addressing the impacts of the COVID-19 crisis. An even more critical aspect may be whether immunity certificates could serve more generally as a policy instrument to reduce the costs of future pandemics, even when vaccine-induced immunity does not become available as quickly as it has during this pandemic. Seeking more clarity on the role of immunity certificates as a tool to mitigate some of the health, social, and economic impacts of pandemics, we conducted a survey of 12,738 scientists between May 4 and June 3, 2020. Early uncertainty about the duration and strength of convalescent immunity and the doubts regarding the potential of vaccine development make our survey interesting for understanding the scope of immunity certification in the initial stages of such a crisis. While future crises may be of a different structure, the knowledge that some immunity may be conferred by infection could make immunity certificates a helpful policy tool in specific circumstances.

## Background and context

First and early evidence regarding the direct health effects of COVID-19 indicated that the high-risk group was largely comprised of elderly people and people with pre-existing medical conditions^23,24^. In March 2020, the World Health Organization noted that most patients (80%) experienced mild illness, based on data from China^25^. The number of asymptomatic cases was unknown but stated to be relevant for evaluation of policies and the ultimate severity of the pandemic^26^.

Although evidence cannot exist for a new type of virus, the probability of contracting the same illness from the virus a second time within a few months or even years was considered to be small compared to a first occurrence of the illness. Numerous experts such as Peter Doherty—recipient of the Nobel Prize—suggested in media reports that even if there was a reinfection, prior infection would give an individual a level of immunity, allowing them to recover quickly^27^. To some degree this was the—now seen as largely correct—prior belief.

Pandemics and the reactions to them increase the general problem of scarcity that always exists in society. The possible immunity of convalescents could make them each individually and as a group a valuable resource, and such resources multiply as the number of convalescents increases^12^. Moreover, from a point of view of freedom, it is not easy to justify wide-ranging restrictions on this group of immune people. To make individual immunity useful during the crisis and for the individual, a certification that a person has contracted and recovered from COVID-19 would be vital. However, certification policies are connected with questions about individual freedom, public health, economic benefits, fairness, and inequality; all issues that were controversially discussed by the scientific community^9,10^. In a statement on April 24, 2020 the World Health Orgazination suggested that “there is not enough evidence about the effectiveness of antibody-mediated immunity to guarantee the accuracy of an ‘immunity passport’ or ‘risk-free certificate’” and the organization stated that such certificates may increase the risks of transmission^28^. Immunity certificates might create incentives for self-infection if they are associated with large benefits. Evidence on robust immune responses after infection accumulated over time^5,29,30^, but with the advent of vaccines, widespread immunity came in reach at low risks. With vaccine availability in early 2021, Israel became the first country to introduce a “Green Pass” for vaccinated and previously infected individuals.

To evaluate opinions regarding immunity certificates as a policy tool, we designed a survey in late March and April 2020. The aim of the survey was to gather information on the acceptance of immunity certificates among scientists during a phase of uncertainty about the disease and uncertainty about the precise immune reactions, such that our conclusions could be useful when related discussions emerge during future crises. Data were collected between May 4 and June 3, 2020; i.e., still during the first wave in most countries and where attention was heavily focused on COVID-19. Responses were gathered via the SurveyMonkey platform from scholars appearing in Scopus (see Method section). We also included scholars from the bibliographic database *RePEc* to increase the representation of social scientists (excluding these entries from the analysis does not influence our main conclusions, see *Appendix SI*). The design allowed us to collect the opinions of scientists across 37 subfields and 63 countries (see Supplementary Fig. 1). We gathered a sample of 213,648 email addresses from journal publications. The response rate was 13.9% based on emails opened (see Supplementary Table 16) and 12,738 scientists eventually concluded the survey. We aimed at a careful understanding and mapping of scholarly positions to establish the consensus of scientists’ opinions on different aspects of immunity certification. Survey respondents could skip any questions they did not want to answer. Ethical approval for the survey and the data collection was given on April 23, 2020 by the Ethics Commission of the Frankfurt School of Finance & Management (Frankfurt, Germany).

Humans are boundedly rational beings^31^ and subject to emotions^32^ such as fear. They react to the complexity of the environment^33^, which affects their level of trust in the government^34^. Limited information on contextual factors or dynamic changes may not allow for clear ideas about risks and facts in uncommon situations such as pandemics. The survey was addressed to scientists, and we explicitly address respondents in their function as a scientist. Being highly educated, being trained in rational thinking and dealing with aspects of uncertainty, scientists have the potential to represent an interesting group when exploring opinions regarding immunity certificates. Uncertainty is a core problem in pandemics, and historically pandemics have often caught governments and authorities unprepared and flat-footed, leading to confusion and improvisation^35^. Scientists are also aware of how surveys work and deal daily with hypothetical situations. They are trained to think about social responsibility and therefore beyond personal interest, with a natural concern for understanding how people make choices. While they are not immune to bias and do not possess all information, they are usually better aware of the state of affairs than the wider public. Moreover, during crises they often inform policymakers and the public regarding scenarios, policy options, or potential trade-offs. In particular, governments can be heavily dependent on experts and scientific advice during crises due to their need for rapid responses^36^. During pandemics such as COVID-19 that represent a massive global health crisis, scientists can help policy-makers, leaders, decision-makers and the public in general to better understand how to handle and manage potential threats, how to find solutions, how to navigate different contexts (e.g., social or cultural), or how to align individual or collective interests while also providing effective science communication^37^. Their attitudes towards policy tools can therefore shape how society copes with such crises. Policy-makers have a variety of tools available to deal with crises. It is therefore valuable to understand the acceptance of—and consensus on—available tools among scientists. Such an inquiry contributes to cognitive processes of learning to cope with new situations, feeding into the discussion process in which insights, attitudes, and preferences are exchanged. It provides a way of understanding not just existing knowledge but also values and priorities.

We informed the respondents in the survey by briefly introducing them to the idea of immunity certificates, stating: *Suppose that there is enough evidence that suggests that people who were infected with COVID-19 and then recovered will likely be immune to re-infection for a certain period of time and are less likely to transmit the disease to others during this period. Authorities around the world may then consider policies for identifying people with immunity by using mass testing, assigning them “immunity certificates” (also called “immunity passports”) that officially declare them immune to COVID-19, and then employing them in critical positions on a voluntary basis (e.g., caring for the elderly)*. Much feedback on the questions suggested that the respondents were very well aware of the ongoing discussions on immunity certificates at the time of the survey.

## Results

### Overall attitudes towards immunity certificates

**Figure 1:**
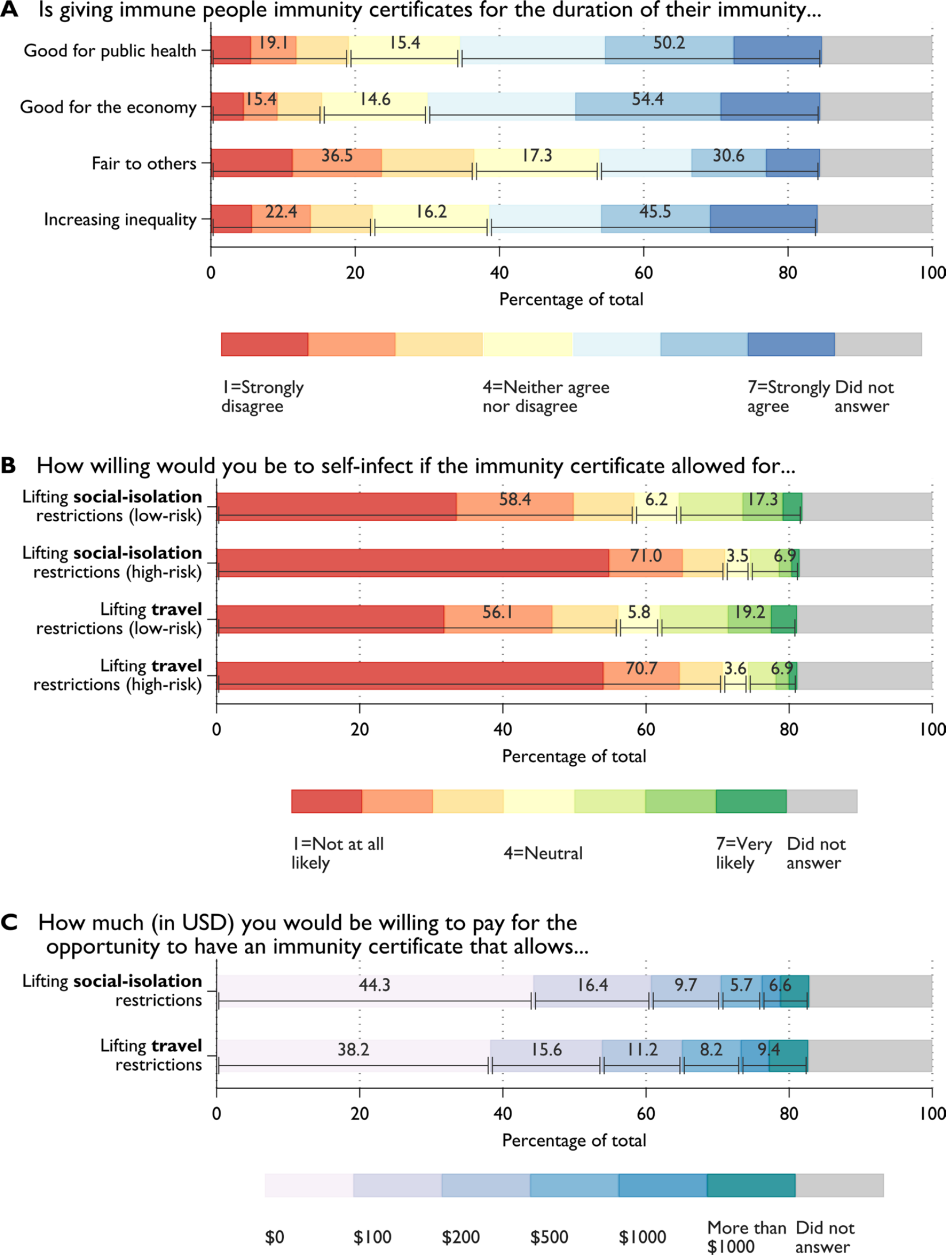
Scientists’ attitudes towards immunity certificates. *N* = 12,738 participants. (**A**) Distribution of responses to the statements “Is giving immune people immunity certificates for the duration of their immunity…” regarding (1) good for public health; (2) good for the economy; (3) fair to others who do not have immunity; and (4) increasing inequality. Share of respondents who did not answer ranges from 15.3 to 15.9%. (**B**) Willingness to self-infect for immunity certificate that lifts *social-isolation* and *travel* restrictions, if medically assessed as *low-risk* or *high-risk* groups. (**C**) Willingness to pay for immunity certificate that lifts *social-isolation* and *travel* restrictions. Share of respondents who did not answer ranges from 18.2 to 18.9%.

We asked scientists if they agree or disagree that immunity certificates are (1) *good for public health*; (2) *good for the economy*; (3) *fair to others who do not have immunity*; and (4) whether certification *increases inequality* (7-point scale from 1 (strongly disagree) to 7 (strongly agree)). The survey results are provided in Fig. 1A. About half of scientists agree that issuance of immunity certificates for the duration of immunity is good for public health (50.2%) and the economy (54.4%), while one-fifth (19.1%) and one-sixth (15.4%) disagree, respectively. In terms of fairness, about 36.5% of scholars think that issuing immunity certificates will not be fair to those who do not have immunity. 45.5% of the respondents think that immunity certificates will increase inequality in society.

To address the fear expressed by the World Health Organization that immunity certificates could increase transmission, we also confronted scholars with the following two scenarios: “Suppose that you were medically assessed to be in a *low-risk* group (*high-risk* group) and you are offered the possibility of receiving an immunity certificate for 12 months through *intentional coronavirus self-infection*. Further suppose that among every 1000 infected people in the *low-risk* group (*high-risk* group), 1 person (50 people) dies (die) due to the coronavirus. How willing would you be to self-infect if the immunity certificate allowed for (a) *lifting of social-isolation restrictions for the certificate holders*, and (b) *lifting of all restrictions and resumption of local and international travel*. Willingness to self-infect is generally low in both scenarios (Fig. 1B). Overall, in the hypothetical low-risk scenario, 56.1–58.4% of respondents indicated that they are not likely (< neutral preference) to self-infect to receive an immunity certificate. Further, about 71% of participants say they will be unlikely to self-infect if they were part of the high-risk group. It is only reasonable to expect that the probability of self-infection to obtain immunity certification is now even lower given the existence of vaccines. Vaccination is associated with substantially smaller health risks than self-infection even for low-risk groups. We also asked scientists the maximum amount (in US$) they would be willing to pay for the opportunity to have an immunity certificate {$0, $100, $200, $500, $1000, more than $1000} (Fig. 1C). Most scientists (38.2% and 44.3%) reported they would not pay to obtain an immunity certificate. It is important to note that such a direct question measures hypothetical, rather than actual, willingness to pay; thereby generating a hypothetical bias. However, applying a more indirect approach would have had the disadvantage of overloading the survey.

### Field comparisons

To explore differences across fields with respect to opinions about immunity certificates, we follow^38^ in classifying fields into five groups: *Natural Sciences* (*n* = 1710), *Applied Sciences* (*n* = 829), *Economic & Social Sciences* (*n* = 4901), *Health Sciences* (*n* = 4851), and *Arts & Humanities* (*n* = 295). The distributional differences across fields are reported in the Supplementary Information (see Supplementary Fig. 2 to 5 and Supplementary Table 1). Our results indicate that Health Scientists are slightly more skeptical about whether immunity certificates are good for public health, particularly relative to scholars in the area of Applied Sciences (Cohen’s *d* = 0.075, *p* = 0.041) and Economic & Social Sciences (*d* = 0.076, *p* = 0.006), though the absolute differences are relatively small. Economists and Social Scientists are also more in favor of immunity certificates than scholars in Arts & Humanities. The differences between Economics and Social Sciences and Health Scientists also small but visible when evaluating the economic benefit of immunity certificates (*d* = 0.06, *p* = 0.067). This does not come as a surprise, given that many of the economic advantages of immunity certificates suggested in the academic discussion were raised by Economists and Social Scientists early in the pandemic (see, e.g., 12). These scholars argue for viewing immune people as a vital resource that can be employed effectively to reduce the economic burden to society and promote the return to normality sooner, which will reduce secondary societal side effects caused by the pandemic. In the political discussion, economists have strongly emphasized the importance of putting numbers on the economic costs of shutdowns. For example, Kip Viscusi—an economist specializing in the economics of risk and uncertainty—argued in a New York Times article that “[m]aking people poorer has health consequences as well” and that “[j]obless people sometimes commit suicide, and poor people are more likely to die if they become sick, estimating that every loss of $100 million in income from the economy causes one additional death”. Health scientists are somewhat more concerned about the fairness considerations of immunity certificates (again, there are statistically significant differences between scholars from the fields of Applied Sciences (*d* = 0.093, *p* = 0.007) and Economics and Social Sciences (*d* = 0.095, *p* = 0.0002)). In an article for *The Lancet*, Alexandra Phelan—a health scientist at the Center for Global Health Science at Georgetown University Medical Center—is critical that the administration of immunity certificates would be subject to corruption and implicit biases that will then be reflected in existing socioeconomic, racial, and ethical inequities, thereby exacerbating the harm inflicted to vulnerable populations^11^. In addition, immunity certificates would risk enshrining discrimination and undermining the right to health of individuals and the population through perverse incentives. In June 2020, the Nuffield Council of Bioethics published a rapid policy briefing stating that “immunity certification raises many ethical questions concerning respect for individual rights and interests, public health responsibilities, and social justice”. For example, the report stressed that “An immune certified workforce may offer businesses a commercial or reputational advantage over competitors. Pursuing these incentives could lead to major social upheaval (as seronegative employees potentially lose opportunities to seropositive colleagues or applicants) and create coercive and stigmatising work environments”. On the other hand, in a more recent article^39^, a group of health scholars argue that the “strength of much of this opposition does not seem justified by the strength of the arguments opposing immunity passports”^8^, (p. 3), stating that “[i]mmunity passports are a potentially valuable and ethical tool” (p. 4). Interestingly, when looking at the opinions on increasing inequality, no statistically significant differences are found between fields. Overall, respondents in our sample share similar opinions regarding immunity certificates: they are generally evaluated as rather beneficial from the public health and the economic perspective. Participants have some reservations regarding fairness and worry even slightly more about inequality.

When exploring the maximum amount (in US$) scientists would be willing to pay for the opportunity to have an immunity certificate that allows a) lifting social-isolation restrictions for the certificate holders and b) lifting all restrictions and resumption of local and international travel for the certificate holders (Supplementary Fig. 6 and Supplementary Table 2), Economists and Social Scientists report a somewhat higher willingness to pay; a result that is statistically significant relative to scholars in Applied Sciences (*d* = 0.19−0.121, *p* < 0.001), Health Sciences (*d* = 0.16–0.176 *p* < 0.001), and Natural Sciences (*d* = 0.13–0.134, *p* < 0.001). On the other hand, whilst there is no statistically significant difference between willingness to self-infect to receive immunity certificates in the low-risk scenario (with the exception between Economics & Social Sciences and Health Sciences, *d* = 0.062, *p* = 0.031), some field differences emerge in the high-risk scenario (see Supplementary Fig. 7 and Supplementary Table 3). Applied Scientists (followed by Economists and Social Scientists) were more willing to self-infect to receive immunity certificates (statistically significant relative to all other groups), and Health Scientists are least likely to self-infect to obtain immunity certificates.

### Differences between US and non-US scholars

**Figure 2:**
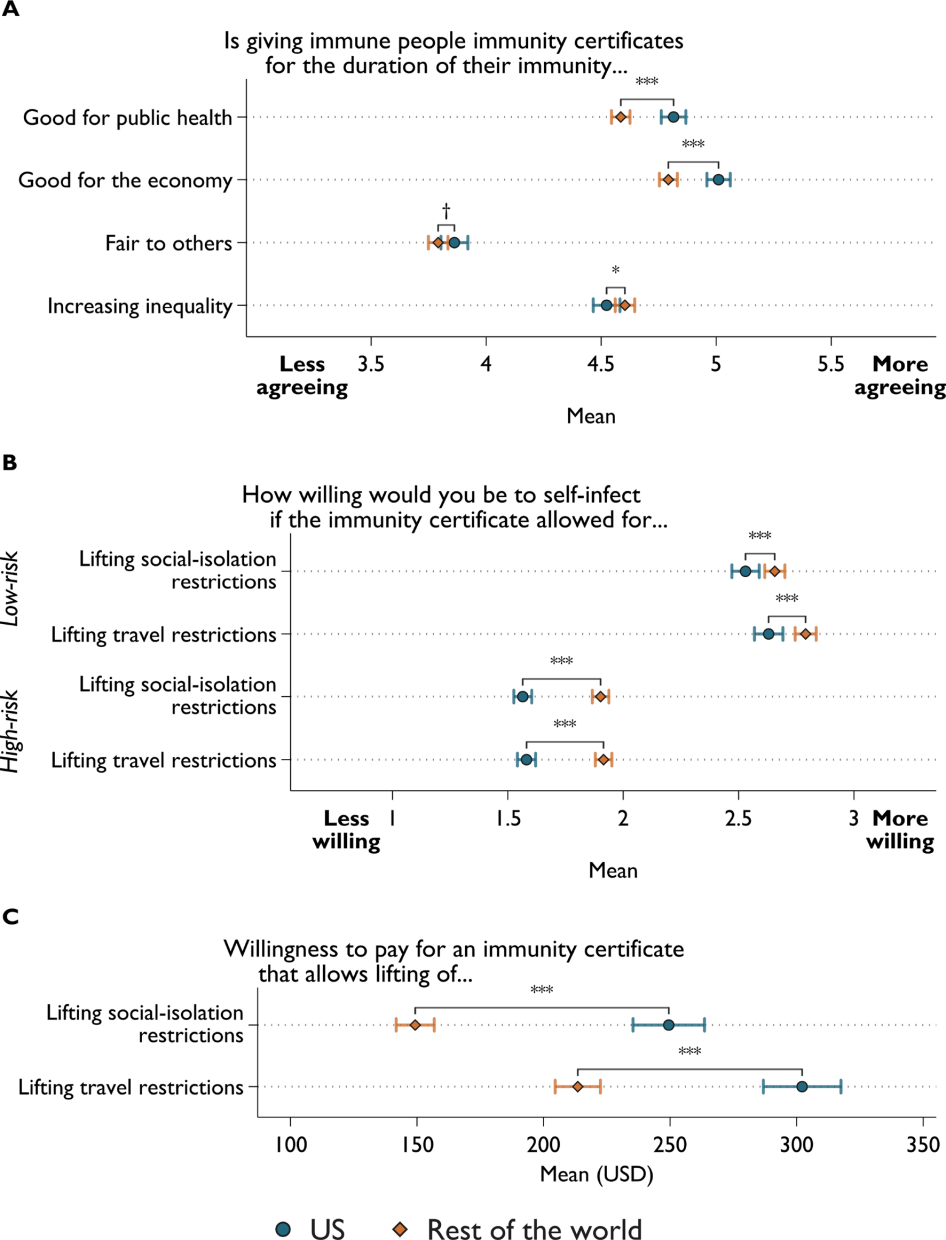
Difference in views on immunity certificates between US and non-US based scientists. (**A**) Views regarding perceived benefits to public health and economy, fairness, and societal inequality of immunity certificate. (**B**) Willingness to self-infect for immunity certificate that lifts social-isolation and travel restrictions. (**C**) Willingness to pay for immunity certificate that lifts social-isolation and travel restrictions. Two-sample mean comparison with *t-*test (two-tailed). Error bars represent 95% confidence intervals. Significance levels: ***p < .001, **p < .01, *p < .05, †*p* < .1. Results are robust to using the Wilcoxon rank sum test (Supplementary Table 4).

We take a closer look at whether we observe differences in opinions between US and Non-US scholars, and indeed, we find appreciable disparities (see Fig. 2 and Supplementary Table 4). Our data consists of a large number of US scholars (*N* = 4076, Applied Sciences: *n* = 169; Arts & Humanities: *n* = 102; Economics and Social Sciences: *n* = 1450; Health Sciences: *n* = 1937; Natural Sciences: *n* = 418). US scholars are significantly more in favor of immunity certificates when considering their relevance for public health (*d* = − 0.117, *p* < 0.001) and the economy (*d* = − 0.113, *p* < 0.001) (Fig. 2A). In general, Non-US scientists regard immunity certificates as more unfair to others (*d* = − 0.037, *p* < 0.001) and more likely to increase inequality (*d* = 0.053, *p* < 0.001), relative to their US-based counterparts; however, these effect sizes are small. Nevertheless, US scientists are less willing to self-infect when in a low-risk (*d* = 0.048–0.07, *p* < 0.01) and high-risk group (*d* = 0.195–0.189, *p* < 0.001), regardless of the type of restrictions the immunity certificate could lift (Fig. 2B). Regarding willingness to pay, US scholars show a higher willingness to pay for immunity certificates, question a) *M* = 249.49 (*SD* = 426.37) for US versus *M* = 149.33 (*SD* = 322.27) for Non-US (*d* = − 0.25, *p* < 0.001); question b) *M* = 302.18 (*SD* = 462.38) for US versus *M* = 213.53 (*SD* = 382.43) for Non-US (*d* = − 0.188, *p* < 0.001) (Fig. 2C).

Comparing US and Non-US scholars within fields (Supplementary Fig. 8 to 10 and Supplementary Table 5), both the US Health Sciences scholars and the US Economists and Social Scientists are more positive towards immunity certificates as something that is good for public health (*d* = − 0.193, *p* < 0.001 and *d* = − 0.069, *p* = 0.0278) and good for the economy (*d* = − 0.198, *p* < 0.001 and *d* = − 0.068, *p* = 0.029) (Supplementary Fig. 8). US Health Scientists have fewer fairness concerns with respect to immunity certificates (*d* = − 0.083, *p* = 0.007) and are less likely to view immunity certificates as increasing inequality (*d* = 0.090, *p* = 0.004). US and non-US differences in willingness to pay for an immunity certificate are evident in almost all fields, with the largest discrepancy among Natural Scientists (*d* = − 0.320–− 0.343, *p* < 0.001) (Supplementary Fig. 9). In terms of willingness to acquire an immunity certificate through self-infection, US Health Scientists hold stronger opposing views compared to their non-US counterparts in all four scenarios (low- or high-risk groups and whether immunity certificate would mean lifting social-isolation and travel restrictions; *d* = − 0.075–− 0.206, all *p* < 0.05). Nevertheless, while US scientists in other fields are also less willing to self-infect to receive an immunity passport (relative to non-US scientists in the same field) when they were in the hypothetical high-risk group, said differences were less visible in the low-risk scenario (Supplementary Fig. 10).

### Consensus

**Figure 3:**
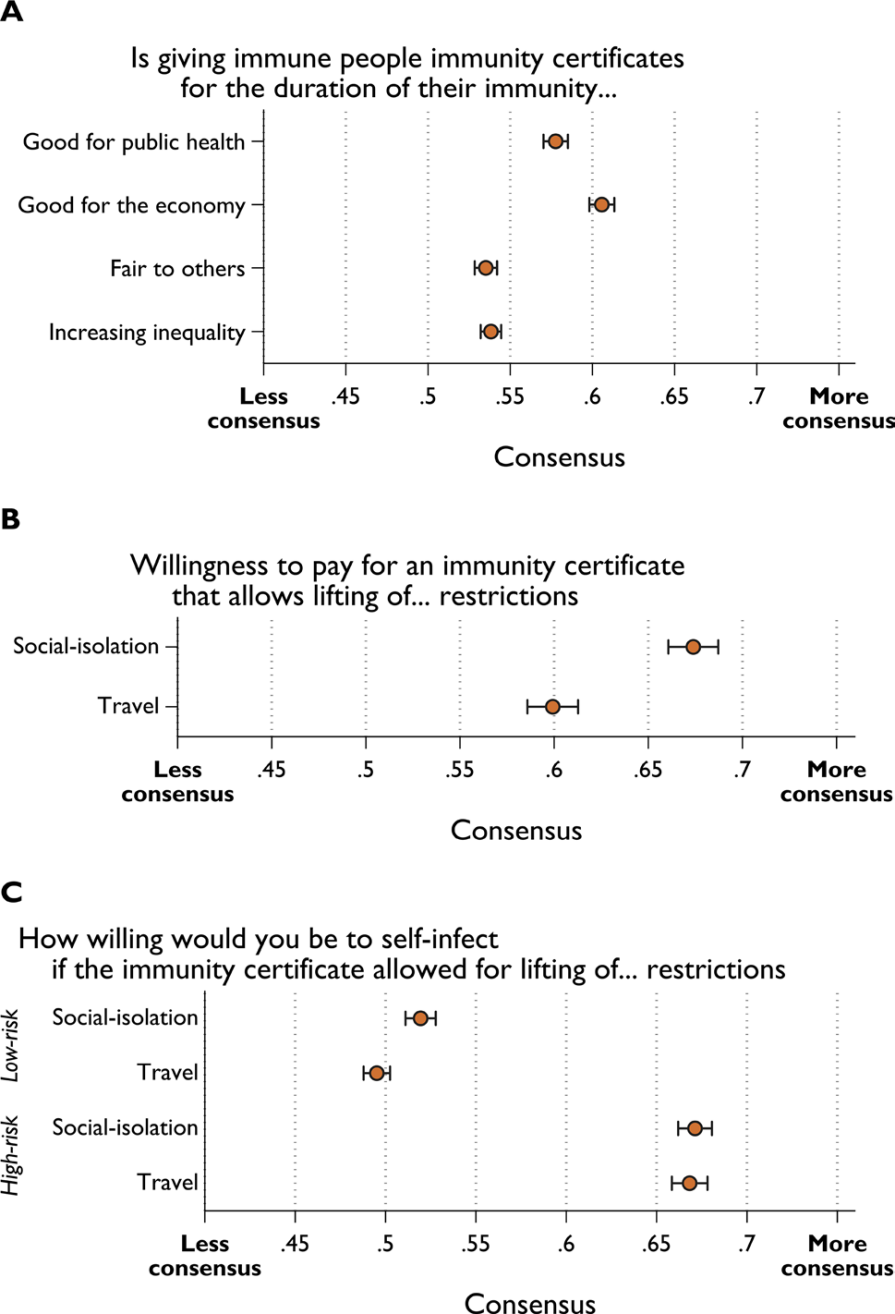
Consensus among scientists. The entropy-based consensus measure takes the value of 1 when all responses are concentrated on one option and value of 0 when responses are evenly distributed in each available option. (**A**) Views on perceived benefits to public health and economy, fairness, and societal inequality of immunity certificates. (**B**) Willingness to pay for an immunity certificate that lifts social-isolation and travel restrictions. (**C**) Willingness to self-infect for an immunity certificate that lifts social-isolation and travel restrictions. Error bars represent 95% confidence intervals obtained from bootstrap resampling with 300 replications. Null responses are excluded from the calculation of consensus.

Employing an entropy-based consensus measure, we observe that scholars show a higher level of consensus on the questions around favoring immunity certificates for public health (C = 0.578) and the economy (C = 0.606) compared with the questions around fairness (C = 0.535) and inequality (C = 0.538) (Fig. 3A). The latter questions are discussed from an ethical perspective in both academic and non-academic channels which may explain lower levels of consensus. Looking at the consensus *within* each field (Supplementary Fig. 11), Economics and Social Sciences and Health Sciences tend to report higher levels of consensus. However, when closely examining whether field differences are statistically significant with Bonferroni adjustments, only the fairness and inequality questions lead to statistically significant field differences (Supplementary Table 6). Natural Scientists indicate the lowest levels of consensus within their field and the differences are statistically significant in comparison to Health Scientists (*p* = 0.090) for fairness; and Health Sciences (*p* < 0.001), and Economics & Social Sciences (*p* = 0.063) for inequality.

Overall, consensus on the willingness to pay (C = 0.600–0.674) for an immunity passport is quite high (Fig. 3B). Economists report the lowest levels of consensus on the willingness to pay for an immunity passport (significantly lower than Health Sciences, Natural Sciences, and Applied Sciences) (Supplementary Fig. 12A and Supplementary Table 7). The lowest level of consensus among all questions is on the issue of self-infection with the low-risk scenario (Fig. 3C; C = 0.495–0.519); there are no significant differences between fields on the low-risk scenario (Supplementary Fig. 12B and Supplementary Table 7). On the other hand, consensus is high for the high-risk scenario (C = 0.668–0.671), with Health Scientists reporting a significantly higher level of consensus than do Economists and Social Scientists (*p* = 0.013 and *p* = 0.0057), Natural Scientists (*p* = 0.027 for lifting social-isolation restrictions) or Applied Scientists (*p* = 0.0021 and *p* = 0.0022).

US scholars demonstrate higher levels of consensus than non-US scholars across all questions (Supplementary Fig. 13 and Supplementary Table 8), except for those regarding willingness to pay, where consensus is lower for US scientists. The differences are statistically significant for all the questions. In the comparison of US and non-US scholars within fields (Supplementary Fig. 14 to 15 and Supplementary Table 9), again, US scholars tend to show a higher level of consensus, except for willingness-to-pay questions. However, key differences are found within Economics and Social Science and Health Sciences on scientists’ perceptions that immunity passports are good for public health (*p* = 0.0072 and *p* < 0.001, respectively) and good for the economy (*p* = 0.014 and *p* < 0.001, respectively), and among Health Sciences with respect to fairness to others (*p* = 0.048). Some field differences are also found for willingness to pay, and self-infection in the high-risk scenario (statistically significant differences for Economics and Social Sciences, Health Sciences, and Natural Sciences). Consensus is also higher among US Health Scientists (relative to Health Scientists elsewhere) in relation to willingness to self-infect in the low-risk scenario.

### Political views

**Figure 4:**
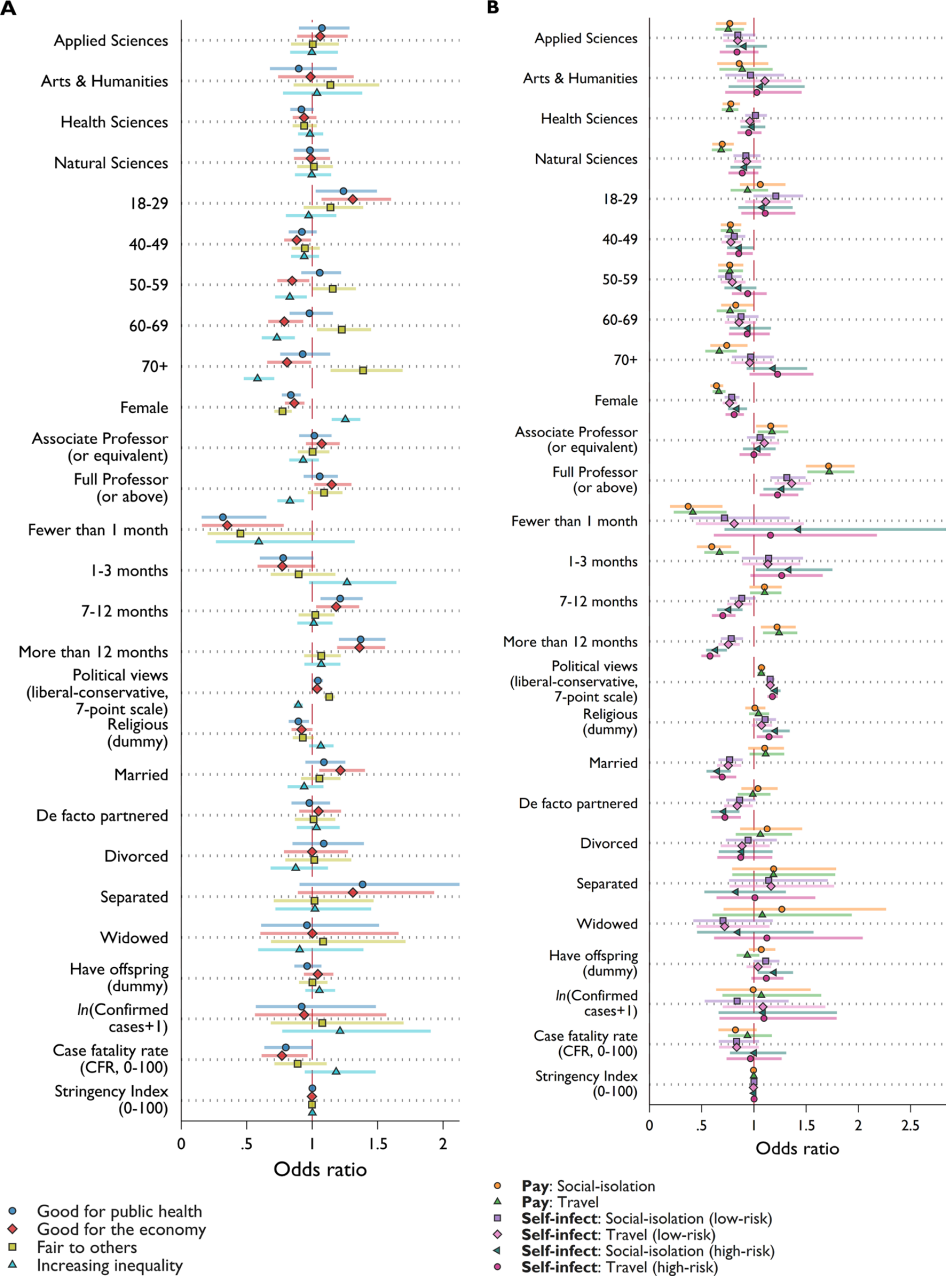
Ordered logit regressions. Presented are odd ratios of covariates from 10 ordered logit regressions for (**A**) perceived benefits to *public health* and the *economy*, and *fairness* and *inequality* concerns and (**B**) willingness to pay and willingness to self-infect for immunity certificate. Error bars represent 95% confidence intervals. Full regression results are presented in Supplementary Table 10 for (**A**) and Supplementary Table 11 for (**B**).

We explore the relevance of political views in greater detail (Fig. 4 and Supplementary Table 10 and 11) by running a set of ordered logit regressions. Scientists who hold more conservative views (more right-wing) are significantly more in favor of immunity certificates. They evaluate them as good for public health (*Odds Ratios* 1.044, *p* = 0.023), good for the economy (*OR* 1.038, *p* = 0.052), fair to others (*OR* 1.130, *p* < 0.001) and do not see them as potentially increasing inequality (*OR* 0.893, *p* < 0.001). Their willingness to pay is higher (*OR* 1.071–1.073, *p* < 0.001), as is their willingness to self-infect to receive immunity certificates (Low-risk scenario: *OR* 1.156, *p* < 0.001; High- risk scenario: *OR* 1.178–1.206, *p* < 0.001). This holds while accounting for individual characteristics, confirmed COVID-19 cases, case fatality rate, stringency index and different fixed effects.^9^ classify and reject arguments for immunity certificates as belonging to a conservative political view by highlighting the relevance of liberal individualism. They frame their own political rejection of immunity certificates as part of a communitarian approach to public health in line with progressive views.^40^ responded by stressing that “liberties should be restored to immune individuals precisely because they are not anymore a threat to the greater good”.

### Individual differences

The results from the ordered logit regressions revealed individual differences in scientists’ attitudes toward immunity certification. Clear patterns are observed throughout all questions. Women scholars are significantly less likely to favor immunity certificates (estimates are statistically significant at 0.1% level for all ten dependent variables) even when accounting for other characteristics, political views, and contextual factors, such as case and fatality rates as well as the stringency implementations. Interestingly, a recent study also indicates that women are more likely to perceive COVID-19 as a very serious health issue and more likely to favor using restrictive public policy measures, a difference that is considerable in all eight OECD countries explored^41^. Controlling for academic rank, younger scholars (age group 18–29) tend to be more supportive of immunity certificates in terms of their benefit for public health (*OR* 1.24, *p* = 0.026) and the economy (*OR* 1.31, *p* = 0.009) compared to the baseline age group^30–39^, while scientists above 40 showed less agreement regarding the economic benefit (*p* < 0.001 for all age groups above 40). Scientists in the age group > 50 demonstrated substantially more support for immunity passports with respect to fairness and inequality (relative to the age group 30–39, *p* < 0.001 for all age groups above 50). Nevertheless, scientists with full professorship showed more support (compared to assistant professor or below) in terms of economic benefit (*OR* 1.149, *p* = 0.026) and inequality concerns (*OR* 0.83, *p* = 0.003). More senior ranked academics (full professorship) also expressed higher willingness to pay (*OR* 1.716–1.723, *p* < 0.001) and self-infect for immunity certificates (*OR* 1.268–1.362, *p* < 0.001), while older age groups are less willing to pay for immunity certificates. Age groups 40–49 and 50–59 report a weak inclination to self-infect in both scenarios. One should note that the correlation between academic rank and age is high (0.605, *p* < 0.001); in an unreported analysis without controlling for academic rank, scientists in the older age group (60+) expressed more favorable views towards immunity certificates (the estimated odds ratios are above 1 for the fairness, willingness to pay, and willingness to self-infect questions and less than 1 for inequality concerns).

Married scientists (relative to those who are not in a relationship) are more supportive of the concept of immunity certificates for its perceived benefit for the economy (*OR* 1.216, *p* = 0.007) but less willing to self-infect to receive one (*p* < 0.001; this also holds for those in a de facto partnered relationship). Non-religious scientists (who never attend religious services) are more supportive of immunity certificates due to the potential benefit but are less inclined to self-infect. There is a tendency for those who believe it will take longer to get back to normality to answer in favor of immunity certificates for promoting public health and the economy. Scientists who imagine better prospects of returning to normality sooner have a lower willingness to pay but a higher willingness to self-infect to obtain an immunity certificate. Further analyses on the difference in prospect of returning to normal are given in *Appendix SI* (see ‘*Return-to-normality timeline’*).

## Discussion

This study is the first to explore scientists’ attitudes and opinions regarding immunity certification. As an innovative contribution, we present results based on our large-scale survey conducted among 12,738 scientists from 37 subfields and 63 countries, with 4076 respondents from the US alone. Immunity certificates were an early topic of contention in this pandemic, but may serve as a policy tool in the future. Vaccination certificates are commonly used for vaccine induced immunity. During early phases of pandemics when no vaccines are available, immunity certificates might be a policy tool to reduce the economic and societal burden of the pandemic. From a perspective of individual freedom, it is difficult to justify severe restrictions for convalescents.

As our survey on scientists’ perceptions of immunity certificates was conducted when vaccinations were not yet available, our results can be interpreted as a conservative threshold on the acceptance of vaccination-induced immunity and corresponding certification. Several arguments put forward by scholars against immunity certificates for convalescents are also applicable to vaccine-induced immunity; thus, the same scholars may object to any reduction of restrictions for vaccinated individuals until herd immunity is achieved^40^, even though models have failed to support achieving herd immunity as a practical objective^42^. Due to the uniform and controlled nature of treatment, the response of a vaccine-induced immunity is more predictable.

Our results indicate that Scientists tend to have favorable opinions on immunity certificates but raise some doubts regarding fairness and inequality. Comparing different fields suggests that Health Scientists are slightly less in favor of immunity certification, while Economists and Social Scientists tend to be supportive. US scientists tend to be more in favor of immunity passports even after controlling for many factors including contextual aspects such as the number of COVID-19 cases or deaths. A similar result is also found for Health Scientists.

There is less consensus among scientists on the questions of fairness and inequality. Scholars from fields that are more active in the policy discussion process report higher levels of consensus; in particular, Health Scientists report high consensus values in relative terms. Self-infection in a low-risk scenario produced less agreement compared with other questions. In addition, US scholars report a higher level of consensus than non-US scholars. Political views matter for support of immunity certificates—with scientists holding conservative views being more in favor. When exploring individual differences, we found that women are less supportive of immunity certificates. Young people also tend to care more about the immunity value in terms of economic benefit.

While many scholars are concerned about the effects of immunity certification on fairness and inequality, there will be a lag in vaccine-induced protection for countries that are particularly vulnerable to prolonged lockdowns but in which many convalescents might be found due to their comparatively young populations. The perceived benefits of immunity passports are likely to increase the longer it takes to bring back normality. Besides understanding the opinions of single scholars who hold the microphone in scientific outlets, it is worth mapping—as we have done—the opinions of a large number of scholars from different fields, and analyzing how individual differences shape the scholars’ opinions. Such mapping contributes to the important debate regarding which policy responses society should follow when coping with pandemics in the future.

## Methods

### Data

We conducted an online survey via the SurveyMonkey platform with scholars who have served as the corresponding author of an article published in the top-ranked journals in 55 scientific fields in the last five years (from 2015 to beginning of 2020). Journals were selected based on their 2019 SCImago Journal Ranking (SJR) in the 55 subject categories from 13 scientific areas defined by Scopus (*Arts and Humanities; Business, Management and Accounting; Economics, Econometrics and Finance; Energy; Health Professions; Immunology and Microbiology; Medicine; Multidisciplinary; Neuroscience; Nursing; Pharmacology, Toxicology and Pharmaceutics; Psychology; Social Sciences*). A total of 849 journals were sampled (see Dataset S1 in *Appendix SI*). We extracted the e-mail address of the corresponding authors from the journal publication records downloaded from the Scopus citation database when available (a total of 318,251 e-mail addresses were extracted). As numerous social scientists and economists have highlighted the costs and trade-offs of the pandemic for society and the economy^37^, we think it is important to oversample social scientists. For example, based on the COVID-19 publication records dataset from Dimensions (as of 5 June 2021), economics and social sciences publications account for 47.5% of all non-health sciences COVID-19-related studies. Therefore, we also included scholars registered in the bibliographic database RePEc (https://econpapers.repec.org/RAS/) in our sample pool (addition of 68,470 e-mail addresses); this provides a total of 353,583 unique e-mail addresses for scientists. From the sample pool, we randomly selected two-thirds and sent out the survey invitations from May 4 to 17, 2020, except on Sundays. The number of survey invitations sent on each day were evenly distributed. A reminder e-mail was sent to invited participants who had not opened the survey link two weeks after the initial e-mail invitation. The survey was closed on June 3, 2020.

Of the 220,923 invitations sent (22,074 (9%) email addresses were invalid), 41.35% (91,346) were opened. The response rate based on e-mails opened was 13.93% (5.76% based on valid invitation), with 98% completed within 24 h upon opening the invitation link. The median response time was 16 min (M = 0.12 days, SD = 1.25). Our response rate is comparable to other studies with surveys sent to scientists through e-mail lists obtained from academic databases such as Scopus. For example^43^ reported a 14.1% response rate from the online survey on public communication with 100,000+ faculty members from 73 land-grant universities^44^; recorded a response rate of 12% in a web survey study exploring the consensus among scientists on the highly debated topic of climate change and environmental policy, using an email list composed from Scopus^45^; reported a response rate of 10.3% with a survey distributed to 729 authors via email and social networking sites^46^; investigated the opinion of scientists on the peer review process from a list of academics at universities ranked highly at the Times Higher Education (THE) university rankings and obtained a response rate of about 5%. We also acknowledge the variation in the response rates across fields (see Supplementary Table 16), ranging from 8.19% (*Health Professions*) to 19.47% (*Economics*). While our sample is large, self-selection remains one of the significant challenges in survey studies; for example, scholars in health science might be less inclined to respond due to increased workload during COVID-19.

The survey consisted of several sections on topics related to COVID-19, including a section devoted to opinions about immunity certificates. Questions pertaining to basic demographics were asked at the start of the survey. In addition, we asked our participants a series of control questions, including their political views, religiosity, and marital status at the end of the survey. Participation in the survey was completely voluntary and subjects were able to skip any question they did not want to answer or quit the survey at any time. For this reason, there is a larger proportion of missing values on the control variables at the end of the survey (about 33%). To assess the reliability of the regression analysis (sensitivity to missing data), we report the results without these control variables (see Supplementary Information). Participants were self-selected from the pool of scientists mentioned above. Participants were told that the survey pertained to the COVID-19 health crisis, but they were not aware of the specific contents of each section before taking part in it. In order to incentivize the interest in the survey, the subjects were offered a $500 lottery for themselves and $500 for a charity of their choice upon leaving a contact email address in case they won the prize. Ethical clearance for the data collection was granted on April 23 by the Ethics Commission of the Frankfurt School of Finance and Management. All participants provided written informed consent at the outset of the survey, and all methods were performed in accordance with the relevant guidelines and regulations. Preliminary analysis on the difference in responses between health scientists and non-health scientists is provided in^47^.

### Sample description

Descriptive statistics of the sample are presented in Supplementary Table 17. We recorded a total of 12,738 responses from scientists across different disciplines. Females represent around 42% (*n* = 5335) of the sample, while 57% (*n* = 7218) of the participants are male. Most participants were in the age brackets 30 to 39 years old (32%, *n* = 4131) and 40 to 49 years old (29%, *n* = 3637). In addition, we recorded information of unique relevance to this demographic group, such as their field of study, how many of them have completed a PhD and whether they hold a professorship (28.5%, *n* = 3631). Most of the respondents are from Europe (42.3% *n* = 5408) and North America (37.22% *n* = 4759). The majority of participants held an assistant professorship (equivalent or below) (52.8% *n* = 6664).

Compared to economics scholars recruited from the top journals in the fields (*n* = 1440), the pool of survey participants drawn from the *RePEc* register (*n* = 1019) is composed of more males (5.3 percentage points, *p* = 0.004) and are from older age groups (*p* = 0.044 based on a two-tailed rank sum test). However, we did not find any significant discernible difference in other sample characteristics (e.g., professorship, political views, religiosity, marital status) between the two samples (all *p* > 0.1). Nevertheless, we control for this by including a dummy variable for participants from the *RePEc* sample in the regression analyses. While the sample from *RePEc* was more supportive of the immunity certificates in terms of inequality concerns (Supplementary Table 12) and have higher willingness to pay for the immunity certificates (Supplementary Table 13) than other economists and social scientists, removing the *RePEc* sample from the analysis does not change our qualitative and quantitative findings reported in the main text. The main results excluding the *RePEc* sample are reproduced in the *Appendix SI* (Supplementary Fig. 18 to 21 and Supplementary Table 18).

#### COVID-19 data

To control for contextual factors due to development of the COVID-19 pandemic, we collected the daily confirmed case and case fatality rate (CFR) statistics at the country level, as well as a measure designed to capture the stringency level of government policy responses. COVID-19 statistics were obtained from the Center for Systems Science and Engineering (CSSE) at Johns Hopkins University^48^ and the Stringency Index were obtained from the Oxford COVID-19 Government Response Tracker (OxCGRT))^49^. We add 1 to the daily number of COVID-19 confirmed case variable and implement a log transformation. The Stringency Index is the sum of eight containment and closure policy indicators together with the presence of public information campaigns (see^49^ for details). The three cross-country daily measures were merged with the self-reported country of residency and survey submission date variables. These variables were included as controls in the regression models, together with country and time fixed effects.

### Empirical approach

Appropriate statistical tests were chosen to perform the response comparisons between groups; both parametric and non-parametric tests were employed. Due to the ordinal nature of the response variables, we perform a non-parametric pairwise multiple comparison^50^ and adjust the false discovery rate using the Benjamini–Hochberg stepwise adjustments. We also report the results of the Kruskal–Wallis rank test of the hypothesis that responses from different fields are from the same population. We employed both mean comparison *t*-test and the non-parametric Wilcoxon rank-sum for comparison between US and non-US responses. For US and non-US difference within fields, we implemented the Bonferroni correction to account for multiple comparison by inflating the significance cut-off by five-fold. To calculate the effect size for these comparisons, we follow the transformation of Cohen’s *d* for ordinal data proposed by^51^, where *d* ${\text{ = ~2*z/}}\sqrt n$. Exact *p*-values (two-tailed) are reported. Statistical analyses were performed using Stata MP 16.1.

### Consensus

To examine the degree of agreement among scientists, we closely follow the approach of^52,53^ when measuring consensus from variables with an ordinal scale. Consensus of the ordinal response variable *X* with *i* categories is defined as: ${\text{Consensus}}\left( X \right) = 1 + \mathop \sum \limits_{{i = 1}}^{n} p_{i} \log _{2} \left( {1 - \frac{{\left| {X_{i} - \mu _{X} } \right|}}{{max_{X} - min_{X} }}} \right)$, where *p *is the share of responses (excluding non-responses). A value of 0 indicates the participants’ responses are evenly split to the two extremes, while a value of 1 means that all responses are in the same category. The consensus score is around 0.45 (depending on the number of response categories) if responses are evenly split into each category. 95% confidence intervals (error bars) of the consensus measure are constructed by performing bootstrap resampling with 300 replications. We employ the two-sample *t*-test to test for statistically significant differences in the consensus scores between groups (across fields or US to non-US). Bonferroni adjustments were also used for multiple-field comparisons. Computing consensus using the Shannon Entropy equation, i.e., $1 - \frac{{\sum p_{i} \times \ln p_{i} }}{{n \times 1/n \times \ln \left( {1/n} \right)}}$, yields identical qualitative conclusions.

### Ordered logit regressions

We employed the ordered logit regression model to examine the effect of sample characteristics and other factors on the response outcome. The ordered logit model is a more suitable model than the commonly used ordinary least squared (OLS) model as it recognizes that the response data is ordinal rather than interval. The ordered logit coefficient indicates the expected increase in the log odds of being in a higher level of the response variable, given a 1-unit increase in the predictor variable, holding other variables in the model constant. For ease of interpretation, we report the estimated proportional odds ratios (by exponentiating the coefficients), which can be interpreted as the odds for being in a higher level of the response variable (i.e., proportional odds times larger).

## Supplementary Information

Supplementary Information.

## Data Availability

All data analyzed in the current study are made available from Open Science Framework (https://osf.io/xghq7/). Data on selected journals are provided in the Supplementary Information.

## Code availability

All computer code used in the data analysis is available from the corresponding author upon reasonable request.
