# Supplementary material for: Scientists have favorable opinions on immunity certificates but raise concerns regarding fairness and inequality
Source: Sci Rep. 2021 Jul 7;11:14016. doi: 10.1038/s41598-021-93148-1 (PMC8263576; doi:10.1038/s41598-021-93148-1)
Supplement: Supplementary file 1 — Supplementary Information. [file 41598_2021_93148_MOESM1_ESM.docx]

**Supplementary Information to:**

**“Scientists have favorable opinions on immunity certificates but raise concerns regarding fairness and inequality”**

Iván Aranzales, Ho Fai Chan, Reiner Eichenberger, Rainer Hegselmann, David Stadelmann, Benno Torgler

[Geographical distribution of survey participants 2](#_Toc74058463)

[Field difference 3](#_Toc74058464)

[US and non-US difference 10](#_Toc74058465)

[Consensus 16](#_Toc74058466)

[Regressions-based results 27](#_Toc74058467)

[Return-to-normality timeline 35](#_Toc74058468)

[Response rate 39](#_Toc74058469)

[Sample characteristics 40](#_Toc74058470)

[Replication of main results excluding the *RePEc* sample 42](#_Toc74058471)

# Geographical distribution of survey participants


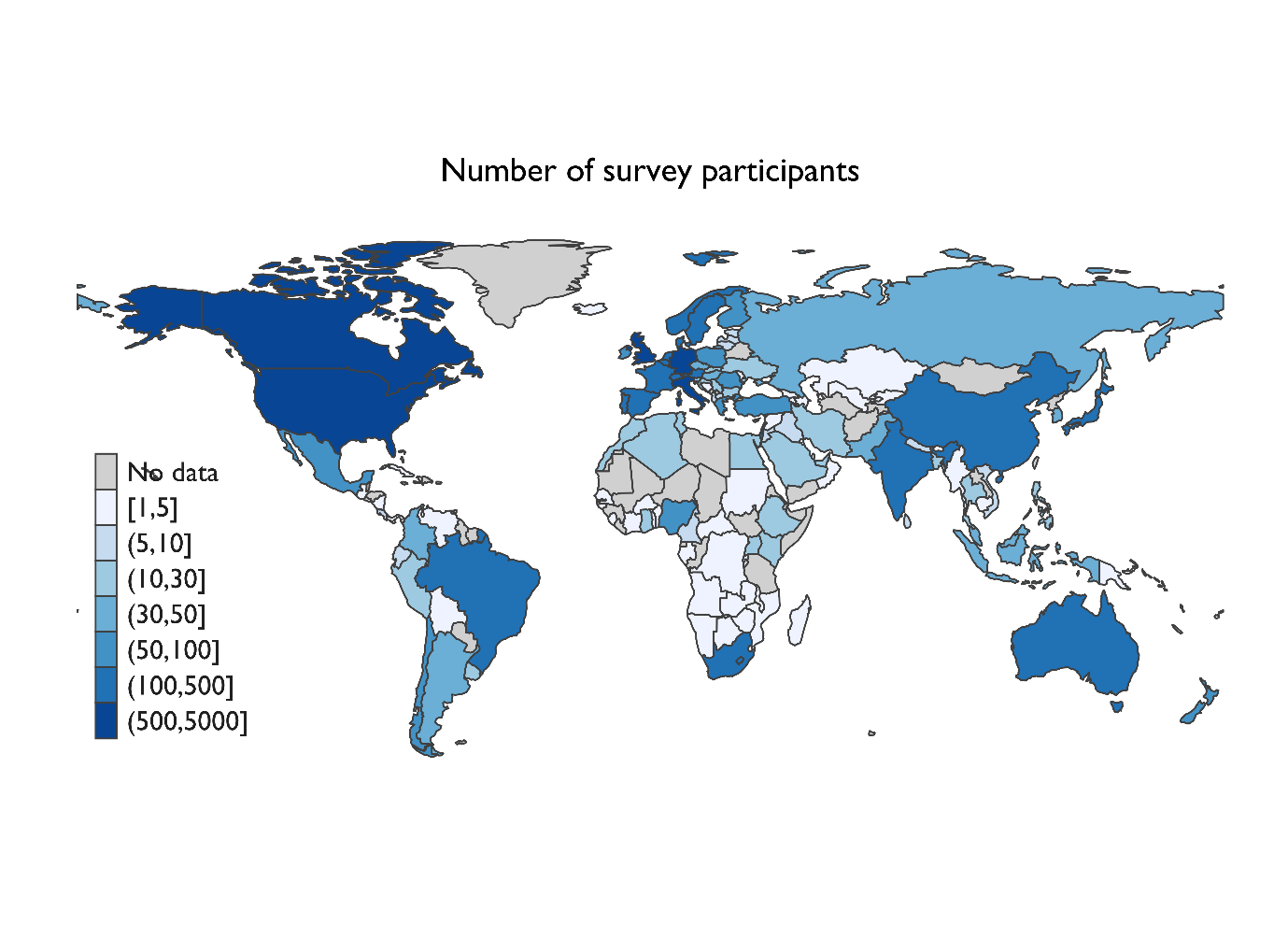


**Supplementary Figure 1. Survey participants by country.** Responses were received from 63 countries, with 44 countries returning at least 30 responses. The figure was created with STATA 16.1 MP (StataCorp. 2019. Stata Statistical Software: Release 16. College Station, TX: StataCorp LLC. <https://www.stata.com/>).

# Field difference

**
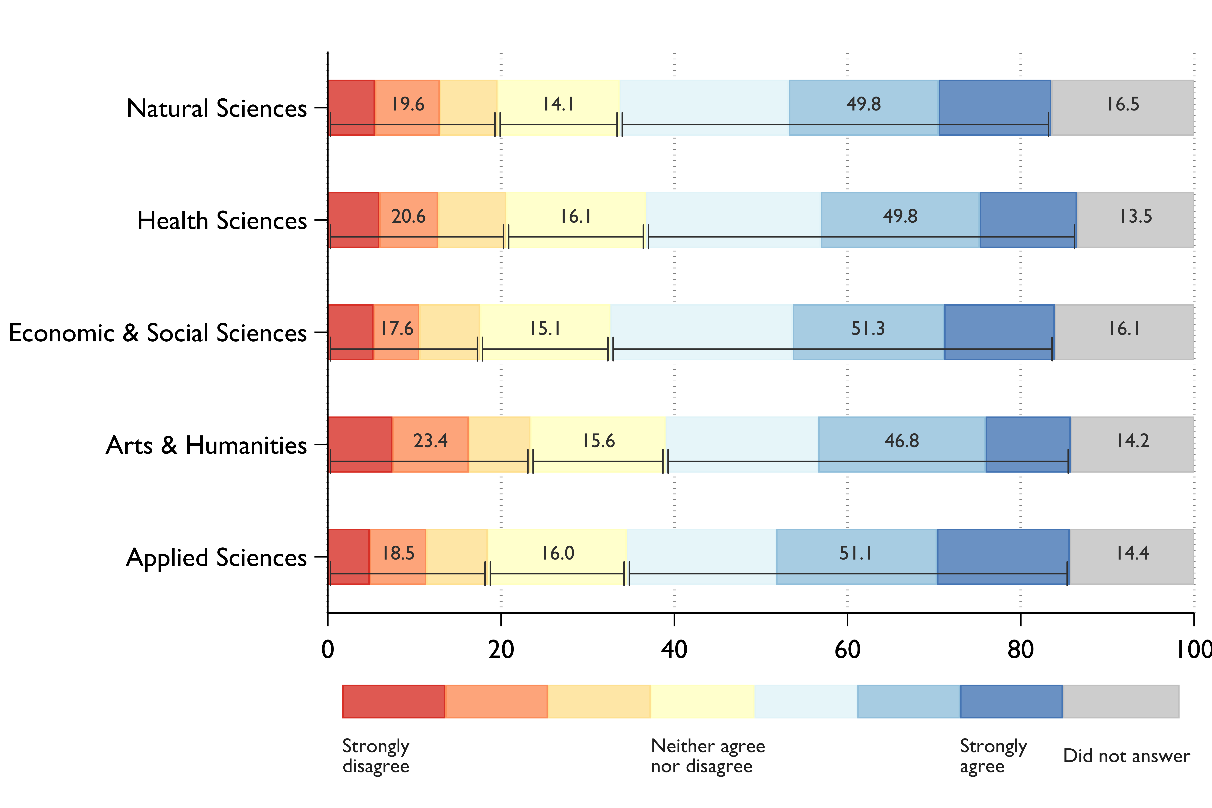
**

**Supplementary Figure 2. Opinion on whether immunity certificates are *good for* *public health*, by field.** *N* = 10,789 non-missing responses. Kruskal-Wallis equality-of-populations rank test (tie-corrected *χ^2^*(4) = 18.11; *p* = .00117).


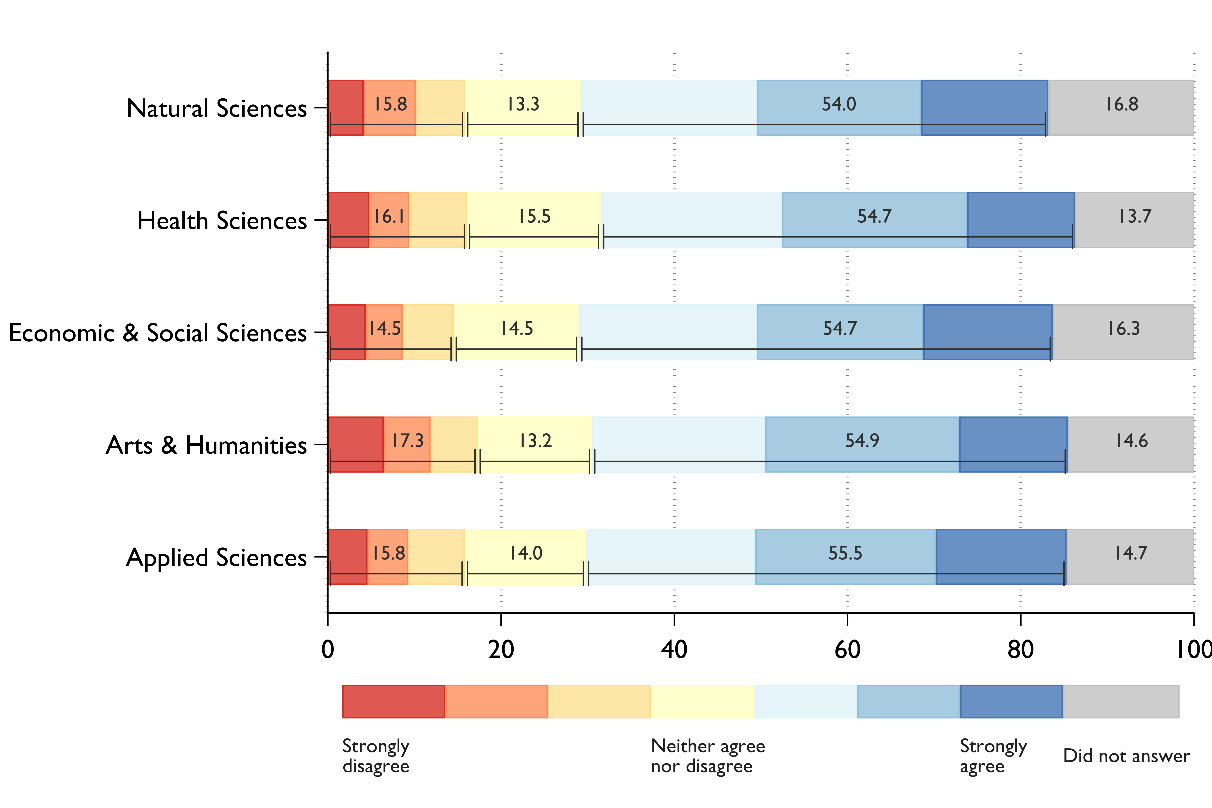


**Supplementary Figure 3. Opinion on whether immunity certificates are *good for the economy*, by field.** *N* = 10,758 non-missing responses. Kruskal-Wallis equality-of-populations rank test (tie-corrected *χ^2^*(4) = 8.271; *p* = .0821).


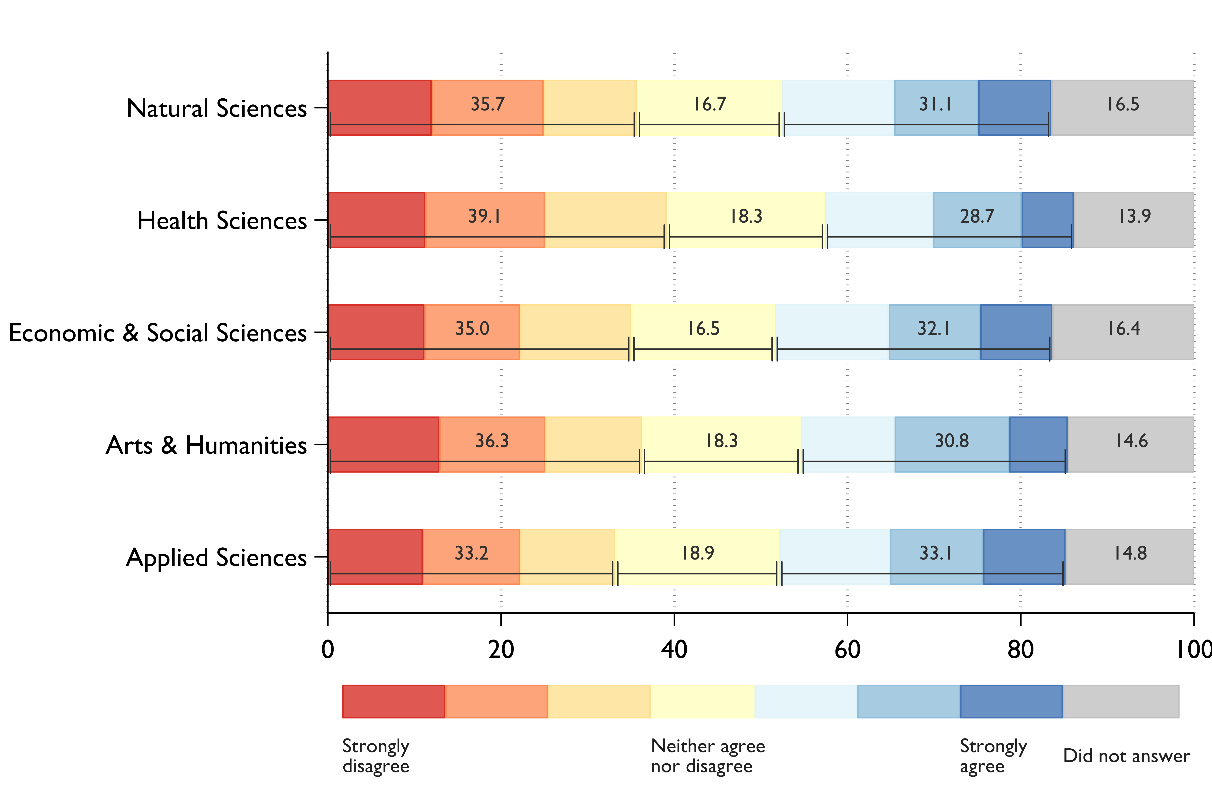


**Supplementary Figure 4. Opinion on whether immunity certificates are *fair to others who do not have immunity*, by field.** *N* = 10,754 non-missing responses. Kruskal-Wallis equality-of-populations rank test (tie-corrected *χ^2^*(4) = 22.89; *p* = .000133).

**
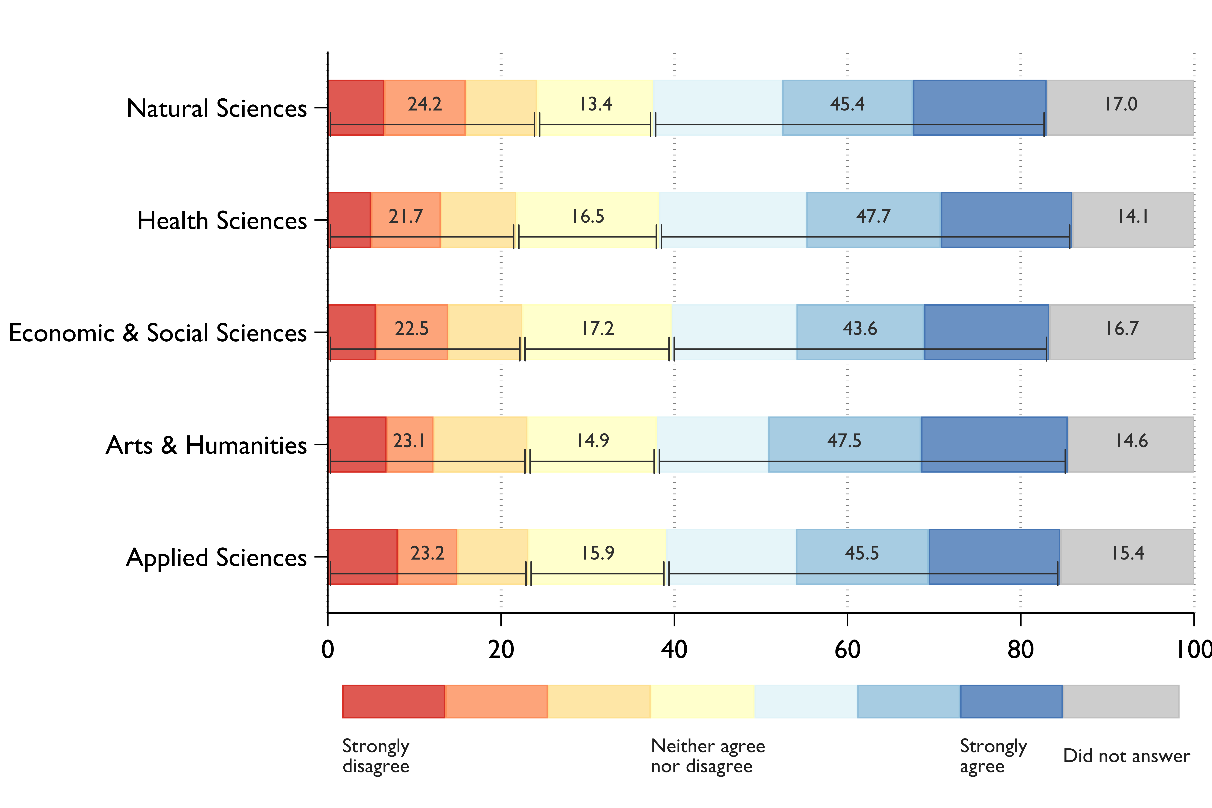
**

**Supplementary Figure 5. Opinion on whether immunity certificates would *increase inequality*, by field.** *N* = 10,712 non-missing responses. Kruskal-Wallis equality-of-populations rank test (tie-corrected *χ^2^*(4) = 4.972; *p* = .29).

**Supplementary Table 1. Differences in opinion towards COVID-19 immunity certificates across fields.**

|  |  |  | *Good for public health* | | | *Good for the economy* | | | *Fair to others* | | | *Increase inequality* | | |
| --- | --- | --- | --- | --- | --- | --- | --- | --- | --- | --- | --- | --- | --- | --- |
|  |  |  | *d* | z-stat. | *p*-val. | *d* | z-stat. | *p*-val. | *d* | z-stat. | *p*-val. | *d* | z-stat. | *p*-val. |
| Applied Sciences | vs. | Arts & Humanities | .151 | 2.365* | .030 | .050 | .767 | .443 | .070 | 1.101 | .226 | -.060 | -.955 | .425 |
| Applied Sciences | vs. | Economic &  Social Sciences | .023 | .771 | .220 | .001 | .029 | .488 | .026 | .894 | .265 | .004 | .149 | .490 |
| Arts & Humanities | vs. | Economic &  Social Sciences | -.066 | -2.189* | .036 | -.026 | -.848 | .495 | -.020 | -.684 | .275 | .035 | 1.174 | .601 |
| Applied Sciences | vs. | Health Sciences | .075 | 2.640* | .021 | .043 | 1.494 | .338 | .093 | 3.206** | .003 | -.026 | -.919 | .298 |
| Arts & Humanities | vs. | Health Sciences | -.031 | -1.02 | .192 | .002 | .069 | .525 | .023 | .766 | .277 | .016 | .502 | .440 |
| Economic &  Social Sciences | vs. | Health Sciences | .076 | 3.454** | .003 | .06 | 2.711* | .034 | .095 | 4.278*** | <.001 | -.044 | -1.981 | .238 |
| Applied Sciences | vs. | Natural Sciences | .055 | 1.276 | .144 | .019 | .446 | .410 | .068 | 1.616 | .133 | -.006 | -.125 | .450 |
| Arts & Humanities | vs. | Natural Sciences | -.081 | -1.679† | .093 | -.026 | -.523 | .429 | -.005 | -.094 | .463 | .046 | .941 | .347 |
| Economic &  Social Sciences | vs. | Natural Sciences | .023 | .888 | .208 | .017 | .628 | .442 | .032 | 1.234 | .217 | -.009 | -.385 | .437 |
| Health Sciences | vs. | Natural Sciences | -.042 | -1.584† | .094 | -.035 | -1.311 | .316 | -.048 | -1.831 | .112 | .027 | 1.033 | .503 |
| N |  |  | 10,789 |  |  | 10,758 |  |  | 10,754 |  |  | 10,712 |  |  |

*Notes*. Cohen’s *d* $\text{= 2*}\text{z}\text{/}\sqrt{n}$. Significance levels: ****p* < .001, ***p* < .01, **p* < .05, †*p* < .1. Non-parametric pairwise multiple comparison (Dunn, 1964) controlling for the false discovery rate using the Benjamini-Hochberg stepwise adjustments.


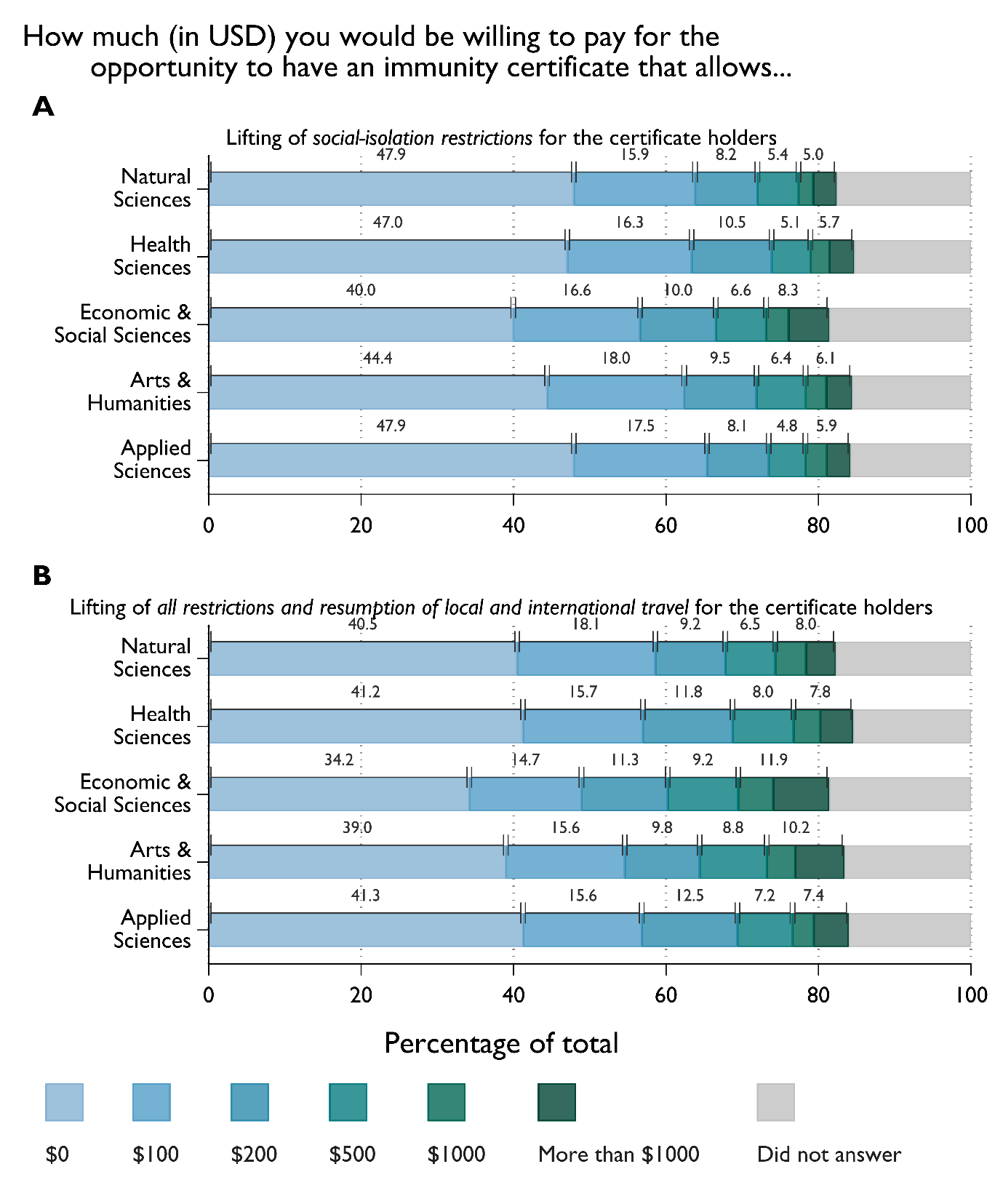


**Supplementary Figure 6.** **Willingness to pay for immunity passports by field of research**. (*A*) lifting social restrictions. N = 10,547 non-missing responses. Kruskal-Wallis equality-of-populations rank test (tie-corrected *χ^2^*(4) = 75.48; *p* < .001). (*B*) lifting travel restrictions. N = 10,530 non-missing responses. Kruskal-Wallis equality-of-populations rank test (tie-corrected *χ^2^*(4) = 86.35; *p* < .001).

**Supplementary Table 2. Differences in willingness-to-pay for COVID-19 immunity certificate across fields.**

|  |  |  | *Lifting social*  *restrictions* | | | *Lifting travel*  *restrictions* | | |
| --- | --- | --- | --- | --- | --- | --- | --- | --- |
|  |  |  | *d* | z-stat. | *p*-val. | *d* | z-stat. | *p*-val. |
| Applied Sciences | vs. | Arts & Humanities | -.052 | -.838 | .402 | -.056 | -.943 | .288 |
| Applied Sciences | vs. | Economic &  Social Sciences | -.121 | -4.645*** | <.001 | -.121 | -4.614*** | <.001 |
| Arts & Humanities | vs. | Economic &  Social Sciences | -.055 | -1.962† | .062 | -.05 | -1.824† | .085 |
| Applied Sciences | vs. | Health Sciences | -.011 | -.393 | .386 | .001 | .062 | .475 |
| Arts & Humanities | vs. | Health Sciences | .020 | .702 | .402 | .031 | 1.105 | .269 |
| Economic &  Social Sciences | vs. | Health Sciences | .160 | 7.883*** | <.001 | .176 | 8.671*** | <.001 |
| Applied Sciences | vs. | Natural Sciences | -.019 | -.522 | .376 | -.015 | -.426 | .372 |
| Arts & Humanities | vs. | Natural Sciences | .026 | .551 | .416 | .032 | .728 | .333 |
| Economic &  Social Sciences | vs. | Natural Sciences | .130 | 5.423*** | <.001 | .134 | 5.528*** | <.001 |
| Health Sciences | vs. | Natural Sciences | -.005 | -.261 | .397 | -.016 | -.723 | .293 |
| N |  |  | 10,547 |  |  | 10,530 |  |  |

*Notes*. Cohen’s *d* $\text{= 2*}\text{z}\text{/}\sqrt{n}$. Significance levels: ****p* < .001, ***p* < .01, **p* < .05, †*p* < .1. Non-parametric pairwise multiple comparison (Dunn, 1964) controlling for the false discovery rate using the Benjamini-Hochberg stepwise adjustments.


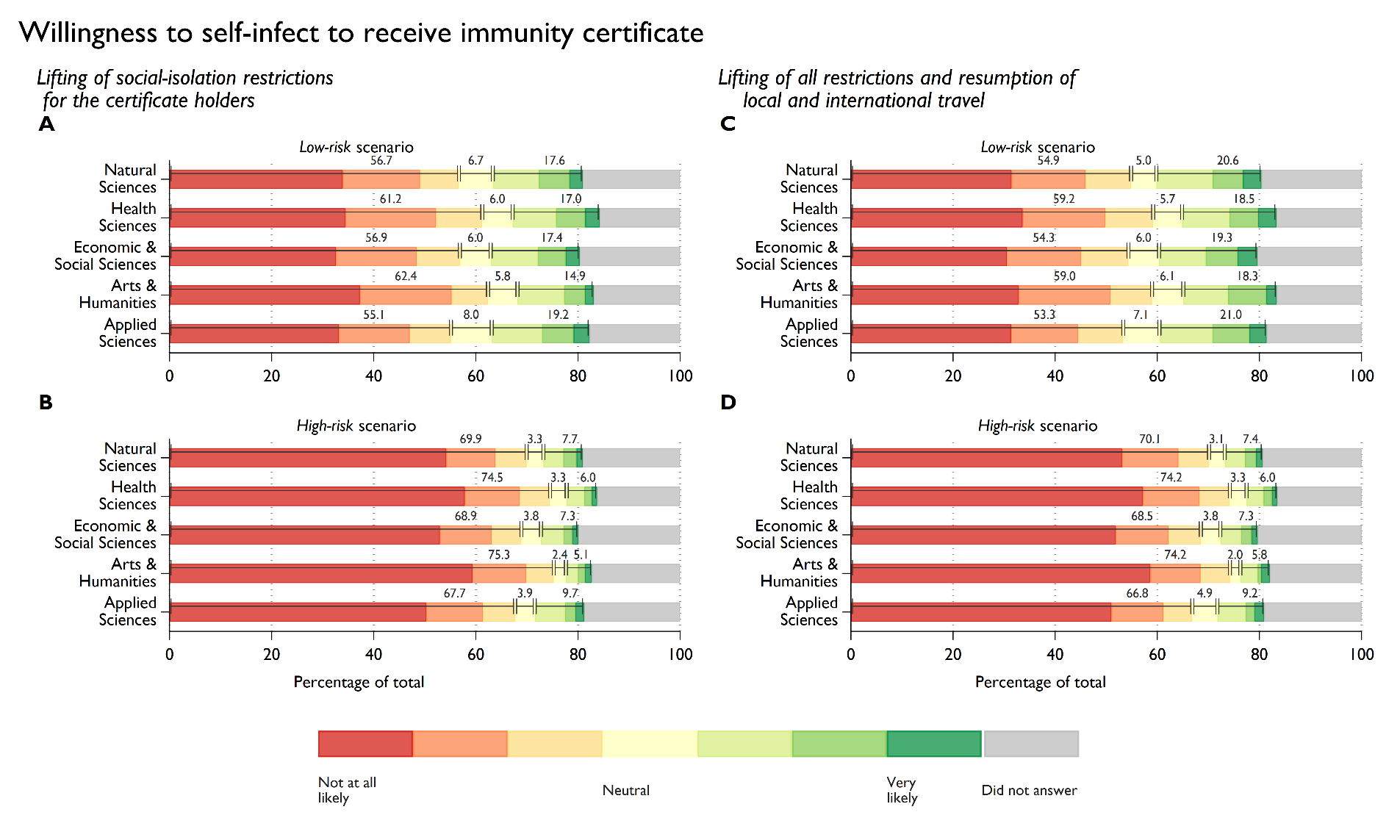


**Supplementary Figure 7.** **Willingness to self-infect for immunity passports by field of research**. (*A*) lifting social restrictions (low-risk scenario). N = 10,424 non-missing responses. Kruskal-Wallis equality-of-populations rank test (tie-corrected *χ^2^*(4) = 6.023; *p* = .197). (*B*) lifting social restrictions (high-risk scenario). N = 10,374 non-missing responses. Kruskal-Wallis equality-of-populations rank test (tie-corrected *χ^2^*(4) = 24.7; *p* < .001). (*C*) lifting travel restrictions (low-risk scenario). N = 10,328 non-missing responses. Kruskal-Wallis equality-of-populations rank test (tie-corrected *χ^2^*(4) = 9.983; *p* = .0407). (*D*) lifting travel restrictions (high-risk scenario). N = 10,336 non-missing responses. Kruskal-Wallis equality-of-populations rank test (tie-corrected *χ^2^*(4) = 23.38; *p* < .001).

**Supplementary Table 3. Differences in willingness to self-infect to receive COVID-19 immunity certificate across fields.**

|  |  |  | *Low-risk scenario* | | | | | | *High-risk scenario* | | | | | |
| --- | --- | --- | --- | --- | --- | --- | --- | --- | --- | --- | --- | --- | --- | --- |
|  |  |  | *Lifting social*  *restrictions* | | | *Lifting travel*  *restrictions* | | | *Lifting social*  *restrictions* | | | *Lifting travel*  *restrictions* | | |
|  |  |  | *d* | z-stat. | *p*-val. | *d* | z-stat. | *p*-val. | *d* | z-stat. | *p*-val. | *d* | z-stat. | *p*-val. |
| Applied Sciences | vs. | Arts & Humanities | .142 | 2.186 | .144 | .073 | 1.117 | .330 | .198 | 3.084** | .003 | .174 | 2.704* | .011 |
| Applied Sciences | vs. | Economic &  Social Sciences | .030 | 1.009 | .196 | .016 | .543 | .367 | .067 | 2.314* | .021 | .041 | 1.401 | .101 |
| Arts & Humanities | vs. | Economic &  Social Sciences | -.057 | -1.837 | .165 | -.029 | -.921 | .357 | -.062 | -2.029* | .035 | -.067 | -2.177* | .037 |
| Applied Sciences | vs. | Health Sciences | .046 | 1.588 | .187 | .059 | 2.023 | .108 | .119 | 4.066*** | <.001 | .100 | 3.408** | .002 |
| Arts & Humanities | vs. | Health Sciences | -.045 | -1.476 | .140 | .001 | .015 | .494 | -.029 | -.929 | .196 | -.029 | -.917 | .200 |
| Economic & Social Sciences | vs. | Health Sciences | .024 | 1.068 | .204 | .062 | 2.740* | .031 | .073 | 3.247** | .003 | .084 | 3.724*** | .001 |
| Applied Sciences | vs. | Natural Sciences | .050 | 1.163 | .204 | .03 | .688 | .351 | .099 | 2.317* | .026 | .073 | 1.672† | .067 |
| Arts & Humanities | vs. | Natural Sciences | -.077 | -1.564 | .147 | -.036 | -.734 | .386 | -.086 | -1.751* | .050 | -.088 | -1.779† | .063 |
| Economic &  Social Sciences | vs. | Natural Sciences | .011 | .402 | .382 | .008 | .310 | .420 | .010 | .394 | .347 | .018 | .644 | .260 |
| Health Sciences | vs. | Natural Sciences | -.010 | -.364 | .358 | -.045 | -1.659 | .162 | -.053 | -1.939* | .038 | -.056 | -2.032* | .042 |
| N |  |  | 10,424 | | | 10,374 | | | 10,328 | | | 10,336 | | |

*Notes*. Cohen’s *d* $\text{= 2*}\text{z}\text{/}\sqrt{n}$. Significance levels: ****p* < .001, ***p* < .01, **p* < .05, †*p* < .1. Non-parametric pairwise multiple comparison (Dunn, 1964) controlling for the false discovery rate using the Benjamini-Hochberg stepwise adjustments.

# US and non-US difference

**Supplementary Table 4. Difference in views on immunity certificates between US and non-US based scientists.**

| *Non-US* vs. *US* | *d* | *z*-stat. | *p-*val. |
| --- | --- | --- | --- |
| Good for public health | -.117 | -6.06*** | <.001 |
| Good for the economy | -.113 | -5.84*** | <.001 |
| Fair to others who do not have immunity | -.037 | -1.93† | .0533 |
| Increasing inequality | .053 | 2.75** | .0059 |
| *Willingness to pay* |  |  |  |
| Lifting of social-isolation restrictions | -.250 | -12.82*** | <.001 |
| Lifting of all restrictions and resumption of local and international travel | -.188 | -9.63*** | <.001 |
| *Willingness to self-infect* |  |  |  |
| *Low-risk* scenario |  |  |  |
| Lifting of social-isolation restrictions | .048 | 2.43* | .0152 |
| Lifting of all restrictions and resumption of local and international travel | .070 | 3.56*** | <.001 |
| *High-risk* scenario |  |  |  |
| Lifting of social-isolation restrictions | .195 | 9.92*** | <.001 |
| Lifting of all restrictions and resumption of local and international travel | .189 | 9.63*** | <.001 |

*Notes*. Wilcoxon rank sum test (two-tailed). Cohen’s *d* $\text{= 2*}\text{z}\text{/}\sqrt{n}$. Significance levels: ****p* < .001, ***p* < .01, **p* < .05, †*p* < .1.

**
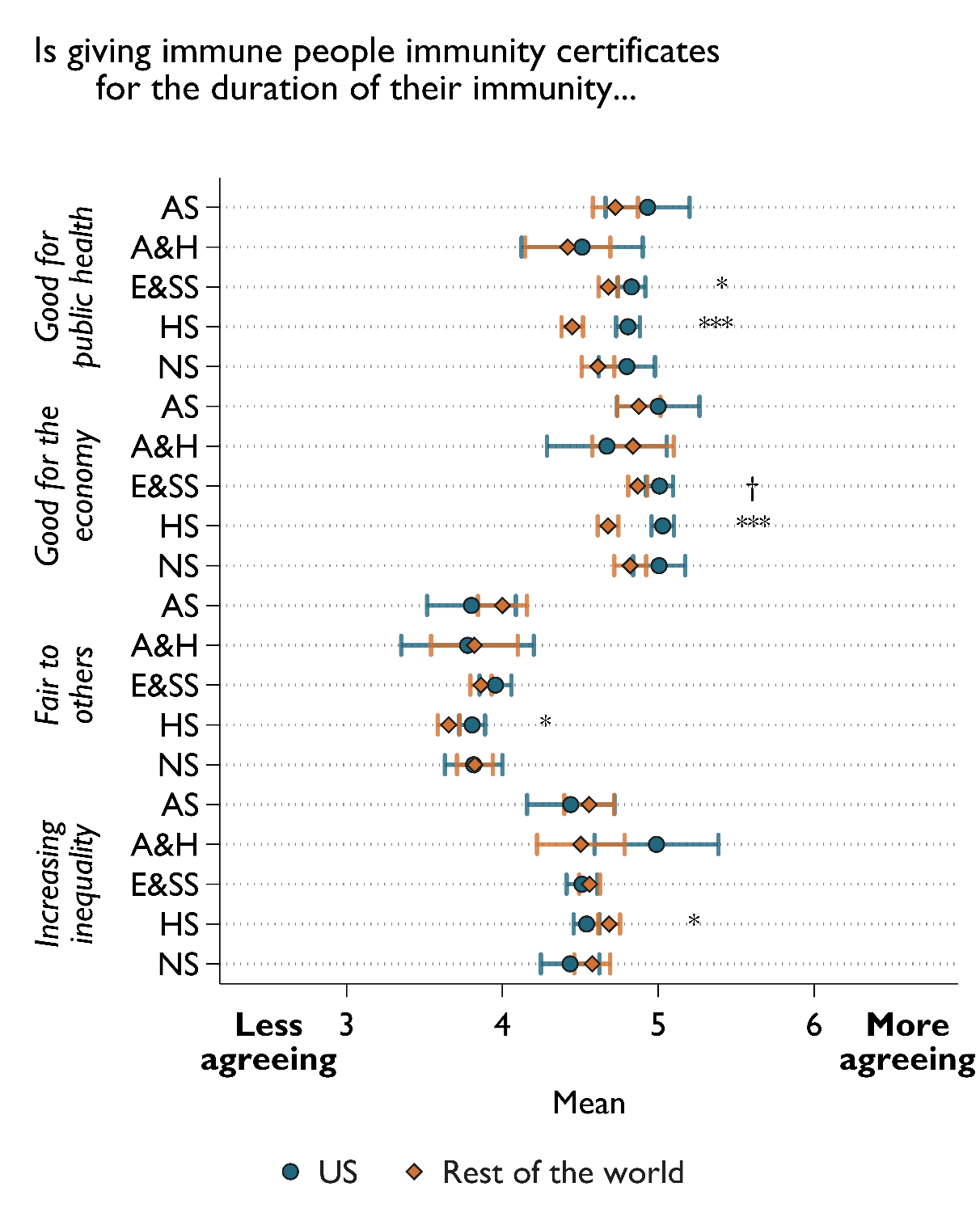
**

**Supplementary Figure 8.** **Difference in opinion on immunity certificates between US and non-US based scientists by field**. *AS* = Applied Sciences; *A&H* = Arts & Humanities; *E&SS* = Economic & Social Sciences; *HS* = Health Sciences; *NS* = Natural Sciences. Two-sample mean comparison with *t-*test (two-tailed). Error bars represent 95% confidence intervals. Significance levels: ****p* < .001, ***p* < .01, **p* < .05, †*p* < .1 based on *p*-values with Bonferroni correction for multiple-comparison. Results are robust to using the Wilcoxon rank sum test.

**
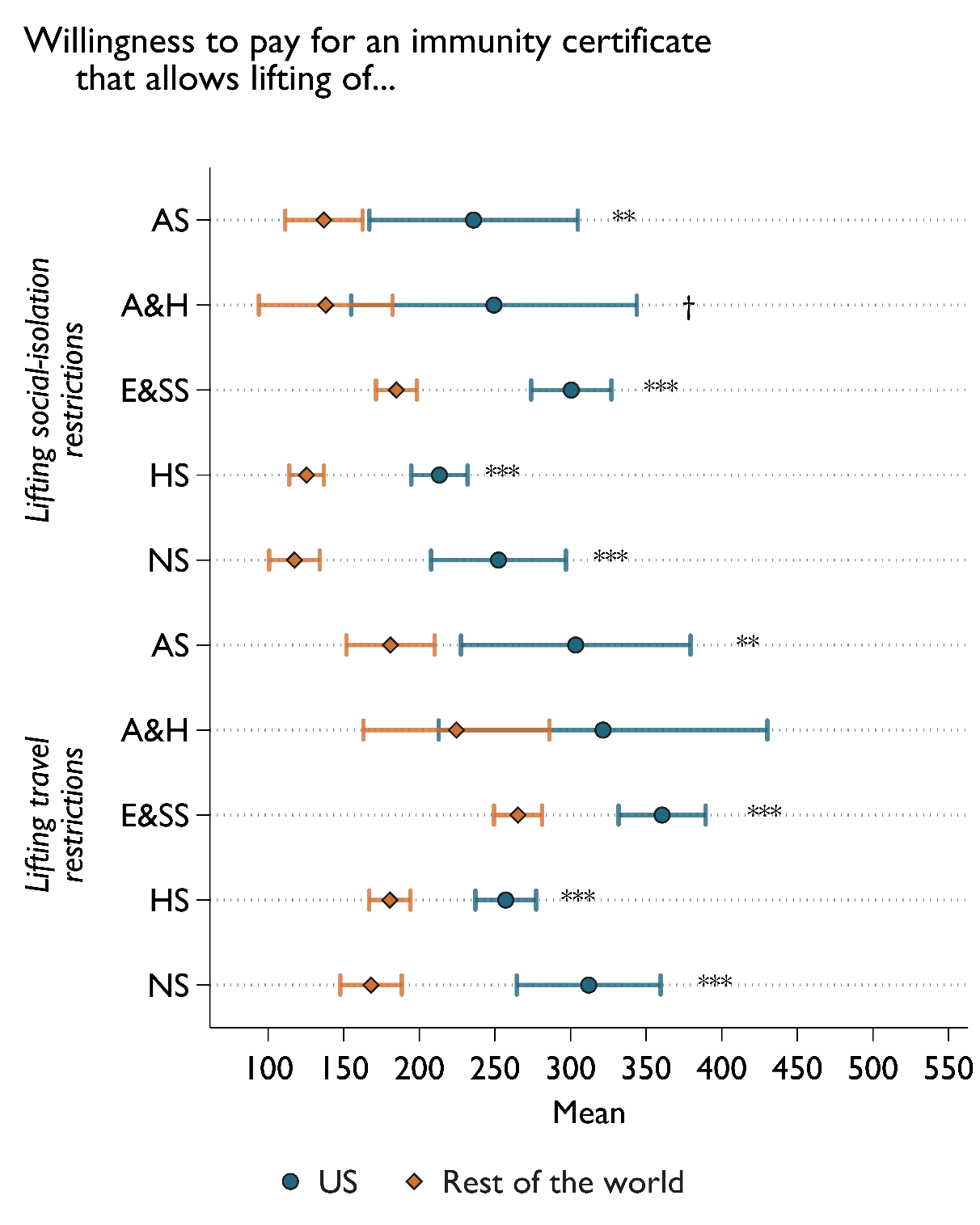
**

**Supplementary Figure 9.** **Difference in willingness-to-pay for immunity certificates between US and non-US based scientists by field**. *AS* = Applied Sciences; *A&H* = Arts & Humanities; *E&SS* = Economic & Social Sciences; *HS* = Health Sciences; *NS* = Natural Sciences. Two-sample mean comparison with *t-*test (two-tailed). Error bars represent 95% confidence intervals. Significance levels: ****p* < .001, ***p* < .01, **p* < .05, †*p* < .1 based on *p*-values with Bonferroni correction for multiple-comparison. Results are robust to using the Wilcoxon rank sum test.

**
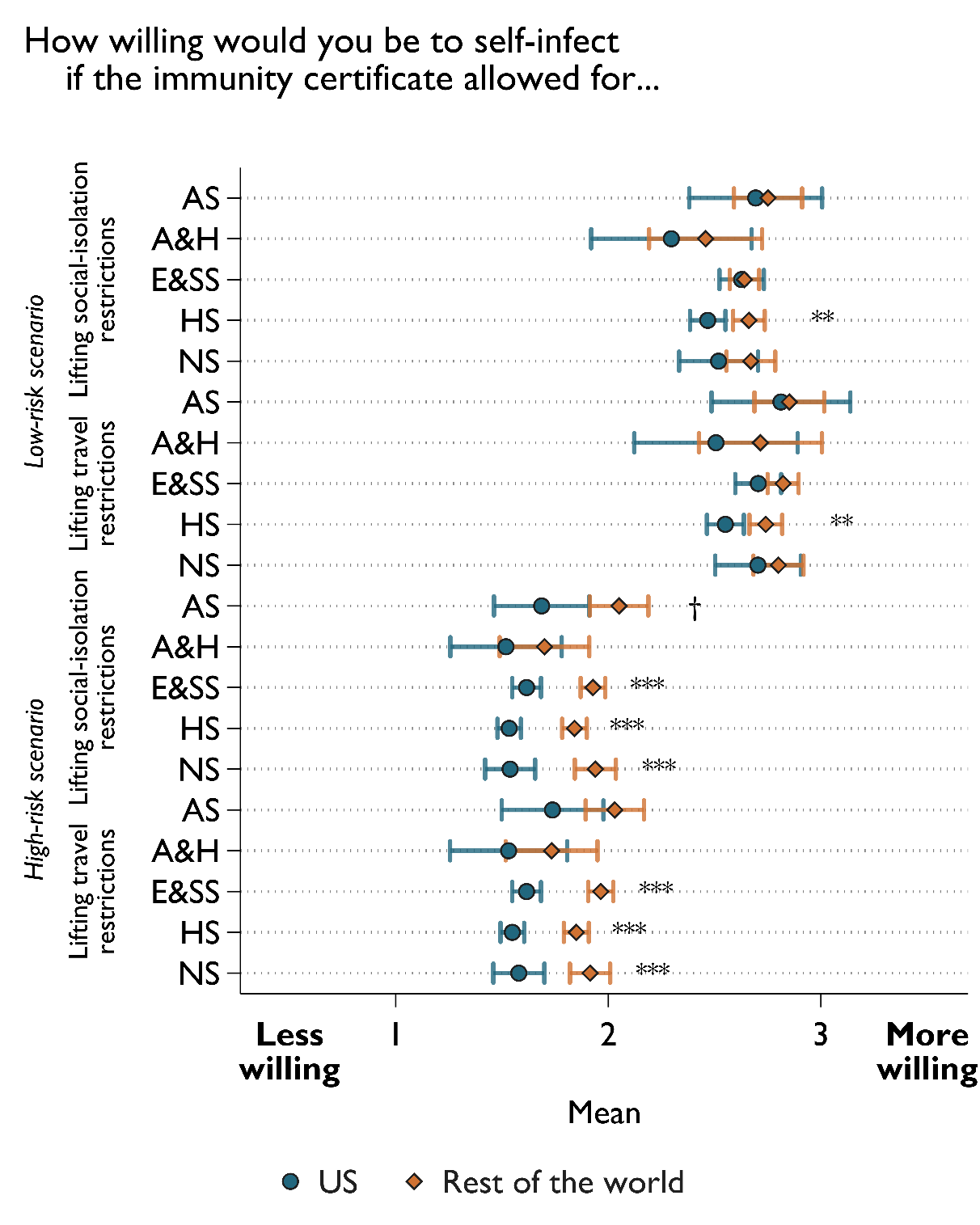
**

**Supplementary Figure 10.** **Difference in willingness to self-infect for immunity certificates between US and non-US based scientists by field**. *AS* = Applied Sciences; *A&H* = Arts & Humanities; *E&SS* = Economic & Social Sciences; *HS* = Health Sciences; *NS* = Natural Sciences. Two-sample mean comparison with *t-*test (two-tailed). Error bars represent 95% confidence intervals. Significance levels: ****p* < .001, ***p* < .01, **p* < .05, †*p* < .1 based on *p*-values with Bonferroni correction for multiple-comparison. Results are robust to using the Wilcoxon rank sum test (Supplementary Table 5).

**Supplementary Table 5. Difference in views on immunity certificates between US and non-US based scientists, by field.**

| *Non-US* vs. *US* | *d* | *z*-stat. | *p-*val. |
| --- | --- | --- | --- |
| Good for public health |  |  |  |
| *Applied Sciences* | -.091 | -1.209 | .227 |
| *Arts & Humanities* | -.058 | -.46 | .645 |
| *Economic & Social Sciences* | -.069 | -2.2* | .0278 |
| *Health Sciences* | -.193 | -6.249*** | <.001 |
| *Natural Sciences* | -.099 | -1.868† | .0618 |
| Good for the economy |  |  |  |
| *Applied Sciences* | -.054 | -.717 | .473 |
| *Arts & Humanities* | .092 | .731 | .465 |
| *Economic & Social Sciences* | -.068 | -2.183* | .029 |
| *Health Sciences* | -.198 | -6.410*** | <.001 |
| *Natural Sciences* | -.091 | -1.707† | .0878 |
| Fair to others who do not have immunity |  |  |  |
| *Applied Sciences* | .089 | 1.18 | .238 |
| *Arts & Humanities* | .018 | .14 | .889 |
| *Economic & Social Sciences* | -.048 | -1.533 | .125 |
| *Health Sciences* | -.083 | -2.683** | .00729 |
| *Natural Sciences* | .001 | .014 | .988 |
| Increasing inequality |  |  |  |
| *Applied Sciences* | .08 | 1.062 | .288 |
| *Arts & Humanities* | -.264 | -2.092* | .0365 |
| *Economic & Social Sciences* | .032 | 1.007 | .314 |
| *Health Sciences* | .09 | 2.902** | .00371 |
| *Natural Sciences* | .083 | 1.554 | .12 |
| *Willingness to pay* |  |  |  |
| Lifting of social-isolation restrictions |  |  |  |
| *Applied Sciences* | -.219 | -2.889** | .00387 |
| *Arts & Humanities* | -.312 | -2.462* | .0138 |
| *Economic & Social Sciences* | -.222 | -7.014*** | <.001 |
| *Health Sciences* | -.277 | -8.870*** | <.001 |
| *Natural Sciences* | -.32 | -6.007*** | <.001 |
| Lifting of all restrictions and resumption of local and international travel |  |  |  |
| *Applied Sciences* | -.228 | -3.007** | .00264 |
| *Arts & Humanities* | -.196 | -1.539 | .124 |
| *Economic & Social Sciences* | -.134 | -4.240*** | <.001 |
| *Health Sciences* | -.213 | -6.808*** | <.001 |
| *Natural Sciences* | -.343 | -6.425*** | <.001 |
| *Willingness to self-infect* |  |  |  |
| *Low-risk* scenario |  |  |  |
| Lifting of social-isolation restrictions |  |  |  |
| *Applied Sciences* | .016 | .209 | .834 |
| *Arts & Humanities* | .085 | .666 | .506 |
| *Economic & Social Sciences* | .005 | .164 | .87 |
| *Health Sciences* | .075 | 2.406* | .0161 |
| *Natural Sciences* | .055 | 1.031 | .303 |
| Lifting of all restrictions and resumption of local and international travel |  |  |  |
| *Applied Sciences* | .017 | .214 | .83 |
| *Arts & Humanities* | .075 | .589 | .556 |
| *Economic & Social Sciences* | .054 | 1.681† | .0928 |
| *Health Sciences* | .082 | 2.612** | .00901 |
| *Natural Sciences* | .048 | .894 | .371 |
| *High-risk* scenario |  |  |  |
| Lifting of social-isolation restrictions |  |  |  |
| *Applied Sciences* | .187 | 2.420* | .0155 |
| *Arts & Humanities* | .121 | .947 | .343 |
| *Economic & Social Sciences* | .151 | 4.731*** | <.001 |
| *Health Sciences* | .206 | 6.573*** | <.001 |
| *Natural Sciences* | .221 | 4.106*** | <.001 |
| Lifting of all restrictions and resumption of local and international travel |  |  |  |
| *Applied Sciences* | .157 | 2.039* | .0415 |
| *Arts & Humanities* | .186 | 1.449 | .147 |
| *Economic & Social Sciences* | .177 | 5.539*** | <.001 |
| *Health Sciences* | .192 | 6.118*** | <.001 |
| *Natural Sciences* | .165 | 3.067** | .00216 |

*Notes*. Wilcoxon rank sum test (two-tailed). Cohen’s *d* $\text{= 2*}\text{z}\text{/}\sqrt{n}$. Significance levels: ****p* < .001, ***p* < .01, **p* < .05, †*p* < .1 based on *p*-values with Bonferroni correction for multiple-comparison.

# Consensus

**
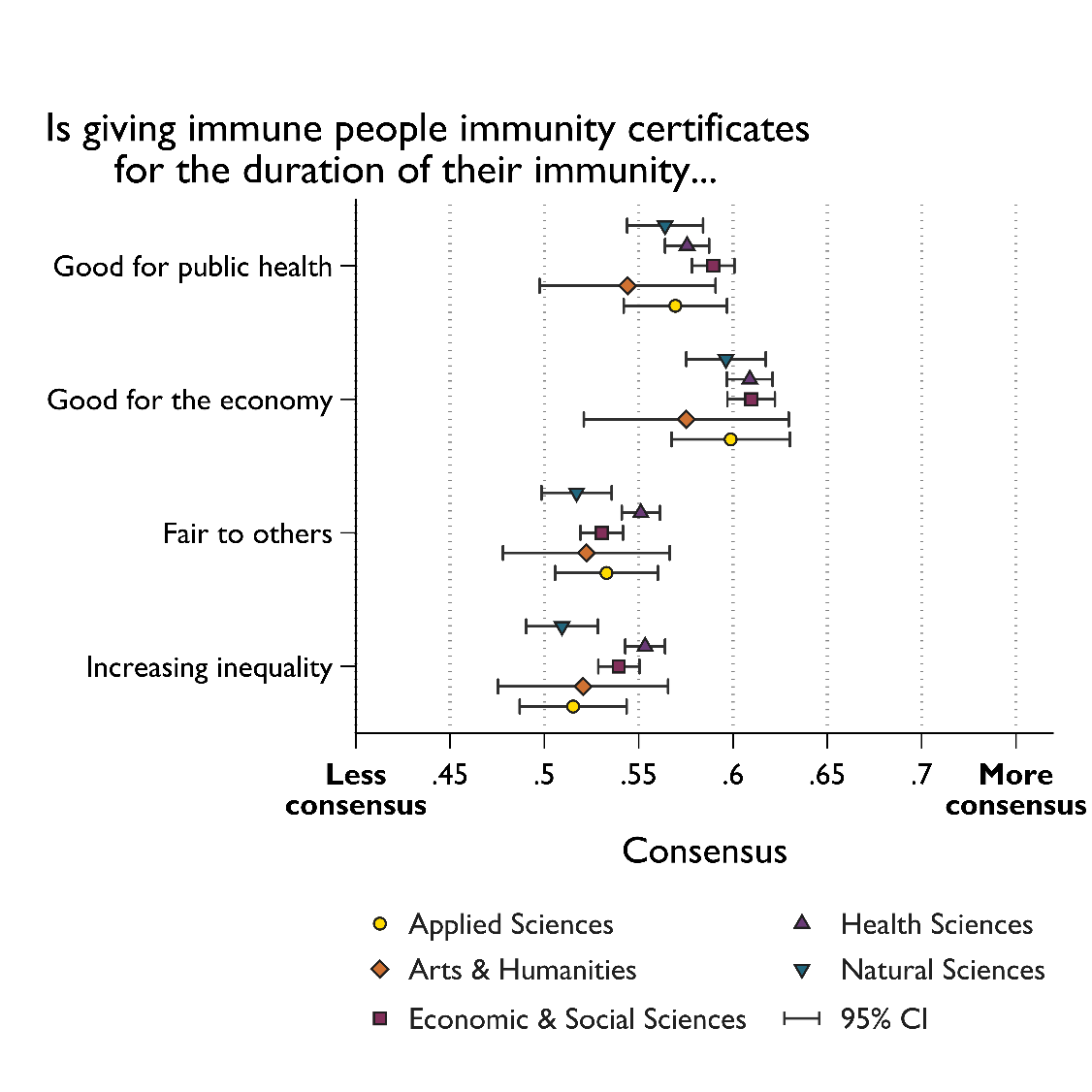
**

**Supplementary Figure 11.** **Consensus on perceived benefits to public health and economy, fairness, and societal inequality of immunity certificate within field**. Error bars represent 95% confidence intervals obtained from bootstrap resampling with 300 replications. Null responses are excluded from the calculation of consensus.

**
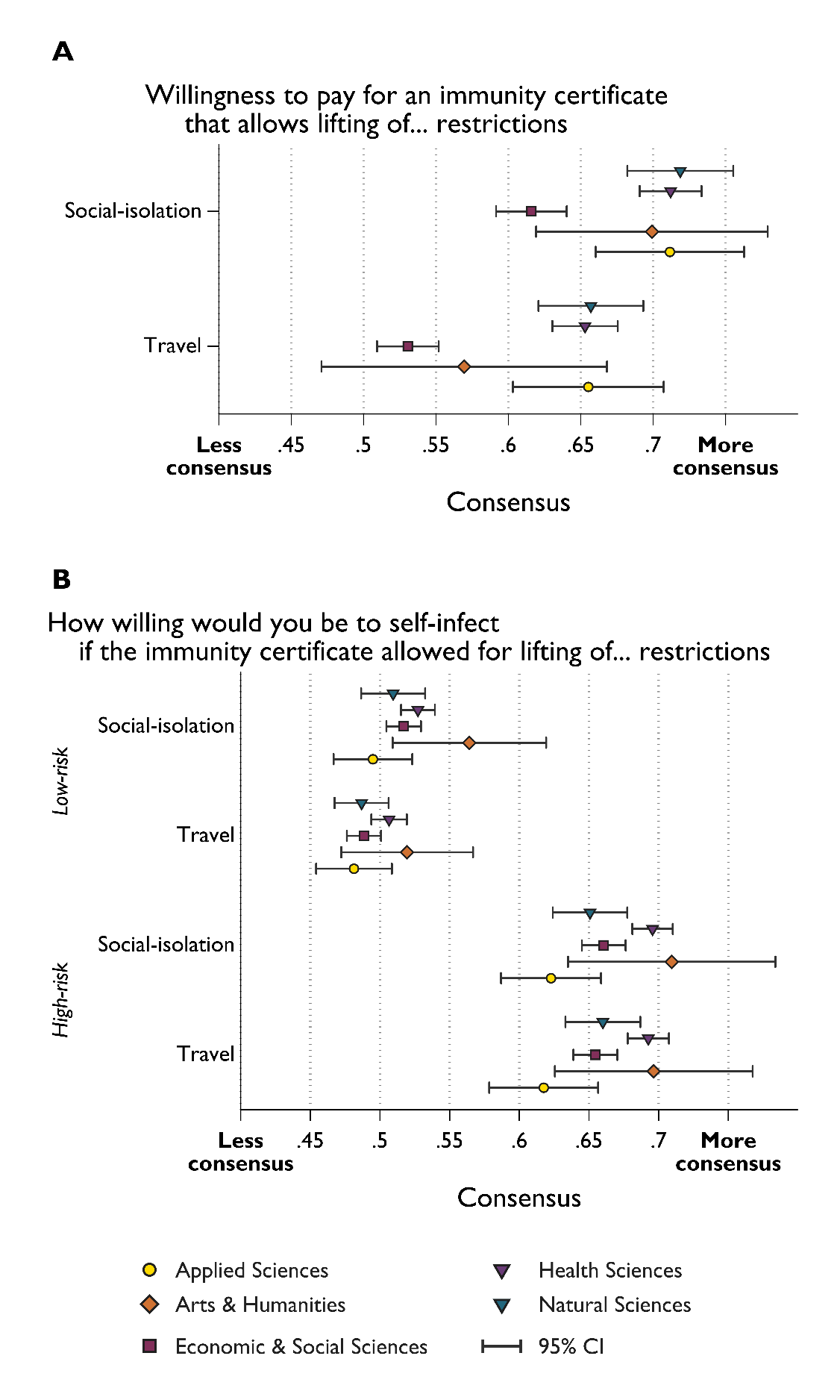
**

**Supplementary Figure 12.** **Consensus on willingness to pay (*A*) and self-infect (*B*) for immunity certificate for lifting social-isolation and travel restrictions within field**. Error bars represent 95% confidence intervals obtained from bootstrap resampling with 300 replications. Null responses are excluded from the calculation of consensus.

**Supplementary Table 6. Differences in the level of consensus on perceived benefits to public health and economy, fairness, and societal inequality of immunity certificate across fields.**

| Field 1 | Field 2 | Mean 1 | SD 1 | Mean 2 | SD 2 | *p* (unadj.) | *p* (adjusted) |
| --- | --- | --- | --- | --- | --- | --- | --- |
| **Good for public health** | | | | | | | |
| *AS* | *A&H* | 0.569 | 0.372 | 0.544 | 0.379 | 0.360 | 1 |
| *AS* | *E&SS* | 0.569 | 0.372 | 0.59 | 0.367 | 0.180 | 1 |
| *AS* | *HS* | 0.569 | 0.372 | 0.576 | 0.385 | 0.690 | 1 |
| *AS* | *NS* | 0.569 | 0.372 | 0.564 | 0.388 | 0.760 | 1 |
| *A&H* | *E&SS* | 0.544 | 0.379 | 0.59 | 0.367 | 0.057 | 0.570 |
| *A&H* | *HS* | 0.544 | 0.379 | 0.576 | 0.385 | 0.210 | 1 |
| *A&H* | *NS* | 0.544 | 0.379 | 0.564 | 0.388 | 0.450 | 1 |
| *E&SS* | *HS* | 0.590 | 0.367 | 0.576 | 0.385 | 0.094 | 0.940 |
| *E&SS* | *NS* | 0.590 | 0.367 | 0.564 | 0.388 | 0.026 | 0.260 |
| *HS* | *NS* | 0.576 | 0.385 | 0.564 | 0.388 | 0.320 | 1 |
| **Good for the economy** | | | | | | | |
| *AS* | *E&SS* | 0.599 | 0.425 | 0.575 | 0.44 | 0.460 | 1 |
| *AS* | *HS* | 0.599 | 0.425 | 0.610 | 0.411 | 0.520 | 1 |
| *AS* | *NS* | 0.599 | 0.425 | 0.609 | 0.400 | 0.540 | 1 |
| *A&H* | *E&SS* | 0.599 | 0.425 | 0.596 | 0.406 | 0.900 | 1 |
| *A&H* | *HS* | 0.575 | 0.440 | 0.610 | 0.411 | 0.200 | 1 |
| *A&H* | *NS* | 0.575 | 0.440 | 0.609 | 0.400 | 0.200 | 1 |
| *E&SS* | *HS* | 0.575 | 0.440 | 0.596 | 0.406 | 0.450 | 1 |
| *E&SS* | *NS* | 0.610 | 0.411 | 0.609 | 0.400 | 0.940 | 1 |
| *HS* | *NS* | 0.610 | 0.411 | 0.596 | 0.406 | 0.290 | 1 |
| **Fair to others who do not have immunity** | | | | | | | |
| *AS* | *A&H* | 0.533 | 0.369 | 0.522 | 0.358 | 0.690 | 1 |
| *AS* | *E&SS* | 0.533 | 0.369 | 0.530 | 0.369 | 0.870 | 1 |
| *AS* | *HS* | 0.533 | 0.369 | 0.551 | 0.334 | 0.190 | 1 |
| *AS* | *NS* | 0.533 | 0.369 | 0.517 | 0.360 | 0.340 | 1 |
| *A&H* | *E&SS* | 0.522 | 0.358 | 0.530 | 0.369 | 0.740 | 1 |
| *A&H* | *HS* | 0.522 | 0.358 | 0.551 | 0.334 | 0.190 | 1 |
| *A&H* | *NS* | 0.522 | 0.358 | 0.517 | 0.360 | 0.830 | 1 |
| *E&SS* | *HS* | 0.530 | 0.369 | 0.551 | 0.334 | 0.007 | .074† |
| *E&SS* | *NS* | 0.530 | 0.369 | 0.517 | 0.360 | 0.240 | 1 |
| *HS* | *NS* | 0.551 | 0.334 | 0.517 | 0.360 | 0.001 | .011* |
| **Increasing inequality** | | | | | | | |
| *AS* | *A&H* | 0.515 | 0.383 | 0.521 | 0.365 | 0.850 | 1 |
| *AS* | *E&SS* | 0.515 | 0.383 | 0.539 | 0.354 | 0.099 | 0.99 |
| *AS* | *HS* | 0.515 | 0.383 | 0.553 | 0.349 | 0.0082 | .082† |
| *AS* | *NS* | 0.515 | 0.383 | 0.509 | 0.365 | 0.730 | 1 |
| *A&H* | *E&SS* | 0.521 | 0.365 | 0.539 | 0.354 | 0.410 | 1 |
| *A&H* | *HS* | 0.521 | 0.365 | 0.553 | 0.349 | 0.150 | 1 |
| *A&H* | *NS* | 0.521 | 0.365 | 0.509 | 0.365 | 0.650 | 1 |
| *E&SS* | *HS* | 0.539 | 0.354 | 0.553 | 0.349 | 0.072 | 0.72 |
| *E&SS* | *NS* | 0.539 | 0.354 | 0.509 | 0.365 | 0.006 | .063† |
| *HS* | *NS* | 0.553 | 0.349 | 0.509 | 0.365 | <.001 | <.001*** |

*Notes*. *AS* = Applied Sciences; *A&H* = Arts & Humanities; *E&SS* = Economic & Social Sciences; *HS* = Health Sciences; *NS* = Natural Sciences. Two-tailed *t-*test. Significance levels: ****p* < 0.001, ***p* < 0.01, **p* < 0.05, †*p* < 0.1 based on p-values with Bonferroni correction for multiple-comparison. Standard deviations are computed using bootstrap resampling with 300 replications.

**Supplementary Table 7. Differences in the level of consensus on willingness to pay and willingness to self-infect for immunity certificate across fields.**

| Field 1 | Field 2 | Mean 1 | SD 1 | Mean 2 | SD 2 | *p* (unadj.) | *p* (adjusted) |
| --- | --- | --- | --- | --- | --- | --- | --- |
| **Willingness to pay: Lifting of social-isolation restrictions** | | | | | | | |
| *AS* | *A&H* | 0.712 | 0.691 | 0.699 | 0.644 | 0.8 | 1 |
| *AS* | *E&SS* | 0.712 | 0.691 | 0.616 | 0.787 | 0.0026 | .026* |
| *AS* | *HS* | 0.712 | 0.691 | 0.712 | 0.699 | 0.99 | 1 |
| *AS* | *NS* | 0.712 | 0.691 | 0.719 | 0.701 | 0.82 | 1 |
| *A&H* | *E&SS* | 0.699 | 0.644 | 0.616 | 0.787 | 0.1 | 1 |
| *A&H* | *HS* | 0.699 | 0.644 | 0.712 | 0.699 | 0.78 | 1 |
| *A&H* | *NS* | 0.699 | 0.644 | 0.719 | 0.701 | 0.68 | 1 |
| *E&SS* | *HS* | 0.616 | 0.787 | 0.712 | 0.699 | <.001 | <.001*** |
| *E&SS* | *NS* | 0.616 | 0.787 | 0.719 | 0.701 | <.001 | <.001*** |
| *HS* | *NS* | 0.712 | 0.699 | 0.719 | 0.701 | 0.76 | 1 |
| **Willingness to pay: Lifting of all restrictions and resumption of local and international travel** | | | | | | | |
| *AS* | *A&H* | 0.655 | 0.701 | 0.569 | 0.789 | 0.11 | 1 |
| *AS* | *E&SS* | 0.655 | 0.701 | 0.531 | 0.686 | <.001 | <.001*** |
| *AS* | *HS* | 0.655 | 0.701 | 0.653 | 0.738 | 0.94 | 1 |
| *AS* | *NS* | 0.655 | 0.701 | 0.657 | 0.693 | 0.96 | 1 |
| *A&H* | *E&SS* | 0.569 | 0.789 | 0.531 | 0.686 | 0.39 | 1 |
| *A&H* | *HS* | 0.569 | 0.789 | 0.653 | 0.738 | 0.086 | 0.86 |
| *A&H* | *NS* | 0.569 | 0.789 | 0.657 | 0.693 | 0.074 | 0.74 |
| *E&SS* | *HS* | 0.531 | 0.686 | 0.653 | 0.738 | <.001 | <.001*** |
| *E&SS* | *NS* | 0.531 | 0.686 | 0.657 | 0.693 | <.001 | <.001*** |
| *HS* | *NS* | 0.653 | 0.738 | 0.657 | 0.693 | 0.86 | 1 |
| **Willingness to self-infect: Lifting of social-isolation restrictions (Low-risk scenario)** | | | | | | | |
| *AS* | *A&H* | 0.495 | 0.376 | 0.564 | 0.44 | 0.019 | 0.190 |
| *AS* | *E&SS* | 0.495 | 0.376 | 0.517 | 0.397 | 0.180 | 1 |
| *AS* | *HS* | 0.495 | 0.376 | 0.527 | 0.397 | 0.047 | 0.470 |
| *AS* | *NS* | 0.495 | 0.376 | 0.509 | 0.436 | 0.460 | 1 |
| *A&H* | *E&SS* | 0.564 | 0.440 | 0.517 | 0.397 | 0.073 | 0.730 |
| *A&H* | *HS* | 0.564 | 0.440 | 0.527 | 0.397 | 0.160 | 1 |
| *A&H* | *NS* | 0.564 | 0.440 | 0.509 | 0.436 | 0.071 | 0.710 |
| *E&SS* | *HS* | 0.517 | 0.397 | 0.527 | 0.397 | 0.250 | 1 |
| *E&SS* | *NS* | 0.517 | 0.397 | 0.509 | 0.436 | 0.550 | 1 |
| *HS* | *NS* | 0.527 | 0.397 | 0.509 | 0.436 | 0.160 | 1 |
| **Willingness to self-infect: Lifting of all restrictions and resumption of local and international travel (Low-risk scenario)** | | | | | | | |
| *AS* | *A&H* | 0.481 | 0.360 | 0.52 | 0.378 | 0.160 | 1 |
| *AS* | *E&SS* | 0.481 | 0.360 | 0.489 | 0.390 | 0.660 | 1 |
| *AS* | *HS* | 0.481 | 0.360 | 0.507 | 0.413 | 0.140 | 1 |
| *AS* | *NS* | 0.481 | 0.360 | 0.487 | 0.367 | 0.750 | 1 |
| *A&H* | *E&SS* | 0.52 | 0.378 | 0.489 | 0.39 | 0.230 | 1 |
| *A&H* | *HS* | 0.52 | 0.378 | 0.507 | 0.413 | 0.630 | 1 |
| *A&H* | *NS* | 0.52 | 0.378 | 0.487 | 0.367 | 0.200 | 1 |
| *E&SS* | *HS* | 0.489 | 0.390 | 0.507 | 0.413 | 0.046 | 0.46 |
| *E&SS* | *NS* | 0.489 | 0.390 | 0.487 | 0.367 | 0.890 | 1 |
| *HS* | *NS* | 0.507 | 0.413 | 0.487 | 0.367 | 0.120 | 1 |
| **Willingness to self-infect: Lifting of social-isolation restrictions (High-risk scenario)** | | | | | | | |
| *AS* | *A&H* | 0.623 | 0.475 | 0.709 | 0.593 | 0.023 | 0.23 |
| *AS* | *E&SS* | 0.623 | 0.475 | 0.661 | 0.502 | 0.069 | 0.69 |
| *AS* | *HS* | 0.623 | 0.475 | 0.696 | 0.471 | <.001 | .0021** |
| *AS* | *NS* | 0.623 | 0.475 | 0.651 | 0.507 | 0.230 | 1 |
| *A&H* | *E&SS* | 0.709 | 0.593 | 0.661 | 0.502 | 0.150 | 1 |
| *A&H* | *HS* | 0.709 | 0.593 | 0.696 | 0.471 | 0.660 | 1 |
| *A&H* | *NS* | 0.709 | 0.593 | 0.651 | 0.507 | 0.110 | 1 |
| *E&SS* | *HS* | 0.661 | 0.502 | 0.696 | 0.471 | 0.0013 | .013* |
| *E&SS* | *NS* | 0.661 | 0.502 | 0.651 | 0.507 | 0.530 | 1 |
| *HS* | *NS* | 0.696 | 0.471 | 0.651 | 0.507 | 0.0027 | .027* |
| **Willingness to self-infect: Lifting of all restrictions and resumption of local and international travel (High-risk scenario)** | | | | | | | |
| *AS* | *A&H* | 0.618 | 0.516 | 0.697 | 0.563 | 0.047 | 0.470 |
| *AS* | *E&SS* | 0.618 | 0.516 | 0.655 | 0.502 | 0.079 | 0.790 |
| *AS* | *HS* | 0.618 | 0.516 | 0.693 | 0.482 | <.001 | .002** |
| *AS* | *NS* | 0.618 | 0.516 | 0.66 | 0.509 | 0.078 | 0.780 |
| *A&H* | *E&SS* | 0.697 | 0.563 | 0.655 | 0.502 | 0.210 | 1 |
| *A&H* | *HS* | 0.697 | 0.563 | 0.693 | 0.482 | 0.900 | 1 |
| *A&H* | *NS* | 0.697 | 0.563 | 0.66 | 0.509 | 0.310 | 1 |
| *E&SS* | *HS* | 0.655 | 0.502 | 0.693 | 0.482 | <.001 | .006** |
| *E&SS* | *NS* | 0.655 | 0.502 | 0.66 | 0.509 | 0.730 | 1 |
| *HS* | *NS* | 0.693 | 0.482 | 0.66 | 0.509 | 0.032 | 0.320 |

*Notes*. *AS* = Applied Sciences; *A&H* = Arts & Humanities; *E&SS* = Economic & Social Sciences; *HS* = Health Sciences; *NS* = Natural Sciences. Two-tailed *t-*test. Significance levels: ****p* < 0.001, ***p* < 0.01, **p* < 0.05, †*p* < 0.1 based on p-values with Bonferroni correction for multiple-comparison. Standard deviations are computed using bootstrap resampling with 300 replications.

**
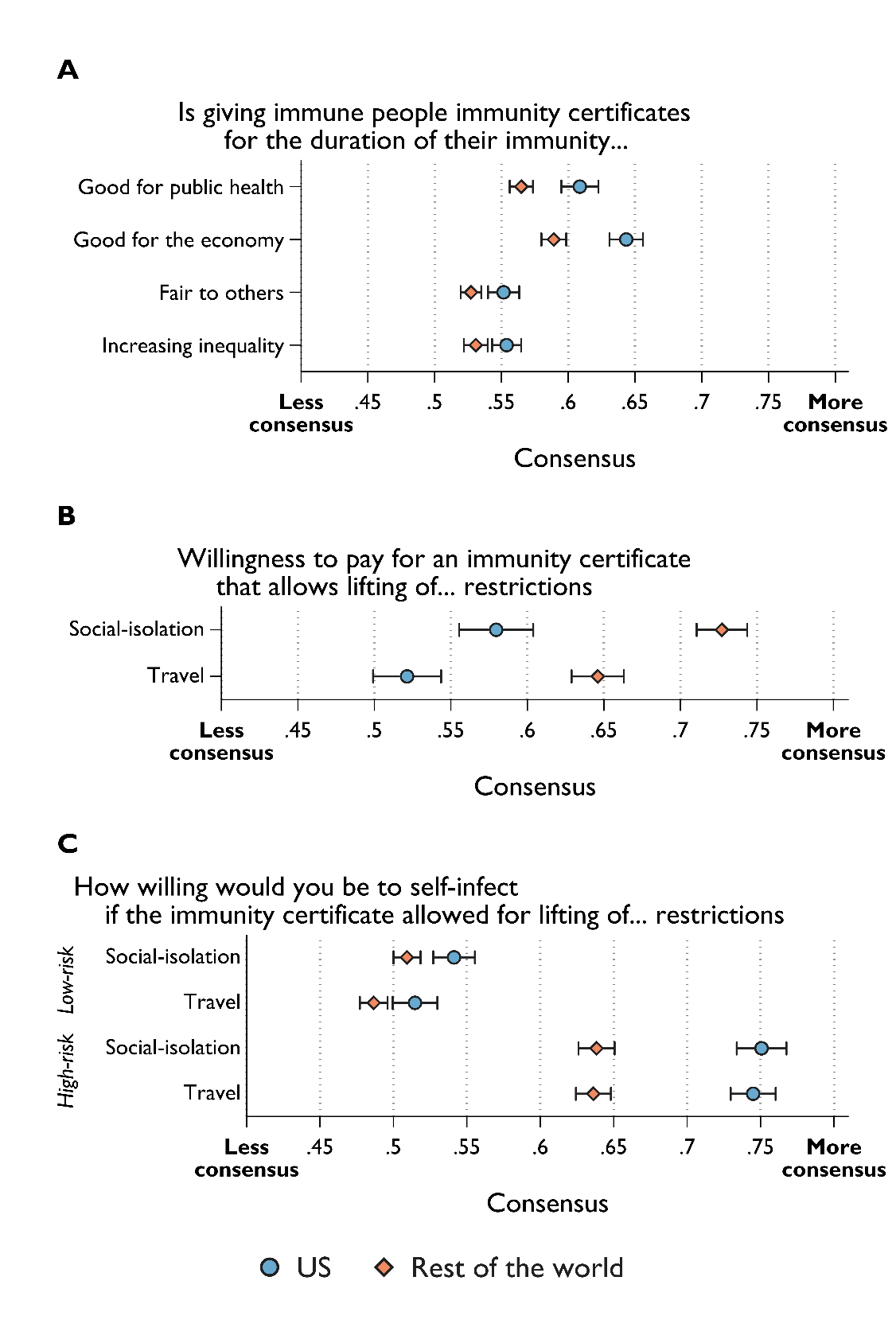
**

**Supplementary Figure 13.** **Differences in the level of consensus between US and non-US scientists**. Error bars represent 95% confidence intervals obtained from bootstrap resampling with 300 replications. Null responses are excluded from the calculation of consensus.

**Supplementary Table 8. Differences in the level of consensus between US and non-US scientists.**

|  | Non-US | | US | |  | |  |
| --- | --- | --- | --- | --- | --- | --- | --- |
|  | M 1 | SD 1 | M 2 | SD 2 | *p*  (unadj.) | *p*  (adjusted) | |
| Good for public health | 0.565 | 0.376 | 0.609 | 0.425 | <.001 | <.001*** | |
| Good for the economy | 0.589 | 0.402 | 0.643 | 0.384 | <.001 | <.001*** | |
| Fair to others who do not have immunity | 0.527 | 0.334 | 0.552 | 0.357 | <.001 | .005*** | |
| Increasing inequality | 0.531 | 0.382 | 0.554 | 0.332 | 0.0022 | .022** | |
| Willingness to pay: Lifting of social-isolation restrictions | 0.727 | 0.709 | 0.58 | 0.73 | <.001 | <.001*** | |
| Willingness to pay: Lifting of all restrictions and resumption of local and international travel | 0.646 | 0.732 | 0.521 | 0.671 | <.001 | <.001*** | |
| Willingness to self-infect: Lifting of social-isolation restrictions (*Low-risk* scenario) | 0.509 | 0.390 | 0.541 | 0.427 | <.001 | .001*** | |
| Willingness to self-infect: Lifting of all restrictions and resumption of local and international travel (*Low-risk* scenario) | 0.487 | 0.399 | 0.515 | 0.456 | 0.001 | .0130** | |
| Willingness to self-infect: Lifting of social-isolation restrictions (*High-risk* scenario) | 0.638 | 0.521 | 0.751 | 0.508 | <.001 | <.001*** | |
| Willingness to self-infect: Lifting of all restrictions and resumption of local and international travel (*High-risk* scenario) | 0.636 | 0.504 | 0.745 | 0.460 | <.001 | <.001*** | |

*Notes*. *AS* = Applied Sciences; *A&H* = Arts & Humanities; *E&SS* = Economic & Social Sciences; *HS* = Health Sciences; *NS* = Natural Sciences. Two-tailed *t-*test. Significance levels: ****p* < 0.001, ***p* < 0.01, **p* < 0.05, †*p* < 0.1 based on p-values with Bonferroni correction for multiple-comparison. Standard deviations are computed using bootstrap resampling with 300 replications.


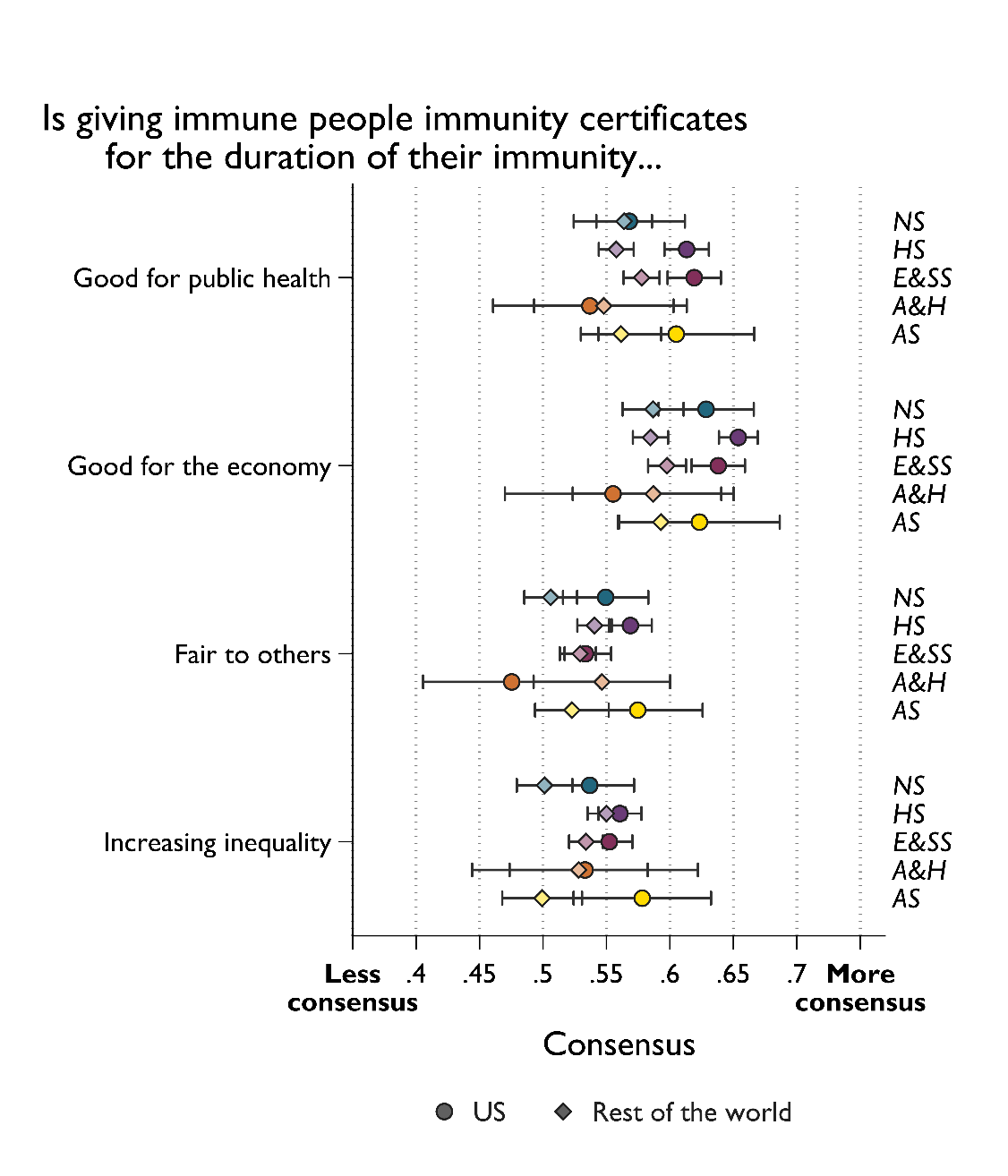


**Supplementary Figure 14.** **Differences in consensus on perceived benefits to public health and economy, fairness, and societal inequality of immunity certificate between US and non-US scientists, by field**. *AS* = Applied Sciences; *A&H* = Arts & Humanities; *E&SS* = Economic & Social Sciences; *HS* = Health Sciences; *NS* = Natural Sciences. Error bars represent 95% confidence intervals obtained from bootstrap resampling with 300 replications. Null responses are excluded from the calculation of consensus.

**
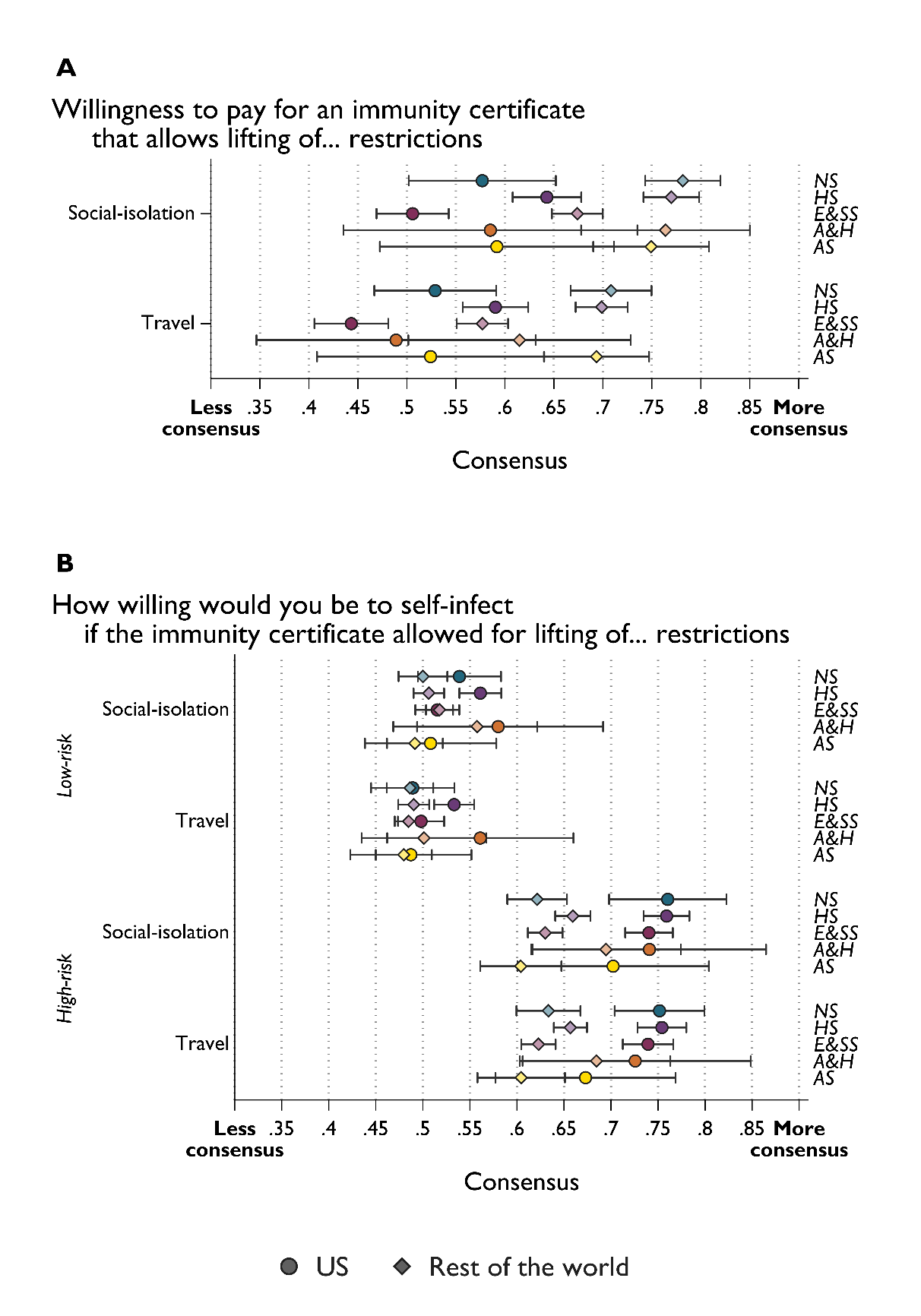
**

**Supplementary Figure 15.** **Differences in consensus on willingness to pay (*A*) and willingness to self-infect (*B*) of immunity certificate between US and non-US scientists, by field**. *AS* = Applied Sciences; *A&H* = Arts & Humanities; *E&SS* = Economic & Social Sciences; *HS* = Health Sciences; *NS* = Natural Sciences. Error bars represent 95% confidence intervals obtained from bootstrap resampling with 300 replications. Null responses are excluded from the calculation of consensus.

**Supplementary Table 9. Differences in the level of consensus between US and non-US scientists by field.**

|  | Non-US | | US | |  | |  |
| --- | --- | --- | --- | --- | --- | --- | --- |
|  | M 1 | SD 1 | M 2 | SD 2 | *p*  (unadj.) | *p*  (adjusted) | |
| Good for public health | | | | | | | |
| *AS* | 0.561 | 0.383 | 0.605 | 0.379 | 0.220 | 1 | |
| *A&H* | 0.548 | 0.362 | 0.537 | 0.361 | 0.820 | 1 | |
| *E&SS* | 0.578 | 0.385 | 0.619 | 0.379 | 0.001 | .007** | |
| *HS* | 0.558 | 0.350 | 0.613 | 0.369 | <.001 | <.001*** | |
| *NS* | 0.564 | 0.365 | 0.568 | 0.426 | 0.860 | 1 | |
| Good for the economy | | | | | | | |
| *AS* | 0.593 | 0.411 | 0.623 | 0.389 | 0.420 | 1 | |
| *A&H* | 0.587 | 0.418 | 0.555 | 0.401 | 0.570 | 1 | |
| *E&SS* | 0.598 | 0.405 | 0.638 | 0.379 | 0.0027 | .014* | |
| *HS* | 0.585 | 0.352 | 0.654 | 0.321 | <.001 | <.001*** | |
| *NS* | 0.587 | 0.399 | 0.629 | 0.363 | 0.079 | 0.4 | |
| Fair to others who do not have immunity | | | | | | | |
| *AS* | 0.523 | 0.35 | 0.575 | 0.315 | 0.100 | 1 | |
| *A&H* | 0.546 | 0.355 | 0.475 | 0.328 | 0.130 | 1 | |
| *E&SS* | 0.529 | 0.335 | 0.534 | 0.366 | 0.720 | 1 | |
| *HS* | 0.541 | 0.342 | 0.569 | 0.353 | 0.0096 | .048* | |
| *NS* | 0.506 | 0.346 | 0.549 | 0.327 | 0.037 | 0.19 | |
| Increasing inequality | | | | | | | |
| *AS* | 0.499 | 0.377 | 0.578 | 0.334 | 0.022 | 0.11 | |
| *A&H* | 0.528 | 0.359 | 0.533 | 0.419 | 0.920 | 1 | |
| *E&SS* | 0.534 | 0.362 | 0.552 | 0.327 | 0.120 | 1 | |
| *HS* | 0.55 | 0.379 | 0.561 | 0.354 | 0.360 | 1 | |
| *NS* | 0.501 | 0.364 | 0.537 | 0.337 | 0.100 | 1 | |
| Willingness to pay: Lifting of social-isolation restrictions | | | | | | | |
| *AS* | 0.749 | 0.709 | 0.592 | 0.734 | 0.018 | .092† | |
| *A&H* | 0.764 | 0.57 | 0.585 | 0.689 | 0.031 | 0.16 | |
| *E&SS* | 0.674 | 0.698 | 0.506 | 0.657 | <.001 | <.001*** | |
| *HS* | 0.77 | 0.717 | 0.643 | 0.729 | <.001 | <.001*** | |
| *NS* | 0.782 | 0.636 | 0.577 | 0.723 | <.001 | <.001*** | |
| Willingness to pay: Lifting of all restrictions and resumption of local and international travel | | | | | | | |
| *AS* | 0.694 | 0.642 | 0.524 | 0.712 | 0.006 | .03* | |
| *A&H* | 0.615 | 0.748 | 0.489 | 0.646 | 0.200 | 1 | |
| *E&SS* | 0.577 | 0.704 | 0.443 | 0.671 | <.001 | <.001*** | |
| *HS* | 0.699 | 0.67 | 0.59 | 0.695 | <.001 | <.001*** | |
| *NS* | 0.708 | 0.682 | 0.529 | 0.6 | <.001 | <.001*** | |
| Willingness to self-infect: Lifting of social-isolation restrictions (*Low-risk* scenario) | | | | | | | |
| *AS* | 0.492 | 0.352 | 0.508 | 0.421 | 0.630 | 1 | |
| *A&H* | 0.558 | 0.417 | 0.58 | 0.512 | 0.710 | 1 | |
| *E&SS* | 0.518 | 0.383 | 0.515 | 0.414 | 0.860 | 1 | |
| *HS* | 0.506 | 0.406 | 0.561 | 0.463 | <.001 | <.001*** | |
| *NS* | 0.500 | 0.424 | 0.539 | 0.426 | 0.140 | 1 | |
| Willingness to self-infect: Lifting of all restrictions and resumption of local and international travel (*Low-risk* scenario) | | | | | | | |
| *AS* | 0.480 | 0.352 | 0.487 | 0.386 | 0.830 | 1 | |
| *A&H* | 0.501 | 0.434 | 0.561 | 0.455 | 0.320 | 1 | |
| *E&SS* | 0.485 | 0.39 | 0.498 | 0.433 | 0.340 | 1 | |
| *HS* | 0.490 | 0.409 | 0.533 | 0.441 | 0.0015 | .0074** | |
| *NS* | 0.486 | 0.403 | 0.489 | 0.424 | 0.910 | 1 | |
| Willingness to self-infect: Lifting of social-isolation restrictions (*High-risk* scenario) | | | | | | | |
| *AS* | 0.604 | 0.510 | 0.702 | 0.607 | 0.054 | 0.27 | |
| *A&H* | 0.695 | 0.518 | 0.741 | 0.570 | 0.530 | 1 | |
| *E&SS* | 0.630 | 0.49 | 0.740 | 0.449 | <.001 | <.001*** | |
| *HS* | 0.659 | 0.469 | 0.759 | 0.505 | <.001 | <.001*** | |
| *NS* | 0.622 | 0.518 | 0.76 | 0.601 | <.001 | <.001*** | |
| Willingness to self-infect: Lifting of all restrictions and resumption of local and international travel (*High-risk* scenario) | | | | | | | |
| *AS* | 0.605 | 0.548 | 0.673 | 0.572 | 0.200 | 1 | |
| *A&H* | 0.685 | 0.508 | 0.726 | 0.564 | 0.570 | 1 | |
| *E&SS* | 0.623 | 0.483 | 0.739 | 0.475 | <.001 | <.001*** | |
| *HS* | 0.657 | 0.441 | 0.754 | 0.539 | <.001 | <.001*** | |
| *NS* | 0.633 | 0.554 | 0.752 | 0.460 | <.001 | .0016** | |

*Notes*. *AS* = Applied Sciences; *A&H* = Arts & Humanities; *E&SS* = Economic & Social Sciences; *HS* = Health Sciences; *NS* = Natural Sciences. Two-tailed *t-*test. Significance levels: ****p* < 0.001, ***p* < 0.01, **p* < 0.05, †*p* < 0.1 based on p-values with Bonferroni correction for multiple-comparison. Standard deviations are computed using bootstrap resampling with 300 replications.

# Regressions-based results

**Supplementary Table 10. Ordered logit regression for attitudes toward immunity certificates.**

| Dependent variable | Good for  public health | Good for  the economy | Fair to others | Increasing  inequality |
| --- | --- | --- | --- | --- |
| Scientific fields |  |  |  |  |
| *Applied Sciences* | 1.075 | 1.061 | 1.005 | 0.998 |
|  | (0.0980) | (0.0980) | (0.0926) | (0.0926) |
| *Arts & Humanities* | 0.897 | 0.988 | 1.139 | 1.037 |
|  | (0.128) | (0.145) | (0.165) | (0.152) |
| *Health Sciences* | 0.918^†^ | 0.938 | 0.939 | 0.984 |
|  | (0.0454) | (0.0460) | (0.0468) | (0.0487) |
| *Natural Sciences* | 0.983 | 0.990 | 1.013 | 0.997 |
|  | (0.0681) | (0.0700) | (0.0692) | (0.0703) |
| Age |  |  |  |  |
| *18–29* | 1.239^*^ | 1.310^**^ | 1.140 | 0.973 |
|  | (0.119) | (0.135) | (0.115) | (0.0975) |
| *40–49* | 0.921 | 0.882^*^ | 0.945 | 0.939 |
|  | (0.0544) | (0.0521) | (0.0546) | (0.0543) |
| *50–59* | 1.058 | 0.847^*^ | 1.157^*^ | 0.829^*^ |
|  | (0.0774) | (0.0621) | (0.0841) | (0.0617) |
| *60–69* | 0.979 | 0.787^**^ | 1.226^*^ | 0.731^***^ |
|  | (0.0841) | (0.0679) | (0.105) | (0.0638) |
| *70+* | 0.927 | 0.808^*^ | 1.389^**^ | 0.582^***^ |
|  | (0.0967) | (0.0856) | (0.140) | (0.0587) |
| Gender |  |  |  |  |
| *Female* | 0.837^***^ | 0.863^***^ | 0.774^***^ | 1.254^***^ |
|  | (0.0368) | (0.0379) | (0.0341) | (0.0555) |
| *Other* | 0.382^*^ | 0.263^***^ | 0.607 | 3.553^*^ |
|  | (0.187) | (0.106) | (0.357) | (2.158) |
| *Prefer not to say* | 0.763 | 0.733 | 0.696 | 1.392 |
|  | (0.258) | (0.237) | (0.239) | (0.472) |
| Professorship |  |  |  |  |
| *Associate Professor*  *(or equivalent)* | 1.017 | 1.073 | 1.003 | 0.931 |
|  | (0.0627) | (0.0657) | (0.0612) | (0.0575) |
| *Full Professor (or above)* | 1.057 | 1.149^*^ | 1.091 | 0.830^**^ |
|  | (0.0665) | (0.0720) | (0.0673) | (0.0519) |
| Time to normality |  |  |  |  |
| *Fewer than 1 month* | 0.318^**^ | 0.352^*^ | 0.452^†^ | 0.593 |
|  | (0.116) | (0.143) | (0.187) | (0.243) |
| *1-3 months* | 0.778^†^ | 0.772^†^ | 0.897 | 1.266^†^ |
|  | (0.103) | (0.111) | (0.125) | (0.168) |
| *7-12 months* | 1.214^**^ | 1.183^*^ | 1.024 | 1.012 |
|  | (0.0820) | (0.0834) | (0.0697) | (0.0685) |
| *More than 12 months* | 1.370^***^ | 1.362^***^ | 1.070 | 1.069 |
|  | (0.0903) | (0.0929) | (0.0712) | (0.0700) |
| Political views (liberal-conservative, 7-point scale) | 1.044^*^ | 1.038^†^ | 1.130^***^ | 0.893^***^ |
|  | (0.0196) | (0.0198) | (0.0212) | (0.0172) |
| Religious (dummy) | 0.895^*^ | 0.919^†^ | 0.929^†^ | 1.066 |
|  | (0.0394) | (0.0407) | (0.0407) | (0.0475) |
| Marital status |  |  |  |  |
| *Married* | 1.090 | 1.216^**^ | 1.055 | 0.939 |
|  | (0.0777) | (0.0886) | (0.0765) | (0.0694) |
| *De facto partnered* | 0.978 | 1.049 | 1.011 | 1.033 |
|  | (0.0750) | (0.0813) | (0.0783) | (0.0831) |
| *Divorced* | 1.088 | 1.000 | 1.016 | 0.874 |
|  | (0.138) | (0.124) | (0.127) | (0.111) |
| *Separated* | 1.385 | 1.312 | 1.018 | 1.021 |
|  | (0.302) | (0.259) | (0.190) | (0.183) |
| *Widowed* | 0.960 | 1.002 | 1.084 | 0.904 |
|  | (0.222) | (0.258) | (0.253) | (0.199) |
| Have offspring (dummy) | 0.962 | 1.043 | 1.002 | 1.055 |
|  | (0.0526) | (0.0571) | (0.0554) | (0.0581) |
| *ln*(# confirmed cases+1) | 0.920 | 0.939 | 1.077 | 1.213 |
|  | (0.225) | (0.245) | (0.250) | (0.280) |
| Case fatality rate (CFR, 0–100) | 0.798^†^ | 0.769^*^ | 0.889 | 1.183 |
|  | (0.0933) | (0.0887) | (0.101) | (0.137) |
| Stringency Index (0–100) | 1.002 | 0.998 | 0.998 | 1.002 |
|  | (0.00667) | (0.00656) | (0.00640) | (0.00688) |
| *RePEc* | 1.052 | 1.132 | 1.117 | 0.756^***^ |
|  | (0.0924) | (0.101) | (0.0942) | (0.0618) |
| Country fixed-effects | Yes | Yes | Yes | Yes |
| Time fixed-effects | Yes | Yes | Yes | Yes |
| N | 7898 | 7885 | 7882 | 7856 |
| Pseudo *R^2^* | 0.015 | 0.015 | 0.018 | 0.020 |

*Notes.* Odd ratios from ordered logistic regression. Standard errors (robust) in parentheses. † *p* < .10; * *p* < .05; ** *p* < .01; *** *p* < .001. Reference category: *Economic & Social Sciences*, *Age (30-39), Gender (Male), Assistant Professor (or below), Time to normality (3-6 months), Never attend religious services, Single,* and *No children*.

**Supplementary Table 11. Ordered logit regression for willingness to pay and self-infect for immunity certificates.**

|  | **Willingness-to-pay** | | **Willingness to self-infect** | | | |
| --- | --- | --- | --- | --- | --- | --- |
|  | Lifting  social-isolation  restrictions | Lifting  travel  restrictions | *Low-risk* | | *High-risk* | |
| Dependent variable |  |  | Social-  isolation | Travel | Social-  isolation | Travel |
| Scientific fields |  |  |  |  |  |  |
| *Applied Sciences* | 0.770^**^ | 0.755^**^ | 0.847^†^ | 0.845^†^ | 0.907 | 0.770^**^ |
|  | (0.0734) | (0.0694) | (0.0780) | (0.0764) | (0.0996) | (0.0734) |
| *Arts & Humanities* | 0.860 | 0.889 | 0.968 | 1.105 | 1.062 | 0.860 |
|  | (0.123) | (0.128) | (0.141) | (0.155) | (0.182) | (0.123) |
| *Health Sciences* | 0.779^***^ | 0.767^***^ | 1.016 | 0.961 | 0.984 | 0.779^***^ |
|  | (0.0424) | (0.0406) | (0.0527) | (0.0497) | (0.0605) | (0.0424) |
| *Natural Sciences* | 0.697^***^ | 0.687^***^ | 0.924 | 0.931 | 0.912 | 0.697^***^ |
|  | (0.0525) | (0.0490) | (0.0656) | (0.0657) | (0.0754) | (0.0525) |
| Age |  |  |  |  |  |  |
| *18–29* | 1.062 | 0.940 | 1.210^†^ | 1.114 | 1.080 | 1.109 |
|  | (0.110) | (0.0911) | (0.120) | (0.110) | (0.132) | (0.131) |
| *40–49* | 0.776^***^ | 0.771^***^ | 0.813^***^ | 0.779^***^ | 0.861^*^ | 0.855^*^ |
|  | (0.0500) | (0.0482) | (0.0505) | (0.0480) | (0.0638) | (0.0632) |
| *50–59* | 0.769^***^ | 0.770^***^ | 0.760^***^ | 0.794^**^ | 0.857^†^ | 0.941 |
|  | (0.0612) | (0.0598) | (0.0580) | (0.0597) | (0.0777) | (0.0844) |
| *60–69* | 0.826^*^ | 0.772^**^ | 0.877 | 0.858^†^ | 0.945 | 0.936 |
|  | (0.0783) | (0.0716) | (0.0780) | (0.0757) | (0.100) | (0.0985) |
| *70+* | 0.740^*^ | 0.669^***^ | 0.970 | 0.960 | 1.186 | 1.227 |
|  | (0.0896) | (0.0764) | (0.102) | (0.101) | (0.146) | (0.154) |
| Gender |  |  |  |  |  |  |
| *Female* | 0.642^***^ | 0.664^***^ | 0.788^***^ | 0.767^***^ | 0.838^**^ | 0.811^***^ |
|  | (0.0315) | (0.0313) | (0.0364) | (0.0356) | (0.0464) | (0.0447) |
| *Other* | 0.219^*^ | 0.242^*^ | 0.478 | 0.737 | 0.980 | 1.147 |
|  | (0.134) | (0.137) | (0.281) | (0.310) | (0.698) | (0.661) |
| *Prefer not to say* | 0.684 | 0.970 | 1.153 | 1.322 | 1.372 | 1.480 |
|  | (0.250) | (0.374) | (0.492) | (0.506) | (0.571) | (0.579) |
| Professorship |  |  |  |  |  |  |
| *Associate Professor*  *(or equivalent)* | 1.161^*^ | 1.174^*^ | 1.059 | 1.100 | 1.041 | 1.000 |
|  | (0.0764) | (0.0743) | (0.0678) | (0.0686) | (0.0793) | (0.0755) |
| *Full Professor (or above)* | 1.716^***^ | 1.723^***^ | 1.316^***^ | 1.362^***^ | 1.268^**^ | 1.227^**^ |
|  | (0.119) | (0.114) | (0.0857) | (0.0887) | (0.0974) | (0.0939) |
| Time to normality |  |  |  |  |  |  |
| *Fewer than 1 month* | 0.371^**^ | 0.415^**^ | 0.719 | 0.811 | 1.428 | 1.159 |
|  | (0.120) | (0.122) | (0.229) | (0.248) | (0.500) | (0.373) |
| *1-3 months* | 0.596^***^ | 0.671^**^ | 1.141 | 1.134 | 1.336^*^ | 1.267^†^ |
|  | (0.0823) | (0.0839) | (0.147) | (0.140) | (0.185) | (0.175) |
| *7-12 months* | 1.102 | 1.104 | 0.884^†^ | 0.855^*^ | 0.760^***^ | 0.702^***^ |
|  | (0.0784) | (0.0761) | (0.0625) | (0.0601) | (0.0622) | (0.0573) |
| *More than 12 months* | 1.222^**^ | 1.241^**^ | 0.783^***^ | 0.756^***^ | 0.635^***^ | 0.580^***^ |
|  | (0.0848) | (0.0833) | (0.0539) | (0.0517) | (0.0504) | (0.0460) |
| Political views (liberal-conservative, 7-point scale) | 1.073^***^ | 1.071^***^ | 1.156^***^ | 1.156^***^ | 1.206^***^ | 1.178^***^ |
|  | (0.0197) | (0.0188) | (0.0216) | (0.0212) | (0.0252) | (0.0246) |
| Religious (dummy) | 1.010 | 1.045 | 1.108^*^ | 1.073 | 1.207^***^ | 1.145^*^ |
|  | (0.0491) | (0.0489) | (0.0509) | (0.0494) | (0.0660) | (0.0633) |
| Marital status |  |  |  |  |  |  |
| *Married* | 1.104 | 1.113 | 0.768^***^ | 0.754^***^ | 0.652^***^ | 0.697^***^ |
|  | (0.0870) | (0.0841) | (0.0588) | (0.0588) | (0.0591) | (0.0630) |
| *De facto partnered* | 1.039 | 0.989 | 0.864^†^ | 0.840^*^ | 0.713^***^ | 0.723^***^ |
|  | (0.0879) | (0.0798) | (0.0711) | (0.0701) | (0.0693) | (0.0703) |
| *Divorced* | 1.127 | 1.062 | 0.945 | 0.886 | 0.885 | 0.874 |
|  | (0.150) | (0.136) | (0.123) | (0.118) | (0.130) | (0.132) |
| *Separated* | 1.190 | 1.187 | 1.142 | 1.164 | 0.829 | 1.010 |
|  | (0.247) | (0.245) | (0.235) | (0.248) | (0.192) | (0.233) |
| *Widowed* | 1.268 | 1.081 | 0.705 | 0.720 | 0.847 | 1.124 |
|  | (0.376) | (0.322) | (0.186) | (0.172) | (0.267) | (0.342) |
| Have offspring (dummy) | 1.071 | 0.937 | 1.113^†^ | 1.040 | 1.194^*^ | 1.119 |
|  | (0.0647) | (0.0554) | (0.0633) | (0.0594) | (0.0856) | (0.0784) |
| *ln*(Confirmed cases+1) | 0.993 | 1.072 | 0.839 | 1.087 | 1.090 | 1.096 |
|  | (0.223) | (0.234) | (0.198) | (0.243) | (0.277) | (0.275) |
| Case fatality rate (CFR, 0–100) | 0.823^†^ | 0.939 | 0.834 | 0.836 | 1.005 | 0.967 |
|  | (0.0924) | (0.106) | (0.0975) | (0.0962) | (0.136) | (0.133) |
| Stringency Index (0–100) | 0.997 | 0.999 | 1.001 | 0.996 | 1.001 | 1.002 |
|  | (0.00663) | (0.00656) | (0.00726) | (0.00708) | (0.00793) | (0.00811) |
| *RePEc* | 1.524^***^ | 1.525^***^ | 1.032 | 1.027 | 0.949 | 1.006 |
|  | (0.131) | (0.129) | (0.0859) | (0.0872) | (0.0979) | (0.102) |
| Country fixed-effects | Yes | Yes | Yes | Yes | Yes | Yes |
| Time fixed-effects | Yes | Yes | Yes | Yes | Yes | Yes |
| N | 7793 | 7786 | 7770 | 7709 | 7763 | 7734 |
| Pseudo *R^2^* | 7793 | 7786 | 7770 | 7709 | 7763 | 7734 |

*Notes.* Odd ratios from ordered logistic regression. Standard errors (robust) in parentheses. † *p* < .10; * *p* < .05; ** *p* < .01; *** *p* < .001. Reference category: *Economic & Social Sciences*, *Age (30-39), Gender (Male), Assistant Professor (or below), Time to normality (3-6 months), Never attend religious services, Single,* and *No children*. For the two variables on willingness to self-infect in high-risk scenario, we merge the two highest responses into a single group to avoid non-convergence issue in the maximum likelihood estimation.

Below we report the ordered logistic regression results without controlling for political views, religiosity, marital status, and offspring dummy from Supplementary Table 10 and 11.

**Supplementary Table 12. Robustness checks - attitudes toward immunity certificates.**

| Dependent variable | Good for  public health | Good for  the economy | Fair to others | Increasing  inequality |
| --- | --- | --- | --- | --- |
| Scientific fields |  |  |  |  |
| *Applied Sciences* | 1.063 | 1.076 | 1.028 | 0.970 |
|  | (0.0828) | (0.0825) | (0.0792) | (0.0776) |
| *Arts & Humanities* | 0.788^†^ | 0.892 | 1.011 | 1.054 |
|  | (0.0966) | (0.107) | (0.120) | (0.133) |
| *Health Sciences* | 0.899^*^ | 0.924^†^ | 0.910^*^ | 0.993 |
|  | (0.0382) | (0.0396) | (0.0390) | (0.0421) |
| *Natural Sciences* | 0.962 | 0.986 | 0.933 | 1.013 |
|  | (0.0569) | (0.0592) | (0.0555) | (0.0610) |
| Age |  |  |  |  |
| *18–29* | 1.200^*^ | 1.154 | 1.075 | 0.939 |
|  | (0.101) | (0.102) | (0.0919) | (0.0803) |
| *40–49* | 0.968 | 0.950 | 1.007 | 0.896^*^ |
|  | (0.0476) | (0.0465) | (0.0488) | (0.0431) |
| *50–59* | 1.065 | 0.917 | 1.211^**^ | 0.802^***^ |
|  | (0.0645) | (0.0556) | (0.0726) | (0.0495) |
| *60–69* | 1.016 | 0.865^*^ | 1.247^**^ | 0.731^***^ |
|  | (0.0732) | (0.0628) | (0.0904) | (0.0530) |
| *70+* | 0.947 | 0.889 | 1.427^***^ | 0.582^***^ |
|  | (0.0850) | (0.0809) | (0.125) | (0.0509) |
| Gender |  |  |  |  |
| *Female* | 0.852^***^ | 0.856^***^ | 0.765^***^ | 1.270^***^ |
|  | (0.0318) | (0.0319) | (0.0285) | (0.0481) |
| *Other* | 0.515^†^ | 0.373^**^ | 0.637 | 2.424^†^ |
|  | (0.193) | (0.130) | (0.255) | (1.140) |
| *Prefer not to say* | 0.582^*^ | 0.511^**^ | 0.702 | 1.425 |
|  | (0.142) | (0.132) | (0.191) | (0.374) |
| Professorship |  |  |  |  |
| *Associate Professor*  *(or equivalent)* | 0.973 | 1.044 | 0.980 | 0.947 |
|  | (0.0505) | (0.0540) | (0.0507) | (0.0494) |
| *Full Professor (or above)* | 1.049 | 1.140^*^ | 1.105^†^ | 0.842^**^ |
|  | (0.0561) | (0.0612) | (0.0581) | (0.0448) |
| Time to normality |  |  |  |  |
| *Fewer than 1 month* | 0.298^***^ | 0.353^***^ | 0.530^*^ | 0.724 |
|  | (0.0853) | (0.110) | (0.159) | (0.227) |
| *1-3 months* | 0.805^*^ | 0.801^†^ | 0.907 | 1.154 |
|  | (0.0884) | (0.0955) | (0.104) | (0.124) |
| *7-12 months* | 1.221^***^ | 1.176^**^ | 1.027 | 1.020 |
|  | (0.0711) | (0.0705) | (0.0602) | (0.0592) |
| *More than 12 months* | 1.345^***^ | 1.340^***^ | 1.027 | 1.100^†^ |
|  | (0.0759) | (0.0777) | (0.0583) | (0.0617) |
| *ln*(# confirmed cases+1) | 0.982 | 1.029 | 1.219 | 1.142 |
|  | (0.197) | (0.219) | (0.236) | (0.221) |
| Case fatality rate (CFR, 0–100) | 0.951 | 0.948 | 1.008 | 1.026 |
|  | (0.156) | (0.149) | (0.111) | (0.0632) |
| Stringency Index (0–100) | 1.005 | 1.004 | 1.001 | 1.002 |
|  | (0.00548) | (0.00547) | (0.00538) | (0.00567) |
| *RePEc* | 1.085 | 1.151^†^ | 1.138^†^ | 0.752^***^ |
|  | (0.0799) | (0.0868) | (0.0840) | (0.0534) |
| Country fixed-effects | Yes | Yes | Yes | Yes |
| Time fixed-effects | Yes | Yes | Yes | Yes |
| N | 10438 | 10412 | 10407 | 10367 |
| Pseudo *R^2^* | 0.013 | 0.013 | 0.015 | 0.015 |

*Notes.* Odd ratios from ordered logistic regression. Standard errors (robust) in parentheses. † *p* < .10; * *p* < .05; ** *p* < .01; *** *p* < .001. Reference category: *Economic & Social Sciences*, *Age (30-39), Gender (Male), Assistant Professor (or below),* and *Time to normality (3-6 months)*.

**Supplementary Table 13. Robustness checks - willingness to pay and self-infect for immunity certificates.**

|  | **Willingness-to-pay** | | **Willingness to self-infect** | | | |
| --- | --- | --- | --- | --- | --- | --- |
|  | Lifting  social-isolation  restrictions | Lifting  travel  restrictions | *Low-risk* | | *High-risk* | |
| Dependent variable |  |  | Social-  isolation | Travel | Social-  isolation | Travel |
| Scientific fields |  |  |  |  |  |  |
| *Applied Sciences* | 0.754^***^ | 0.753^***^ | 0.953 | 0.938 | 0.970 | 0.892 |
|  | (0.0630) | (0.0604) | (0.0760) | (0.0747) | (0.0897) | (0.0846) |
| *Arts & Humanities* | 0.853 | 0.855 | 0.897 | 1.013 | 0.890 | 0.869 |
|  | (0.106) | (0.110) | (0.109) | (0.121) | (0.131) | (0.131) |
| *Health Sciences* | 0.773^***^ | 0.756^***^ | 1.021 | 0.975 | 0.970 | 0.950 |
|  | (0.0368) | (0.0351) | (0.0460) | (0.0438) | (0.0513) | (0.0502) |
| *Natural Sciences* | 0.688^***^ | 0.692^***^ | 0.928 | 0.946 | 0.897 | 0.877^†^ |
|  | (0.0450) | (0.0424) | (0.0566) | (0.0575) | (0.0641) | (0.0619) |
| Age |  |  |  |  |  |  |
| *18–29* | 1.005 | 0.917 | 1.310^**^ | 1.254^**^ | 1.187^†^ | 1.180^†^ |
|  | (0.0907) | (0.0774) | (0.112) | (0.107) | (0.121) | (0.118) |
| *40–49* | 0.844^**^ | 0.796^***^ | 0.810^***^ | 0.778^***^ | 0.872^*^ | 0.849^**^ |
|  | (0.0460) | (0.0417) | (0.0420) | (0.0404) | (0.0538) | (0.0524) |
| *50–59* | 0.863^*^ | 0.844^**^ | 0.816^**^ | 0.839^**^ | 0.960 | 1.004 |
|  | (0.0577) | (0.0548) | (0.0522) | (0.0537) | (0.0719) | (0.0748) |
| *60–69* | 0.885 | 0.795^**^ | 0.916 | 0.894 | 1.013 | 0.970 |
|  | (0.0719) | (0.0631) | (0.0693) | (0.0676) | (0.0905) | (0.0862) |
| *70+* | 0.865 | 0.723^**^ | 1.009 | 0.940 | 1.318^**^ | 1.303^*^ |
|  | (0.0909) | (0.0713) | (0.0923) | (0.0863) | (0.138) | (0.138) |
| Gender |  |  |  |  |  |  |
| *Female* | 0.655^***^ | 0.660^***^ | 0.770^***^ | 0.747^***^ | 0.780^***^ | 0.755^***^ |
|  | (0.0276) | (0.0268) | (0.0307) | (0.0299) | (0.0367) | (0.0354) |
| *Other* | 0.172^**^ | 0.244^**^ | 0.483 | 0.668 | 0.875 | 1.068 |
|  | (0.103) | (0.117) | (0.233) | (0.250) | (0.438) | (0.479) |
| *Prefer not to say* | 0.499^**^ | 0.680 | 0.878 | 1.094 | 1.074 | 1.172 |
|  | (0.130) | (0.191) | (0.225) | (0.286) | (0.322) | (0.347) |
| Professorship |  |  |  |  |  |  |
| *Associate Professor*  *(or equivalent)* | 1.137^*^ | 1.115^*^ | 1.066 | 1.067 | 1.039 | 1.011 |
|  | (0.0648) | (0.0609) | (0.0585) | (0.0582) | (0.0675) | (0.0656) |
| *Full Professor (or above)* | 1.706^***^ | 1.656^***^ | 1.239^***^ | 1.252^***^ | 1.159^*^ | 1.137^†^ |
|  | (0.102) | (0.0954) | (0.0698) | (0.0706) | (0.0767) | (0.0749) |
| Time to normality |  |  |  |  |  |  |
| *Fewer than 1 month* | 0.382^**^ | 0.507^**^ | 0.900 | 1.115 | 1.476 | 1.384 |
|  | (0.116) | (0.128) | (0.250) | (0.296) | (0.435) | (0.378) |
| *1-3 months* | 0.704^**^ | 0.742^**^ | 1.178 | 1.195 | 1.428^**^ | 1.341^*^ |
|  | (0.0838) | (0.0806) | (0.132) | (0.129) | (0.172) | (0.163) |
| *7-12 months* | 1.113^†^ | 1.112^†^ | 0.851^**^ | 0.852^**^ | 0.717^***^ | 0.693^***^ |
|  | (0.0682) | (0.0665) | (0.0523) | (0.0522) | (0.0503) | (0.0485) |
| *More than 12 months* | 1.235^***^ | 1.217^***^ | 0.732^***^ | 0.735^***^ | 0.589^***^ | 0.560^***^ |
|  | (0.0732) | (0.0705) | (0.0438) | (0.0438) | (0.0399) | (0.0379) |
| *ln*(Confirmed cases+1) | 1.058 | 1.023 | 0.942 | 1.078 | 1.133 | 1.123 |
|  | (0.208) | (0.194) | (0.189) | (0.204) | (0.241) | (0.232) |
| Case fatality rate (CFR, 0–100) | 1.054 | 1.134^†^ | 1.038 | 1.041 | 1.073 | 1.063 |
|  | (0.0653) | (0.0857) | (0.0648) | (0.0645) | (0.0897) | (0.0856) |
| Stringency Index (0–100) | 1.001 | 1.001 | 1.002 | 0.998 | 1.000 | 1.001 |
|  | (0.00564) | (0.00544) | (0.00586) | (0.00573) | (0.00672) | (0.00680) |
| *RePEc* | 1.475^***^ | 1.464^***^ | 1.031 | 1.049 | 0.938 | 0.955 |
|  | (0.111) | (0.108) | (0.0753) | (0.0776) | (0.0834) | (0.0838) |
| Country fixed-effects | Yes | Yes | Yes | Yes | Yes | Yes |
| Time fixed-effects | Yes | Yes | Yes | Yes | Yes | Yes |
| N | 10219 | 10206 | 10105 | 10010 | 10057 | 10017 |
| Pseudo *R^2^* | 0.032 | 0.026 | 0.018 | 0.017 | 0.042 | 0.042 |

*Notes.* Odd ratios from ordered logistic regression. Standard errors (robust) in parentheses. † *p* < .10; * *p* < .05; ** *p* < .01; *** *p* < .001. Reference category: *Economic & Social Sciences*, *Age (30-39), Gender (Male), Assistant Professor (or below),* and *Time to normality (3-6 months)*. For the two variables on willingness to self-infect in high-risk scenario, we merge the two highest responses into a single group to avoid non-convergence issue in the maximum likelihood estimation.

# Return-to-normality timeline

When asking scientists about the expected timelines within which they believe the current policy measures could bring back normality *without* the use of immunity certificates, more than half (52%) of the respondents think the pandemic will last longer than 12 months (Supplementary Figure 11). Health Scientists were far more convinced that it will take a longer time to get back to normality (Supplementary Table 6). A nonparametric pairwise comparison shows Health Scientists’ opinions differ from Applied Scientists (*d* = .122, *p* < .001), Natural Scientists (*d* = .121, *p* < .001), and Economists and Social Scientists (*d* = .116, *p* < .001). US-based scientists are also less optimistic about the time it will take for life to return to normal (*d* = .301, *p* < .001) and this difference is evident across all fields (Supplementary Table 7). Scientists who expected the return to normality to last longer were more likely to have more favorable views on all aspects related to immunity certificates compared to those who gave more optimistic estimates regarding the return to some form of normality (Supplementary Figure 12). We found that the average favorability of immunity certificates with respect to public health and the economy increases with estimated time-to-normality – as does the willingness-to-pay. Interestingly, willingness to self-infect to receive immunity certificates decreases with the back-to-normal timeline projections. There is no apparent association between the timeline estimates to fairness and inequality concerns of immunity certificates.


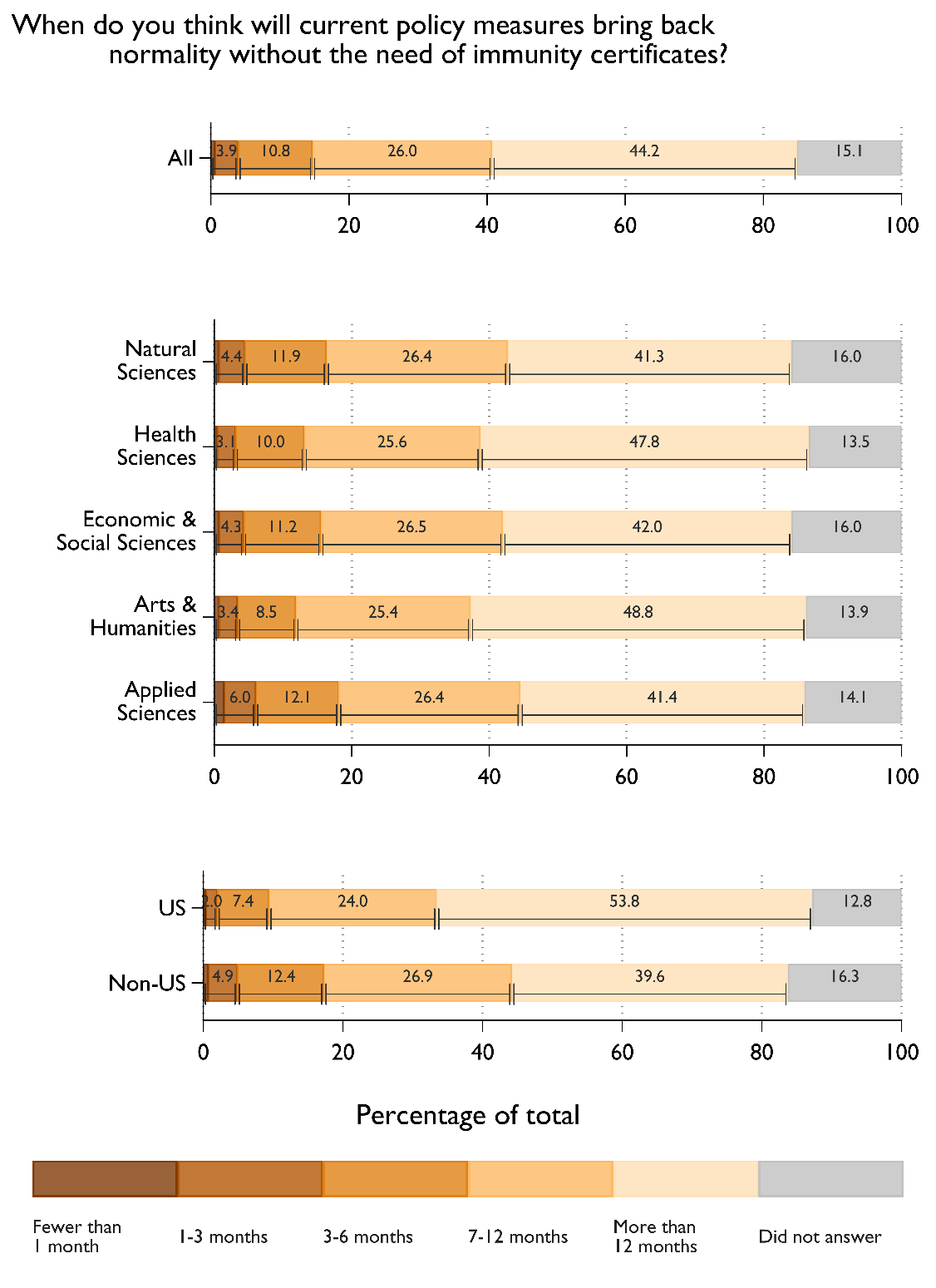


**Supplementary Figure 16.** **Time to normality projections**. *N* = 10,809 non-missing responses. Distributional difference by fields: Kruskal-Wallis equality-of-populations rank test (tie-corrected *χ^2^*(4) = 45.56; *p* < .001). Difference between US and Non-US: Cohen’s *d* = -.301; *z* = -15.66; *p* < .001.

**Supplementary Table 14. Differences in expected back-to-normality timelines across fields.**

| *Expected ‘back-to-normal’ timeline without the need for immunity certificates?* | | | | | |
| --- | --- | --- | --- | --- | --- |
|  |  |  | *d* | z-stat. | *p*-val. |
| Applied Sciences | vs. | Arts & Humanities | -.177 | -2.801** | .006 |
| Applied Sciences | vs. | Economic & Social Sciences | -.041 | -1.419 | .111 |
| Arts & Humanities | vs. | Economic & Social Sciences | .068 | 2.276* | .019 |
| Applied Sciences | vs. | Health Sciences | -.122 | -4.264*** | <.001 |
| Arts & Humanities | vs. | Health Sciences | .015 | .494 | .311 |
| Economic & Social Sciences | vs. | Health Sciences | -.116 | -5.254*** | <.001 |
| Applied Sciences | vs. | Natural Sciences | -.034 | -.772 | .275 |
| Arts & Humanities | vs. | Natural Sciences | .12 | 2.487* | .013 |
| Economic & Social Sciences | vs. | Natural Sciences | .019 | .724 | .261 |
| Health Sciences | vs. | Natural Sciences | .121 | 4.495*** | <.001 |
| *N* |  |  | 10,809 | | |

*Notes*. Cohen’s *d* $\text{= 2*}\text{z}\text{/}\sqrt{n}$. Significance levels: ****p* < .001, ***p* < .01, **p* < .05, †*p* < .1. Non-parametric pairwise multiple comparison (Dunn, 1964) controlling for the false discovery rate using the Benjamini-Hochberg stepwise adjustments.

**Supplementary Table 15. Differences in expected back-to-normality timelines between US and Non-US scholars, by field.**

| *Non-US* vs. *US* | *d* | *z*-stat. | *p-*val. |
| --- | --- | --- | --- |
| *Applied Sciences* | -.229 | -3.055** | .00225 |
| *Arts & Humanities* | -.47 | -3.744*** | <.001 |
| *Economic & Social Sciences* | -.314 | -10.069*** | <.001 |
| *Health Sciences* | -.303 | -9.811*** | <.001 |
| *Natural Sciences* | -.163 | -3.093** | .00198 |

*Notes*. Wilcoxon rank sum test (two-tailed). Cohen’s *d* $\text{= 2*}\text{z}\text{/}\sqrt{n}$. Significance levels: ****p* < .001, ***p* < .01, **p* < .05, †*p* < .1.

**
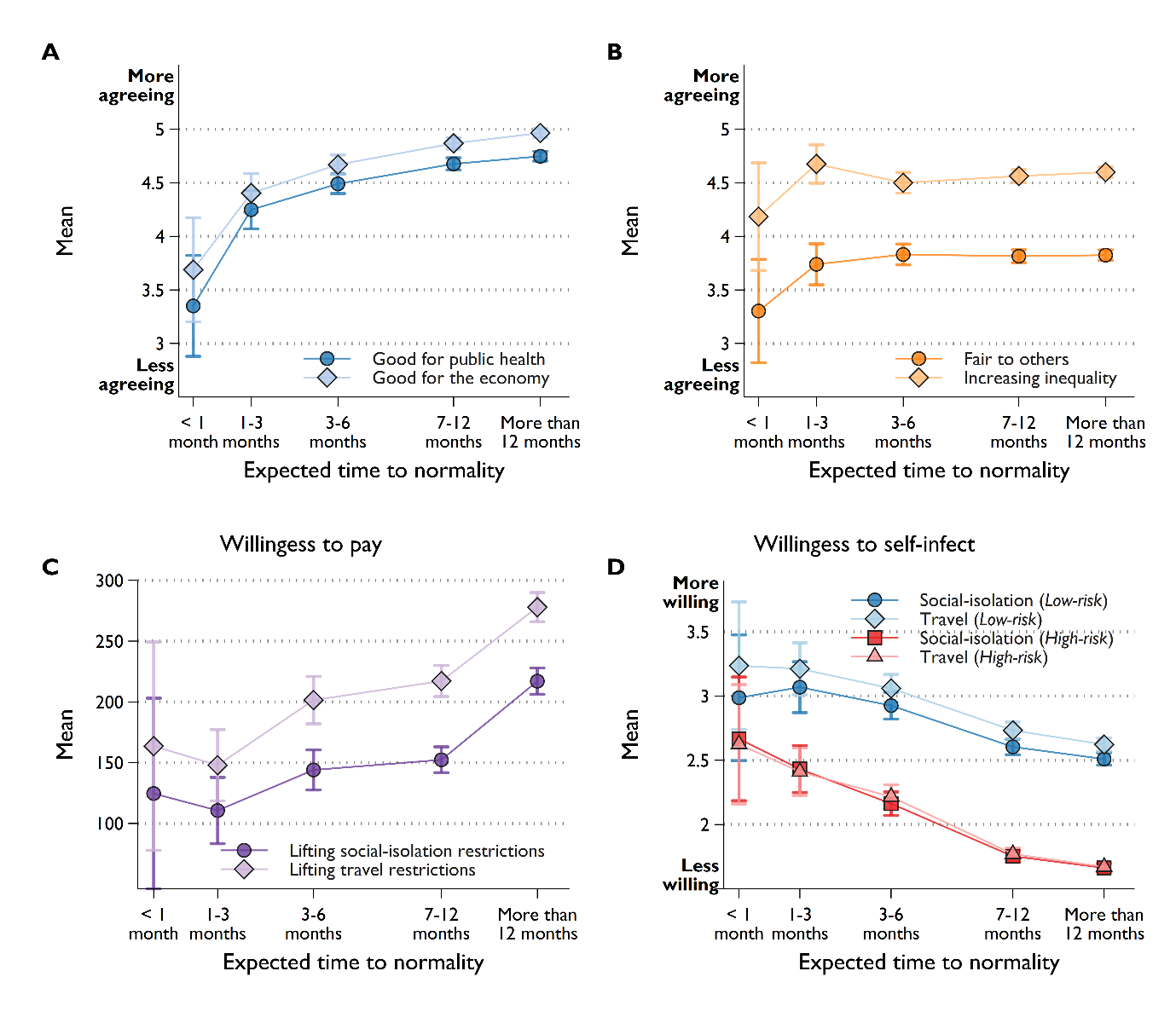
**

**Supplementary Figure 17.** **Time-to-normality and attitude toward immunity certificate**. “*Is giving immune people immunity certificates for the duration of their immunity...”* regarding *good for public health*, *good for the economy* (*A*), *fair to others who do not have immunity,* and *increase inequality* (*B*). Willingness to self-infect for immunity certificate lifting *social-isolation* and *travel* restrictions, if medically assessed as *low-risk* or *high-risk* groups (*C*). Willingness to pay for immunity certificate lifting *social-isolation* and *travel* restrictions (*D*). Responses were averaged according to the answer given to the question “*When do you think will current policy measures (including social distancing and investment in medical research) bring back normality without the need of immunity certificates”*. Error bars represent 95% confidence intervals.

# Response rate

**Supplementary Table 16. Survey response rate, by scientific areas**

| Scientific areas | # Email sent | # Email opened | # Responses | % per opened | % sent |
| --- | --- | --- | --- | --- | --- |
| *Arts and Humanities* | 11,819 | 5,316 | 888 | 16.70% | 7.51% |
| *Business, Management and Accounting* | 10,901 | 4,295 | 706 | 16.44% | 6.48% |
| *Economics, Econometrics and Finance* | 23,712 | 11,964 | 2,329 | 19.47% | 9.82% |
| *Energy* | 14,794 | 5,580 | 489 | 8.76% | 3.31% |
| *Health Professions* | 7,814 | 3,185 | 261 | 8.19% | 3.34% |
| *Immunology and Microbiology* | 7,400 | 3,077 | 320 | 10.40% | 4.32% |
| *Medicine* | 63,391 | 23,848 | 2,735 | 11.47% | 4.31% |
| *Multidisciplinary* | 20,113 | 8,306 | 946 | 11.39% | 4.70% |
| *Neuroscience* | 5,531 | 2,268 | 267 | 11.77% | 4.83% |
| *Nursing* | 4,864 | 1,942 | 273 | 14.06% | 5.61% |
| *Pharmacology, Toxicology and Pharmaceutics* | 3,657 | 1,239 | 152 | 12.27% | 4.16% |
| *Psychology* | 15,295 | 6,615 | 994 | 15.03% | 6.50% |
| *Social Sciences* | 31,632 | 13,711 | 2,360 | 17.21% | 7.46% |
| Total | 220,923 | 91,346 | 12,720 | 13.93% | 5.76% |

*Notes*. Scientific areas are based on the Scimago journal classification. In addition, 36 scientists received a web invitation for a total of 12,756 responses. Of these, 18 responses were excluded as these participants started the survey after the 3^rd^ of June, 2020.

# Sample characteristics

**Supplementary Table 17. Descriptive statistics of sample characteristics**

| Demographic characteristics | N | % |
| --- | --- | --- |
| **Gender** |  |  |
| Male | 7,218 | 56.67% |
| Female | 5,335 | 41.87% |
| Other/Prefer not to say | 185 | 1.46% |
|  |  |  |
| **Age** |  |  |
| 18*–*29 | 577 | 4.53% |
| 30*–*39 | 4,131 | 32.44% |
| 40*–*49 | 3,637 | 28.56% |
| 50*–*59 | 2,248 | 17.65% |
| 60*–*69 | 1,341 | 10.53% |
| >70 | 639 | 5.02% |
| Prefer not to say | 165 | 1.28% |
|  |  |  |
| **Region** |  |  |
| Africa | 364 | 2.85% |
| Asia | 995 | 7.78% |
| Europe | 5,408 | 42.3% |
| North America | 4,759 | 37.22% |
| Oceania | 553 | 4.33% |
| South America | 453 | 3.54% |
| Prefer not to say | 253 | 1.98% |
|  |  |  |
| **Field** |  |  |
| Applied Sciences | 6.51 | 6.51% |
| Arts & Humanities | 295 | 2.32% |
| Economic & Social Sciences | 4,901 | 38.48% |
| Health Sciences | 4,851 | 38.09% |
| Natural Sciences | 1,710 | 13.43% |
| Not specified | 150 | 1.18% |
|  |  |  |
| **Professorship** |  |  |
| Assistant Professor (or below) | 6,664 | 52.8% |
| Associate Professor  (or equivalent) | 2,327 | 18.44% |
| Full Professor (or above) | 3,630 | 28.76% |
| Not specified | 115 | 0.9% |
|  |  |  |
| **Political View** |  | *M (SD)* |
| Liberal to Conservative (7-point scale) | 8,287 | 2.79 (1.35) |
|  |  |  |
| **Marital Status** |  |  |
| Married | 5,634 | 44.24% |
| De facto partnered | 1,288 | 10.11% |
| Divorced | 321 | 2.52% |
| Separated | 124 | 0.97% |
| Widowed | 75 | 0.59% |
| Single | 1,157 | 9.08% |
| Not specified | 4,139 | 32.48% |
|  |  |  |
| **Number of children** |  |  |
| None | 3,160 | 24.81% |
| 1 child | 1,621 | 12.73% |
| 2 children | 2,586 | 20.3% |
| 3 children | 920 | 7.22% |
| 4 children | 224 | 1.76% |
| 5 or more children | 95 | 0.75% |
| Not specified | 4,132 | 32.43% |
|  |  |  |
| **Religious service attendance** |  |  |
| > once a week | 243 | 1.91% |
| Once a week | 838 | 6.58% |
| Once a month | 447 | 3.51% |
| Only on special holy days | 918 | 7.21% |
| Once a year | 518 | 4.07% |
| Less often | 802 | 6.3% |
| Never, practically never | 4,840 | 38% |
| Not specified | 4,132 | 32.43% |

# Replication of main results excluding the *RePEc* sample

In this section, we summarise the results of the analyses in the main text, excluding the respondents drawn from the *RePEc* sample (*n* = 1,019). Overall, the results presented here are quantitatively and qualitatively very similar to those in the main text. For example, the distribution of the overall attitudes towards immunity certification is within 1 percentage point difference (for each question and each response category) when the *RePEc* sample is excluded (Supplementary Figure 18). In general, the statistical differences in opinion across fields are very similar while effect sizes remain small (Supplementary Table 18). Significant differences (both statistical and economical) between US and non-US respondents remain visible when the RePEc sample is excluded (17.3% of the RePEc respondents are from the US) (Supplementary Figure 19). Overall consensus in each question increased slightly when focusing on the Scopus sample (Supplementary Figure 20). Lastly, excluding the *RePEc* sample has a negligible effect on the regression estimates (Supplementary Figure 21).


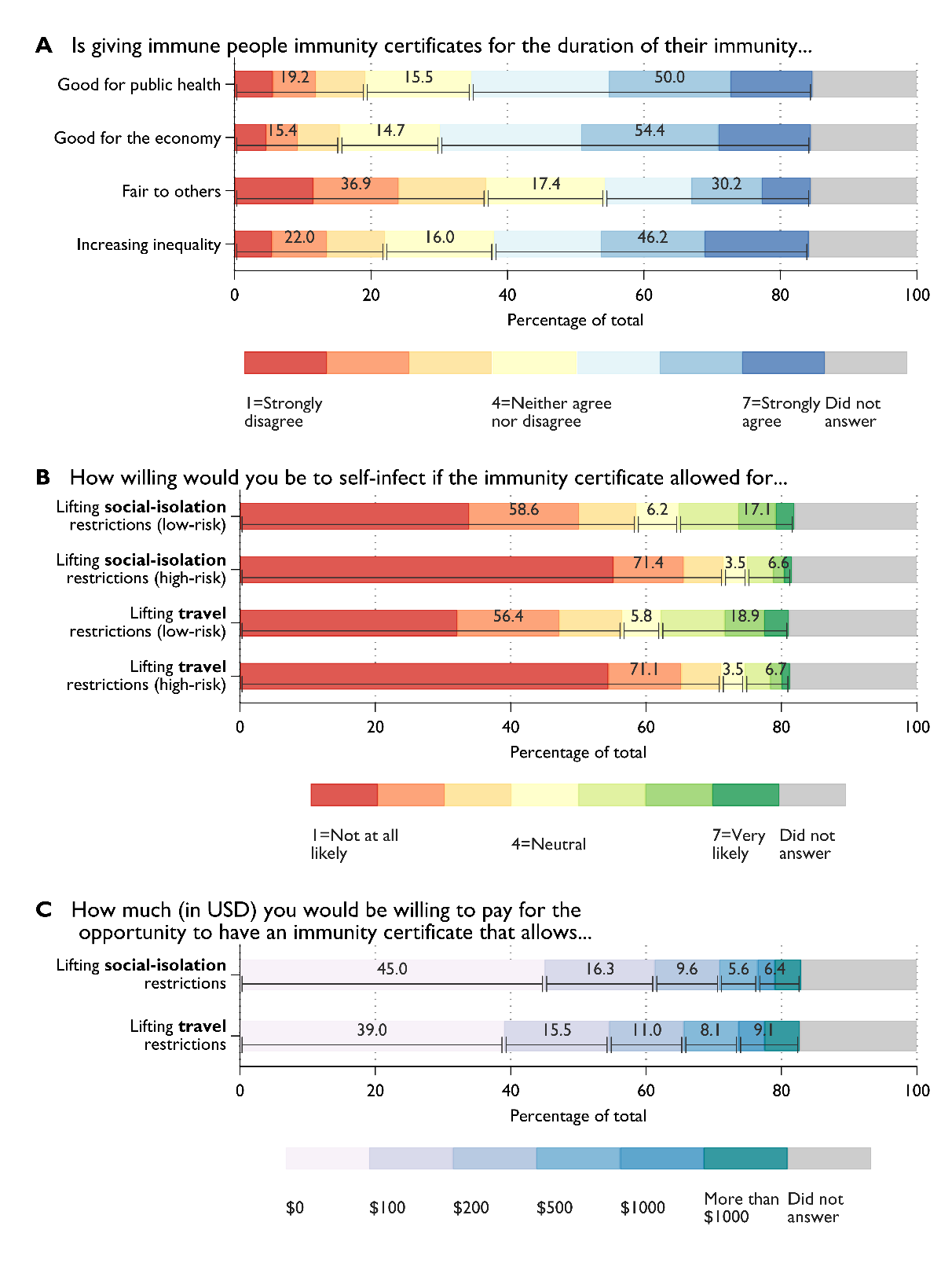
 **Supplementary Figure 18. Scientists’ attitudes towards immunity certificates, excluding *RePEc* sample.** *N* = 11,719 participants. *(A)* Distribution of responses to the statement “Is giving immune people immunity certificates for the duration of their immunity...” regarding 1) good for public health; 2) good for the economy; 3) fair to others who do not have immunity; and 4) increasing inequality. *(B)* Willingness to self-infect for immunity certificate that lifts *social-isolation* and *travel* restrictions, if medically assessed as *low-risk* or *high-risk* groups. *(C)* Willingness to pay for immunity certificate that lifts *social-isolation* and *travel* restrictions.

**Supplementary Table 18. Differences in opinion towards COVID-19 immunity certificates across fields, excluding *RePEc* sample.**

|  |  |  | *Good for public health* | | | *Good for the economy* | | | *Fair to others* | | | *Increase inequality* | | |
| --- | --- | --- | --- | --- | --- | --- | --- | --- | --- | --- | --- | --- | --- | --- |
|  |  |  | *d* | z-stat. | *p*-val. | *d* | z-stat. | *p*-val. | *d* | z-stat. | *p*-val. | *d* | z-stat. | *p*-val. |
| Applied Sciences | vs. | Arts & Humanities | 0.17 | 2.626* | 0.014 | 0.074 | 1.113 | 0.332 | 0.097 | 1.507 | 0.132 | -0.081 | -1.256 | 1.000 |
| Applied Sciences | vs. | Economic &  Social Sciences | 0.034 | 1.048 | 0.164 | 0.014 | 0.431 | 0.417 | 0.054 | 1.725 | 0.106 | -0.028 | -0.892 | 0.466 |
| Arts & Humanities | vs. | Economic &  Social Sciences | -0.077 | -2.288* | 0.028 | -0.032 | -0.979 | 0.328 | -0.02 | -0.599 | 0.343 | 0.028 | 0.846 | 0.398 |
| Applied Sciences | vs. | Health Sciences | 0.076 | 2.669* | 0.019 | 0.047 | 1.619 | 0.264 | 0.1 | 3.441** | 0.003 | -0.029 | -1.000 | 0.529 |
| Arts & Humanities | vs. | Health Sciences | -0.039 | -1.299 | 0.121 | -0.008 | -0.244 | 0.403 | 0.013 | 0.456 | 0.36 | 0.025 | 0.794 | 0.356 |
| Economic &  Social Sciences | vs. | Health Sciences | 0.066 | 2.837* | 0.023 | 0.049 | 2.093 | 0.182 | 0.069 | 2.991** | 0.007 | -0.004 | -0.163 | 0.435 |
| Applied Sciences | vs. | Natural Sciences | 0.062 | 1.413 | 0.113 | 0.029 | 0.675 | 0.357 | 0.088 | 2.075† | 0.063 | -0.019 | -0.413 | 0.378 |
| Arts & Humanities | vs. | Natural Sciences | -0.091 | -1.872† | 0.061 | -0.037 | -0.742 | 0.382 | -0.011 | -0.222 | 0.412 | 0.052 | 1.073 | 0.708 |
| Economic &  Social Sciences | vs. | Natural Sciences | 0.019 | 0.686 | 0.246 | 0.012 | 0.42 | 0.375 | 0.021 | 0.763 | 0.318 | 0.017 | 0.574 | 0.354 |
| Health Sciences | vs. | Natural Sciences | -0.038 | -1.432 | 0.127 | -0.03 | -1.144 | 0.421 | -0.038 | -1.471 | 0.118 | 0.019 | 0.716 | 0.339 |
| N |  |  | 9,928 |  |  | 9,901 |  |  | 9,899 |  |  | 9,863 |  |  |

*Notes*. Cohen’s *d* $\text{= 2*}\text{z}\text{/}\sqrt{n}$. Significance levels: ****p* < .001, ***p* < .01, **p* < .05, †*p* < .1. Non-parametric pairwise multiple comparison (Dunn, 1964) controlling for the false discovery rate using the Benjamini-Hochberg stepwise adjustments.


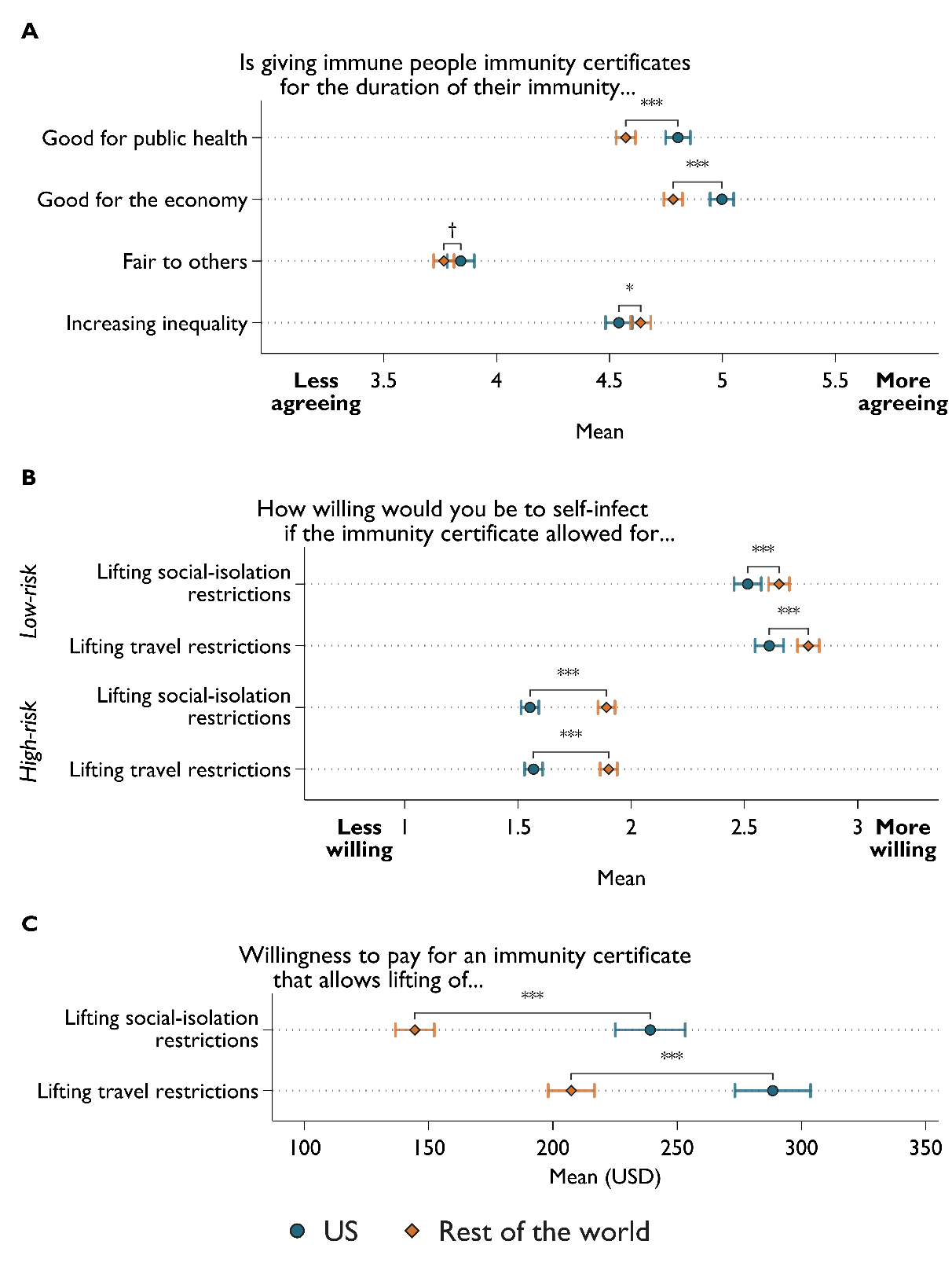


**Supplementary Figure 19. Difference in views on immunity certificates between US and non-US based scientists, excluding *RePEc* sample.** (*A*) Views regarding perceived benefits to *public health* and *economy*, *fairness*, and *societal inequality* of immunity certificate. (*B*) Willingness to pay for immunity certificate for lifting *social-isolation* and *travel restrictions*. (*C*) Willingness to self-infect for immunity certificate that lifts *social-isolation* and *travel* restrictions. Two-sample mean comparison with *t-*test (two-tailed). Error bars represent 95% confidence intervals. Significance levels: ****p* < .001, ***p* < .01, **p* < .05, †*p* < .1. Results are robust to using the Wilcoxon rank sum test (Supplementary Table 4).


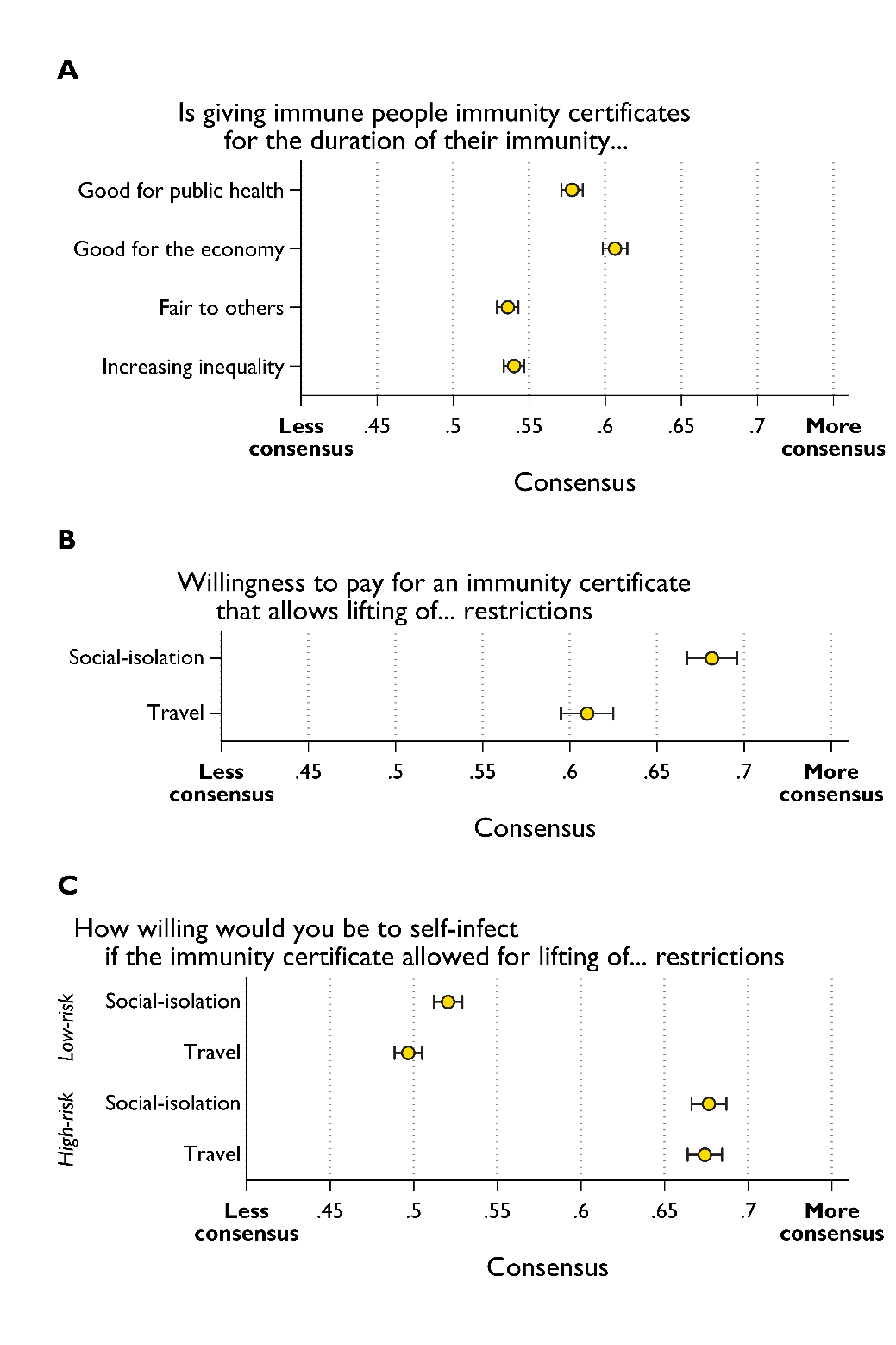


**Supplementary Figure 20. Consensus among scientists, excluding *RePEc* sample.** The entropy-based consensus measure takes the value of 1 when all responses are concentrated on one option and value of 0 when responses are evenly distributed in each available option. (*A*) Views on perceived benefits to *public health* and *economy*, *fairness*, and *societal inequality* of immunity certificate. (*B*) Willingness to pay for immunity certificate that lifts *social-isolation* and *travel restrictions*. (*C*) Willingness to self-infect for immunity certificate that lifts *social-isolation* and *travel restrictions*. Error bars represent 95% confidence intervals obtained from bootstrap resampling with 300 replications. Null responses are excluded from the calculation of consensus.


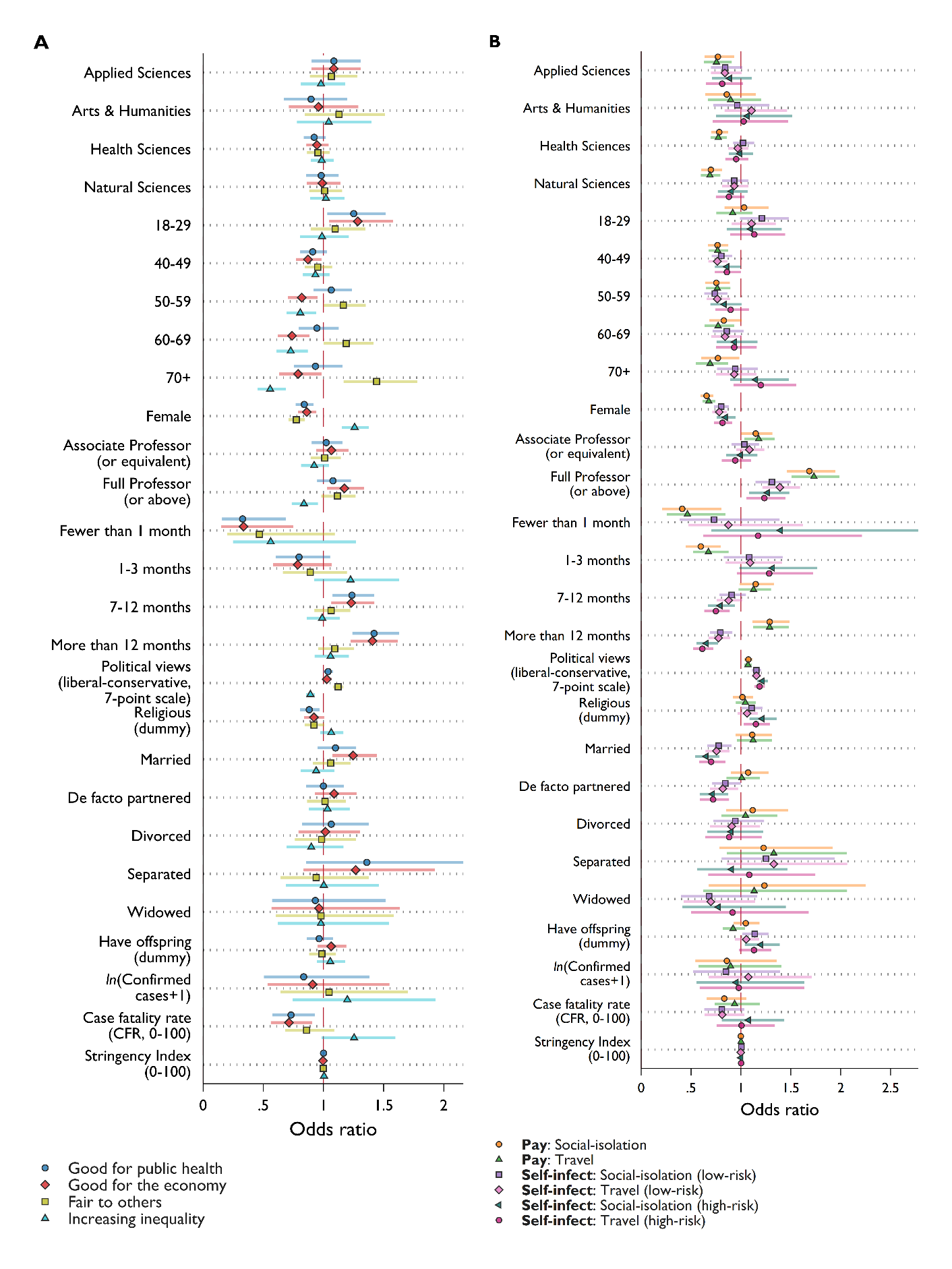


**Supplementary Figure 21. Ordered logit regressions, excluding *RePEc* sample.** Presented are odd ratios of covariates from 10 ordered logit regressions for (*A*) perceived benefits to *public health* and the *economy*, and *fairness* and *inequality* concerns and (*B*) willingness to pay and willingness to self-infect for immunity certificate. Error bars represent 95% confidence intervals.

**Supplementary Data 1. List of journals.** This data file contains the full list of journals from which our sample of scientists was surveyed. These journals ranked top 20 in the SCImago based on SJR (SCImago Journal Rank Indicator 2020) in 55 categories from 13 areas.
